# HIF-2**α** expression and metabolic signaling require ACSS2 in clear cell renal cell carcinoma

**DOI:** 10.1172/JCI164249

**Published:** 2024-06-17

**Authors:** Zachary A. Bacigalupa, Emily N. Arner, Logan M. Vlach, Melissa M. Wolf, Whitney A. Brown, Evan S. Krystofiak, Xiang Ye, Rachel A. Hongo, Madelyn Landis, Edith K. Amason, Kathryn E. Beckermann, W. Kimryn Rathmell, Jeffrey C. Rathmell

**Affiliations:** 1 Department of Medicine,; 2 Department of Pathology, Microbiology, and Immunology, and; 3 Vanderbilt Center for Immunobiology, Vanderbilt University Medical Center, Nashville, Tennessee, USA.; 4 Cell Imaging Shared Resource, Vanderbilt University, Nashville, Tennessee, USA.

**Keywords:** Cell biology, Metabolism, Cancer, Hypoxia, Molecular biology

## Abstract

Clear cell renal cell carcinoma (ccRCC) is an aggressive cancer driven by *VHL* loss and aberrant HIF-2α signaling. Identifying means to regulate HIF-2α thus has potential therapeutic benefit. Acetyl-CoA synthetase 2 (ACSS2) converts acetate to acetyl-CoA and is associated with poor patient prognosis in ccRCC. Here we tested the effects of ACSS2 on HIF-2α and cancer cell metabolism and growth in ccRCC models and clinical samples. ACSS2 inhibition reduced HIF-2α levels and suppressed ccRCC cell line growth in vitro, in vivo, and in cultures of primary ccRCC patient tumors. This treatment reduced glycolytic signaling, cholesterol metabolism, and mitochondrial integrity, all of which are consistent with loss of HIF-2α. Mechanistically, ACSS2 inhibition decreased chromatin accessibility and HIF-2α expression and stability. While HIF-2α protein levels are widely regulated through pVHL-dependent proteolytic degradation, we identify a potential pVHL-independent pathway of degradation via the E3 ligase MUL1. We show that MUL1 can directly interact with HIF-2α and that overexpression of MUL1 decreased HIF-2α levels in a manner partially dependent on ACSS2. These findings identify multiple mechanisms to regulate HIF-2α stability and ACSS2 inhibition as a strategy to complement HIF-2α–targeted therapies and deplete pathogenically stabilized HIF-2α.

## Abstract 2

ACSS2 inhibition promotes HIF-2α degradation via MUL1 to effectively block clear cell renal cell carcinoma in tumor models resistant to HIF-2α inhibitor therapy.

## Introduction

Clear cell renal cell carcinoma (ccRCC) is the most abundant subtype of kidney cancer, accounting for 75% of all cases (1). In ccRCC, a deregulated hypoxia transcriptional response is the major driver and is centrally mediated by hypoxia-inducible factor-2α (HIF-2α) (1–4). HIF-2α is posttranslationally modified and subject to oxygen-dependent hydroxylation to mark the protein for proteasomal destruction mediated by the von Hippel–Lindau (pVHL) E3 ubiquitin ligase. Mutations in the *VHL* gene are common in ccRCC, resulting in HIF-2α stabilization and a pseudohypoxic transcriptional response. HIF-2α regulates transcription of a range of metabolic genes and pathways, including glycolysis and the lipid metabolism that fuels the clear cell phenotype (5, 6), and *VHL*-deficient tumor cells develop a dependence on HIF transcriptional activity. Activity of HIF-2α requires dimerization with HIF-1β and has led to the development of small-molecule inhibitors targeting HIF-2α by interfering with dimerization with its cognate partner, HIF-1β (7). Belzutifan (MK-6482, previously PT-2399) is the first of such molecules to receive FDA approval for the treatment of adult patients with pVHL syndromic tumors (8).

While pVHL-mediated degradation of HIF-α proteins is the most well-understood mechanism of HIF-α disposal, pVHL-independent pathways may also promote protein degradation. Recently, the mitochondrion-tethered E3 ubiquitin ligase MUL1 was found to indirectly regulate HIF-1α protein levels (9). Though MUL1 is best described to target proteins for degradation via SUMOylation, it can also aid mitophagy and autophagic degradation of mitochondria-associated proteins via ubiquitylation (10). Given the similarities with HIF-1α, MUL1 may also regulate HIF-2α.

Because of HIF-2α stabilization, ccRCCs show the greatest in vivo glycolytic flux among tumors tested (11). However, ccRCCs display surprisingly low levels of FDG-PET avidity (12) and preferential consumption of glutamine and lipids (13–15). Clinically, ^11^C-acetate has been reported in renal cancers to provide a more effective PET tracer than standard ^18^F-FDG imaging strategies and may aid in the evaluation of tumor lipid content (16, 17). Indeed, acetate may contribute as much as 50% of the carbon mass during oxygen stress (18). ccRCC is particularly lipid rich, which accounts for the clear cell appearance of this tumor. High lipid content may arise from uptake or synthesis from acetyl-CoA derived by conversion of cytosolic citrate to acetyl-CoA by ATP-citrate lyase (ACLY) or by uptake and conversion of acetate directly to acetyl-CoA by acetyl-CoA synthetase 2 (ACSS2). How lipids or lipid metabolism contribute to the viability of ccRCC cells, or to the opportunities to disrupt cellular homeostasis in this cancer, has been an object of much consideration (19).

Intermediate enzymes of lipid metabolism have been linked to prognosis in ccRCC. Notably, a previous study of cell lines and matched ccRCC patient samples showed that despite decreases in some bulk sequencing data in The Cancer Genome Atlas, ACSS2 was elevated at both the mRNA and protein levels, corresponding to a decline in prognosis (20). ACSS2 expression is elevated in cancer cells during low-oxygen conditions and can contribute to lipid biosynthesis (21). ACSS2 activity has also been linked to the maintenance of HIF-2α signaling output during metabolic stress in other cancer models (22–24). Despite these connections, the role of ACSS2 as a therapeutic target and the potential to regulate the pathogenic expression of HIF-2α or metabolism in ccRCC have been uncertain.

Here we characterize ACSS2 in ccRCC models and explore the relationship between ACSS2 and alternative mechanisms that regulate HIF-2α expression and signaling. Inhibition of ACSS2 downregulated HIF-2α signaling, as well as downstream cholesterol biosynthesis, glucose uptake, and oxidative metabolism. Notably, restoration of ACSS2 expression with a catalytically inactive mutant demonstrated that cell growth and HIF-2α expression are partially dependent on ACSS2 enzymatic activity. Intriguingly, ACSS2 inhibition induced HIF-2α proteasomal degradation even in the absence of pVHL, and this appeared to be mediated by MUL1. Importantly, pharmacological inhibition of ACSS2 effectively blocked cancer cell growth in primary ccRCC samples, in which stabilized HIF-2α is a key driver. Together, these findings demonstrate a role for inhibition of ACSS2 to facilitate degradation of HIF-2α in ccRCC.

## Results

### ccRCC tumors display robust ACSS2 expression in cancer cells.

**Figure 1:**
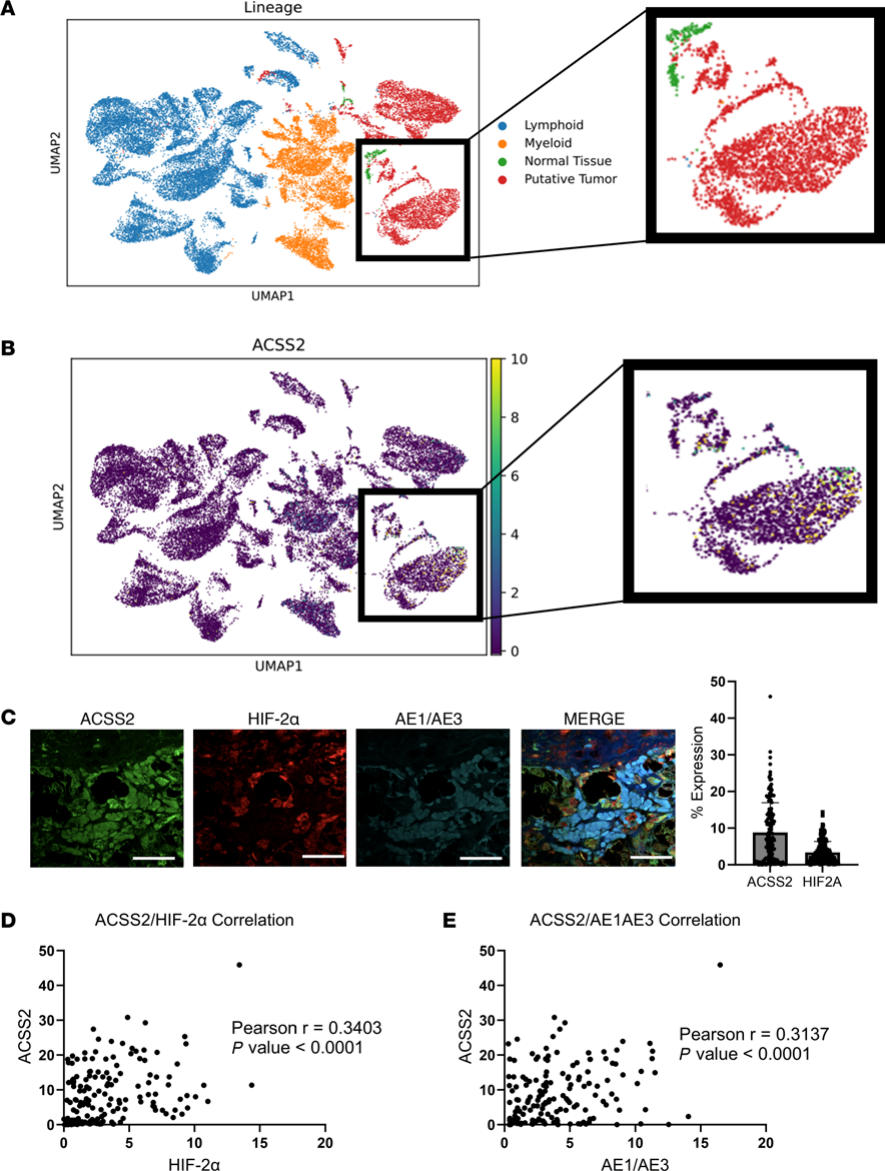
ccRCC tumors have robust ACSS2 expression in cancer cells. (**A**) Uniform manifold approximation and projection (UMAP) plot depicting tumor cell lineage with lymphoid (blue), myeloid (orange), normal tissue (green), and tumor (red) represented. Data were normalized to 10,000 reads per cell using Seurat-Scanpy method, then converted to log scale. Inset focuses on large clusters of normal tissue and tumor. (**B**) UMAP plot (same as in **A**) depicting ACSS2 expression with intensity scored from 0 (purple) to 10 (yellow). Inset focuses on large clusters of normal tissue and tumor. (**C**) Representative images from tissue microarray immunofluorescence staining for ACSS2 (green), HIF-2α (red), AE1/AE3 (cyan), and merge with DAPI (blue). Scale bars: 20 μm. Bar graph shows the expression of ACSS2 and HIF-2α as a percentage of each respective sample (*n* = 159). (**D**) Pearson’s correlation plot for ACSS2 and HIF-2α. (**E**) Pearson’s correlation plot for ACSS2 and AE1/AE3.

ACSS2 is a key regulatory enzyme that has been detected in ccRCC tumors and cell lines (20). ccRCC tumors exhibit significant cellular heterogeneity, which includes a significant variety of non-cancerous stromal and immune cells (25–27). We accessed a recently published human ccRCC single-cell RNA sequencing (scRNA-Seq) data set (28) with more refined cell populations and evaluated ACSS2 gene expression. Our analysis found that ACSS2 was primarily expressed in cancer cells in ccRCC tumors (Figure 1, A and B). To further analyze the expression of ACSS2 in ccRCC, we performed immunofluorescent (21) staining on a tissue microarray with 159 ccRCC patient samples. ACSS2 expression correlated with an epithelial marker indicating ccRCC cells, AE1/AE3, and HIF-2α (Figure 1, C–E). These data show that ACSS2 was expressed in the cancer cells of ccRCC tumors, and its expression correlated with epithelial cell markers and the canonical expression of HIF-2α.

### ACSS2 is essential for ccRCC growth and proliferation.

ACSS2 is emerging as a cancer target but has remained largely unexplored in ccRCC. ACSS2 plays a role to generate cytosolic acetyl-CoA from available acetate, and alteration of ACSS2 activity should be reflected in the abundance of acetate. Using both pharmacological and genetic approaches to target ACSS2, we measured the concentration of acetate in the growth medium of 786-O cells. We observed a buildup of acetate in the medium when cells were treated with a pharmacological inhibitor of ACSS2 (ACSS2i) or transduced with shACSS2. Conversely, ACSS2 overexpression depleted acetate levels (Supplemental Figure 1, A and B; supplemental material available online with this article; https://doi.org/10.1172/JCI164249DS1). These observations demonstrate that the targeting strategies effectively altered ACSS2 enzymatic activity as anticipated.

**Figure 2:**
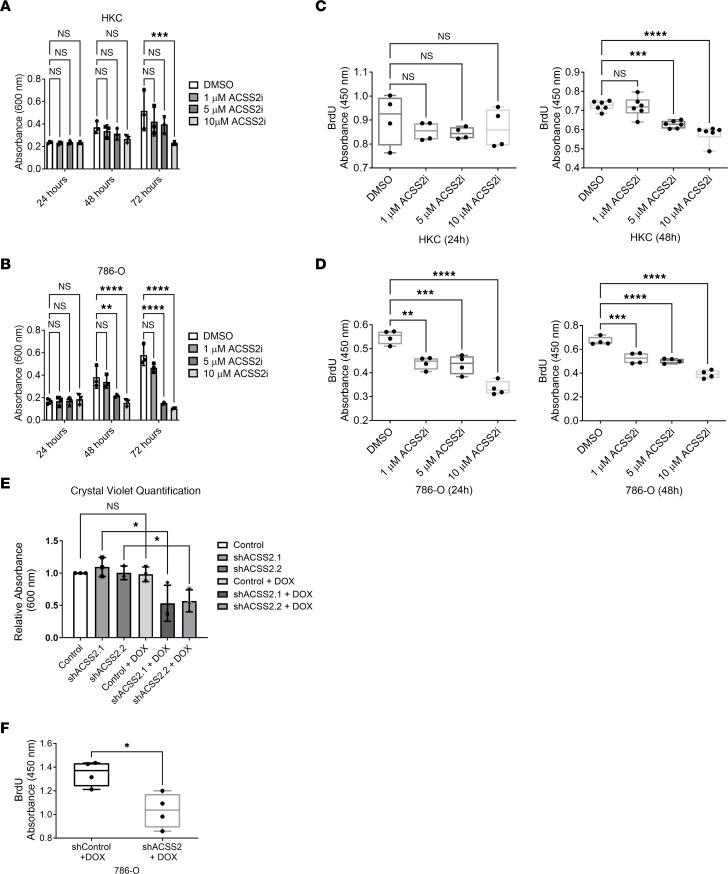
ACSS2 is essential for ccRCC growth and proliferation. (**A** and **B**) Bar graph quantification of 3 independent replicates of crystal violet growth assay performed in HKC cells (**A**) or 786-O cells (**B**). Statistical significance was determined using Tukey’s multiple-comparison test (**P < 0.01; ***P < 0.001; ****P < 0.0001). (**C** and **D**) Box-and-whisker plots showing the absorbance values detected at OD_450nm_ of BrdU ELISA assays performed on HKC cells (**C**) or 786-O cells (**D**) treated for 24 hours (left) or 48 hours (right) with DMSO or 1 μM, 5 μM, or 10 μM ACSS2i (*n* = 4). Statistical significance was determined using Bonferroni’s multiple-comparison test (**P < 0.01; ***P < 0.001; ****P < 0.0001). (**E**) Bar graph quantification of crystal violet staining from 3 independent experiments using 786-O pTRIPZ control or pTRIPZ shACSS2 cells left untreated or treated with 2 μg/mL doxycycline for 48 hours stained with crystal violet. Statistical significance was determined using an unpaired, 2-tailed *t* test (*P < 0.05). (**F**) Box-and-whisker plots showing absorbance values detected at OD_450nm_ of BrdU ELISA assays performed on 786-O pTRIPZ control or 786-O pTRIPZ shACSS2 cells treated with 2 μg/mL doxycycline for 24 hours (*n* = 4). Two-tailed paired *t* test was used to assess statistical significance (*P < 0.05). See also Supplemental Figure 1.

We next performed a dose-response, time-course study with ACSS2i in control HKC (a proximal kidney tubule immortalized cell line) or pVHL-deficient RCC 786-O and A498 cell lines. The viability of HKC cells was only affected at the highest dose, but ACSS2 inhibition led to a significant reduction in cell growth for both pVHL-deficient ccRCC cell lines (Figure 2, A and B, and Supplemental Figure 1C). We next analyzed the effect of ACSS2 inhibition on proliferation by measuring BrdU incorporation. Similarly, while HKC cells were only affected after 48 hours at 5 μM and 10 μM doses, proliferation was significantly reduced in ccRCC cell lines at 24 hours with all doses of the inhibitor (Figure 2, C and D, and Supplemental Figure 1D). 3D growth as tumor spheres using the same cell lines also revealed reduced ability of A498 and 786-O cells to form spheres when treated with ACSS2i (Supplemental Figure 1, E and F). To directly assess the role of ACSS2 in ccRCC growth, we transduced the 786-O cell line with a doxycycline-inducible shRNA construct targeting a control sequence or ACSS2. After 48 hours, we assessed cell growth via crystal violet staining and observed significant reduction in cell growth resulting from ACSS2 depletion (Figure 2E). As with the inhibitor, after 24 hours, 786-O cells expressing shACSS2 displayed significantly reduced BrdU incorporation (Figure 2F). These results show that ACSS2 inhibition can be an effective strategy to block ccRCC growth in vitro.

### ACSS2 is essential for tumor growth in vivo.

**Figure 3:**
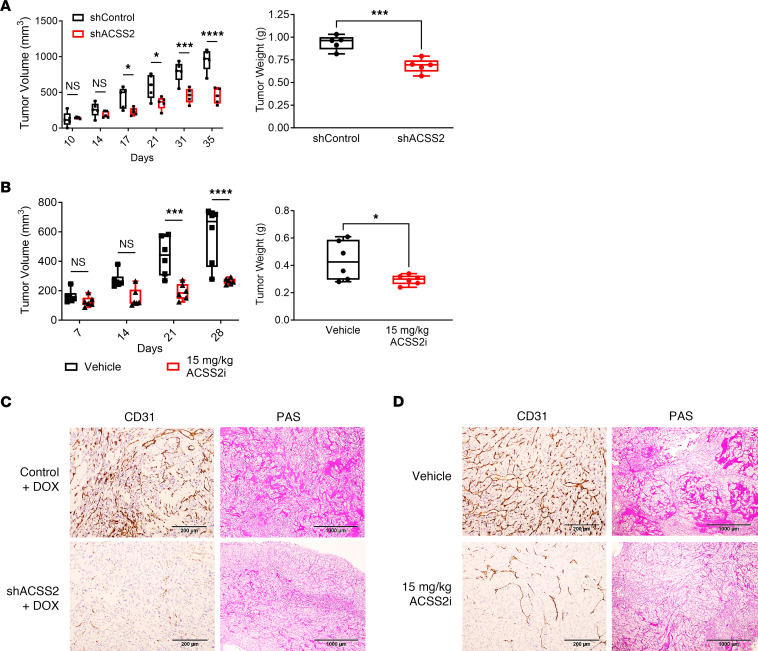
ACSS2 is essential for tumor growth in vivo. (**A** and **B**) Box-and-whisker plots showing individual data points for tumor volume over time and tumor weight in mice inoculated with 786-O pTRIPZ control (black, *n* = 5) or 786-O pTRIPZ shACSS2 (red, *n* = 5) cells and fed a doxycycline rodent chow or treated with vehicle (black, *n* = 6) or 15 mg/kg ACSS2i (red, *n* = 6). Statistical significance was determined using 2-way ANOVA and Šidák’s test for multiple comparisons (*P < 0.05; ***P < 0.0005; ****P < 0.0001). Tumor weight statistical significance was determined using an unpaired, 2-tailed *t* test (***P < 0.001). (**C** and **D**) Representative images taken at ×10 magnification of sections from tumors treated with control or shACSS2 and vehicle or 15 mg/kg ACSS2i and stained with CD31 (left) and periodic acid–Schiff (PAS, right). Scale bars: 200 μm (left), 1,000 μm (right). Data are represented as mean ± SD. See also Supplemental Figure 1.

The effect of ACSS2 inhibition on tumor growth was next tested in vivo. Doxycycline induction of shACSS2 in mice bearing subcutaneous 786-O tumors resulted in a significant reduction in tumor growth and final tumor weight (Figure 3A). Similarly, mice bearing subcutaneous 786-O tumors receiving daily treatment with ACSS2i exhibited a decrease in tumor growth and final tumor weight (Figure 3B). In neither model did targeting ACSS2 result in observable toxicity such as weight loss, suggesting that targeting of ACSS2 is tolerable (Supplemental Figure 1, G and H). Immunohistochemistry for CD31 and periodic acid–Schiff staining revealed that targeting ACSS2 reduced vascularization and glycogen deposition (Figure 3, C and D, and quantified in Supplemental Figure 1, I and J), which are commonly associated clinical features of ccRCC linked to HIF signaling. Together, these data show that ACSS2 contributes to tumor growth and suggest a druggable vulnerability in ccRCC.

### HIF-2α expression and degradation are regulated by ACSS2 activity.

**Figure 4:**
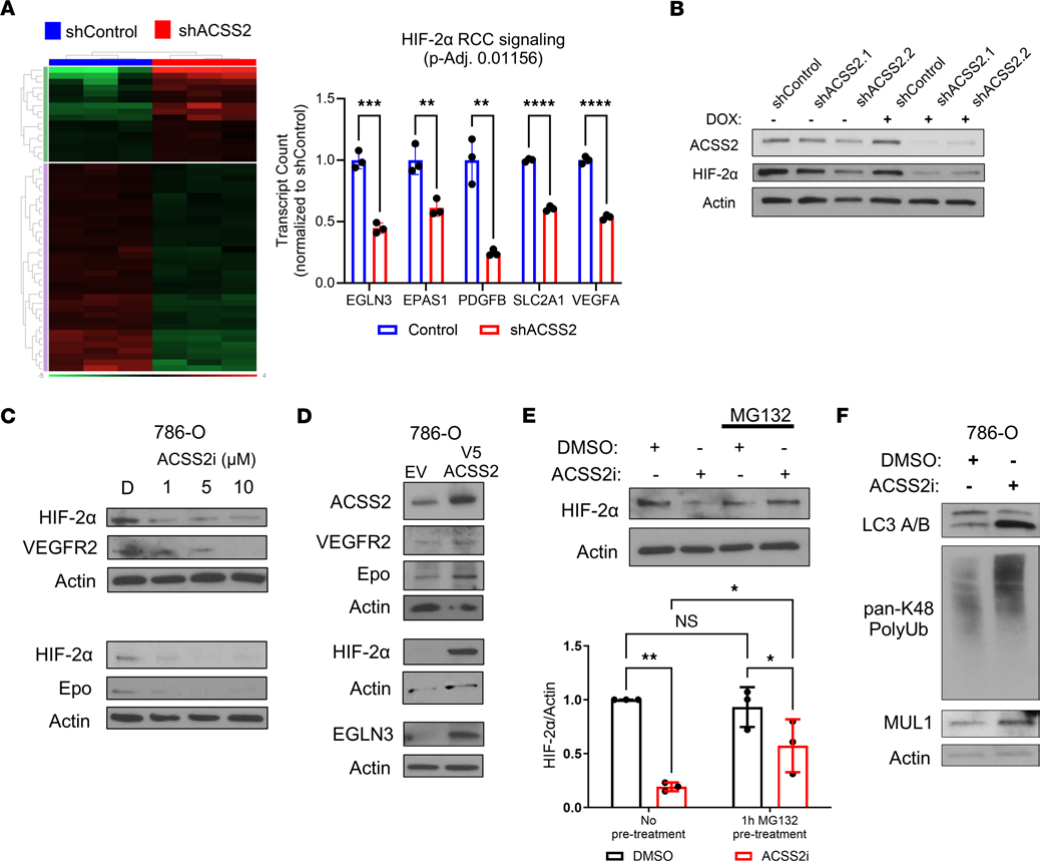
HIF-2α expression and degradation are regulated by ACSS2 activity. (**A**) Heatmap showing differential gene expression expressed as fold change (downregulated or upregulated at least 1.5-fold, adjusted *P* value ≤ 0.05) between control (blue; *n* = 3) and shACSS2 (red; *n* = 3) groups. Bar graph showing relative transcript counts from HIF-2α RCC signaling pathway comparing control (blue) and shACSS2 (red) groups. Statistical significance was determined using multiple unpaired, 2-tailed *t* tests (**P < 0.01; ***P < 0.001; ****P < 0.0001). (**B**) Representative Western blot analysis of HIF-2α (*n* = 3), ACSS2 (*n* = 3), and actin (*n* = 3) expression following 24-hour doxycycline induction of 786-O pTRIPZ control and pTRIPZ shACSS2 cells. (**C**) Representative Western blot analysis assessing expression of HIF-2α (*n* = 3), VEGFR2 (*n* = 2), Epo (*n* = 2), and actin (*n* = 3) in 786-O cells treated with DMSO or 1 μM, 5 μM, or 10 μM ACSS2i for 24 hours. (**D**) Representative Western blot analysis showing expression of ACSS2 (*n* = 6), HIF-2α (*n* = 4), VEGFR2 (*n* = 3), Epo (*n* = 3), EGLN3 (*n* = 3), and actin (*n* = 6) in 786-O cells transduced with pLX304 empty vector or pLX304 V5-ACSS2 overexpression vector. (**E**) Representative Western blot and bar chart quantification (*n* = 3) depicting HIF-2α expression in 786-O cells in the absence or presence of a 1-hour incubation with 10 μM MG132 prior to treatment with DMSO or 5 μM ACSS2i for 24 hours. (**F**) Western blot images analyzing LC3 A/B (*n* = 3), K48 polyubiquitination (*n* = 3), and MUL1 (*n* = 3) in 786-O cells treated with DMSO or 5 μM ACSS2i for 48 hours. Statistical significance was determined using 2-way ANOVA and Holm-Šidák test for multiple comparisons (*P < 0.05; **P < 0.005). Data are represented as mean ± SD. See also Supplemental Figure 2.

The distinctive biology of RCC is largely orchestrated by metabolic changes driven by pathogenic HIF-2α signaling. To test the role of ACSS2 to regulate essential metabolic signaling pathways that promote ccRCC growth, we performed transcriptomic analysis using the NanoString Metabolic Pathways probe set on 786-O cells transduced with shRNA targeting a control sequence or shACSS2 (Figure 4A). Differential expression analysis of this data set identified a HIF-2α RCC gene set consisting of *EGLN3*, *PDGFB*, *SLC2A1*, and *VEGFA*, as well as the gene that encodes HIF-2α (*EPAS1*). This reflected the most significantly downregulated pathway in response to ACSS2 deficiency, suggesting that the core features of HIF-2α transcriptional activation had been disrupted. We further observed reduced HIF-2α protein expression when ACSS2 was targeted with shRNA (Figure 4B). Mirroring the previous results, treatment of 786-O cells with ACSS2i for 24 hours resulted in reduced expression of HIF-2α, as well as HIF-2α targets VEGFR2 and Epo (Figure 4C). Next, we wanted to test whether overexpressing ACSS2 would be sufficient to drive HIF-2α expression and signaling. 786-O cells were stably transduced to express HIF-2α, and immunoblot analysis showed a marked increase in the expression of HIF-2α and its downstream targets VEGFR2, Epo, and EGLN3 (Figure 4D). Proliferation of 786-O cells stably overexpressing ACSS2 also increased as indicated by a significant increase in BrdU incorporation in comparison with control cells (Supplemental Figure 2A).

Given the effects of ACSS2 deficiency on gene expression and cell proliferation, we next sought to evaluate the role of ACSS2 in tumor cell growth and to explore the contribution of HIF-2α to the proliferation phenotype. To this end, shRNA was used to target HIF2A in 786-O control cells (Supplemental Figure 2B) or 786-O cells stably overexpressing ACSS2 (Supplemental Figure 2C). These cells were seeded into an anchorage-independent growth assay, where colony formation was monitored. After 21 days of growth, we observed a significant increase in colony formation when ACSS2 was overexpressed, which was prevented with cotransduction of shHIF2A (Supplemental Figure 2D). Together, these data show that ACSS2 promotes HIF-2α transcription and protein expression, and that HIF activity is essential for ACSS2-enhanced cancer cell growth.

The HIF family of transcription factors are subject to strict regulation at the protein level. While this occurs primarily through the action of pVHL, we observed dynamic changes in protein levels of HIF-2α in pVHL-null cells. We sought to determine, therefore, whether ACSS2 may also regulate HIF-2α protein stability independent of pVHL. We performed a 1-hour incubation with the proteasomal inhibitor MG132 followed by the addition of DMSO or ACSS2i for 24 hours in 786-O cells. Interestingly, 786-O cells pretreated with MG132 prior to ACSS2i displayed a significant rescue in HIF-2α expression (Figure 4E), suggesting that ACSS2 inhibition promotes HIF-2α degradation via a proteasomal mechanism, even in cells lacking *VHL*. Supporting these findings, treatment with ACSS2i led to elevated LC3 A/B processing and pan-K48 polyubiquitination, indicating activation of autophagy and increased proteasomal degradation, respectively (Figure 4F). Together, these data suggest that in the setting of ACSS2 inhibition, HIF-2α protein stability is reduced in part as a result of increased proteasomal degradation and this occurs in the absence of pVHL.

### MUL1 engages with HIF-2α and displays inverse expression.

**Figure 5:**
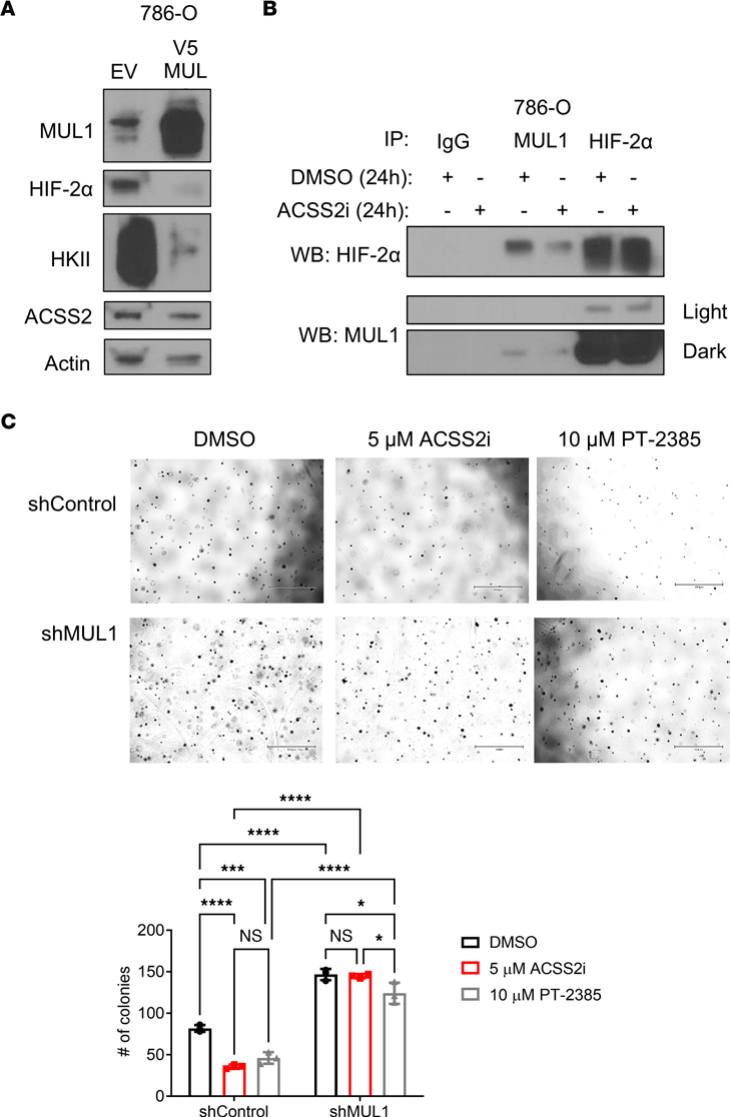
MUL1 engages with HIF-2α and displays inverse expression. (**A**) Western blot analysis showing expression of ACSS2 (*n* = 2), HIF-2α (*n* = 2), HKII (*n* = 2), and MUL1 (*n* = 2) in response to MUL1 overexpression. (**B**) Western blot images following immunoprecipitations of HIF-2α or MUL1 performed on 786-O cells treated with DMSO or 5 μM ACSS2i for 24 hours (*n* = 2). (**C**) Representative images at day 16 of anchorage-independent growth assays (*n* = 3) performed on 786-O cells transduced to express shControl or shMUL1 and treated with either DMSO, 5 μM ACSS2i, or 10 μM PT-2385, and bar graph quantification. Scale bars: 1,050 μm. Statistical significance was determined using 2-way ANOVA and Tukey’s multiple-comparison test (*P < 0.05; ***P < 0.0005; ****P < 0.0001). See also Supplemental Figures 2 and 3.

Recent studies have linked the E3 ubiquitin ligase MUL1 to the regulation of HIF-1α via degradation directed by the E3 ligase cofactor UBXN7 (9). Considering the functional homology between HIF-1α and HIF-2α, we tested whether MUL1 contributed to ACSS2-dependent regulation of HIF-2α protein stability. We hypothesized that MUL1 may bind and target HIF-2α for degradation. Ectopic overexpression of MUL1 in 786-O cells depleted HIF-2α protein levels and reduced expression of hexokinase II (HKII), a downstream target of HIF-2α that is responsible for the committed step in glycolysis (Figure 5A). To test whether MUL1 could interact with HIF-2α, MUL1 was immunoprecipitated from 786-O cells treated with DMSO or ACSS2i for 24 hours. We found that HIF-2α and MUL1 coprecipitated in this assay, and that this interaction was preserved in the presence of ACSS2i (Figure 5B). To confirm that the interaction between MUL1 and HIF-2α was relevant in additional settings, we repeated the immunoprecipitations using A498 cells treated with DMSO or ACSS2i, and tumor cells freshly isolated from a patient tumor. In the latter case, we treated with DMSO, ACSS2i, or PT-2385, a HIF-2α inhibitor that disrupts transcriptional activity. In each case, MUL1 and HIF-2α were found to interact (Supplemental Figure 3, A and B). Lastly, we tested whether the growth defects caused by ACSS2 or HIF-2α inhibition could be neutralized by targeting of MUL1. Intriguingly, depleting MUL1 via shRNA enhanced HIF-2α protein expression and provided 786-O cells with resistance to treatment with either ACSS2i or PT-2385, enabling successful anchorage-independent growth (Figure 5C and Supplemental Figure 2E). Our data demonstrate that ACSS2 regulates HIF-2α protein degradation and suggest a conserved and actionable HIF-2α regulatory axis involving ACSS2 and MUL1, which may complement ongoing efforts to target the HIF-2α transcriptional complex.

### ACSS2 activity supports cholesterol biosynthesis and glucose uptake.

**Figure 6:**
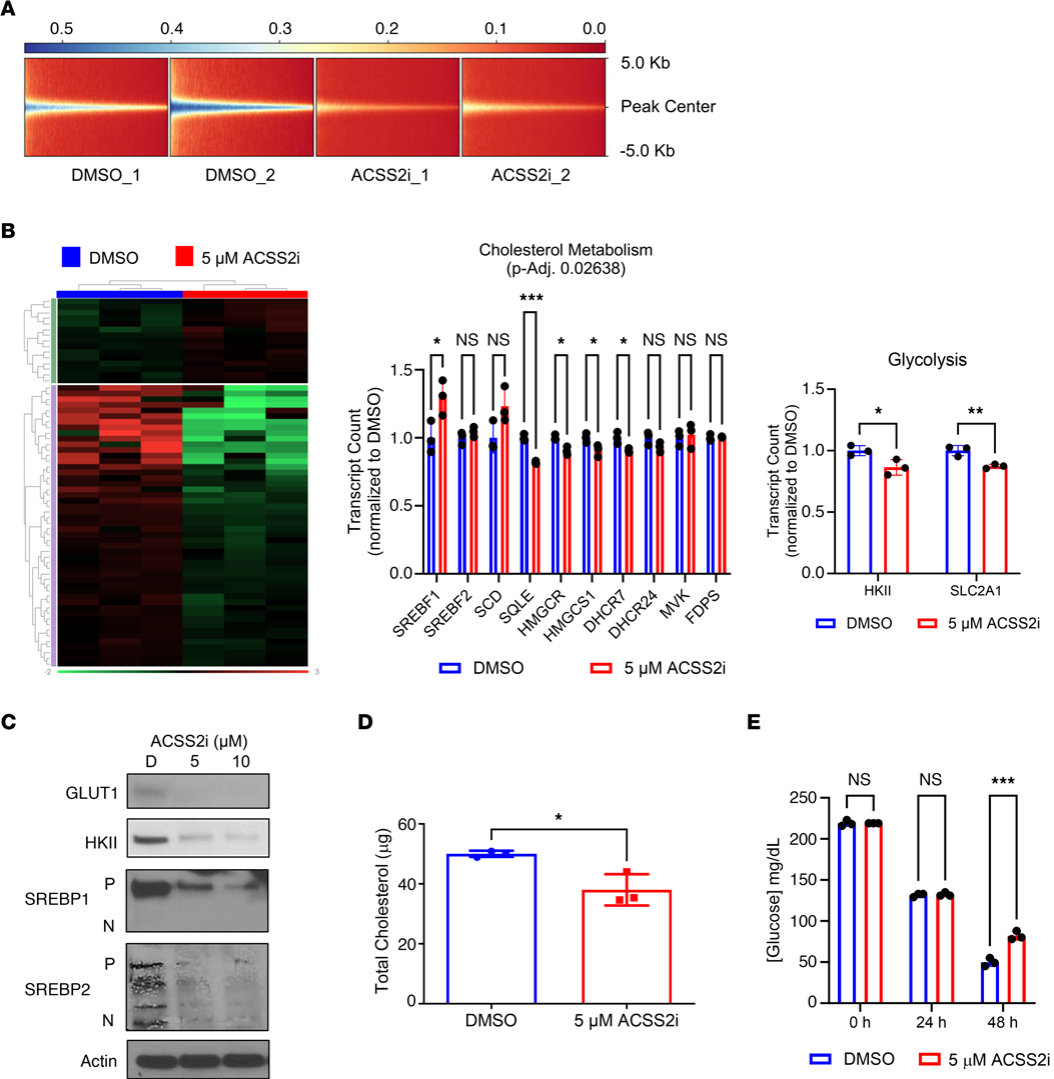
ACSS2 activity supports cholesterol biosynthesis and glucose uptake. (**A**) Heatmap showing gene distance from the transcription start site as a measure of accessibility (red, no coverage; blue, maximum coverage). (**B**) Left: Heatmap showing differential gene expression expressed as fold change (downregulated or upregulated at least 1.1-fold, adjusted *P* value ≤ 0.05) between groups with 24-hour treatment of 786-O cells with DMSO (blue; *n* = 3) versus 5 μM ACSS2i (red; *n* = 3). Gene expression clustering on the *y* axis depicts genes that are upregulated (purple) and downregulated (green) in the DMSO group. Middle and right: Bar graphs showing transcript counts for genes involved in the cholesterol biosynthesis pathway and glycolysis. Statistical significance was determined by multiple unpaired, 2-tailed *t* tests (*P < 0.05; **P < 0.005; ***P < 0.0005). (**C**) Western blot analysis assessing expression of GLUT1 (*n* = 2), HKII (*n* = 2), SREBP1 (*n* = 2), SREBP2 (*n* = 2), and actin (*n* = 3) in 786-O cells treated with DMSO or 5 μM or 10 μM ACSS2i for 24 hours. (**D**) Bar graph showing total cholesterol levels in 786-O cells treated with DMSO (blue; *n* = 3) or 5 μM ACSS2i (red; *n* = 3) for 24 hours. Statistical significance was determined using an unpaired, 2-tailed Student’s *t* test (*P < 0.05). (**E**) Bar graph showing measured glucose concentrations in the medium of 786-O cells treated with DMSO or 5 μM ACSS2i for 0, 24, and 48 hours (*n* = 3 for all conditions). Statistical significance was determined using a 2-way ANOVA and Tukey’s multiple-comparison test (***P < 0.0005). Data are represented as mean ± SD.

Acetate metabolism intersects metabolic and epigenetic regulation by contributing to the acetyl-CoA pool. We next sought to determine how ACSS2 deficiency affected RCC cells. Having demonstrated that ACSS2 activity regulates the transcription of HIF-2α and its immediate transcriptional targets, we next hypothesized that changes in acetate metabolism and acetyl-CoA levels would have broad effects on chromatin accessibility. We performed assay for transposase-accessible chromatin sequencing (ATAC-seq) on 786-O cells treated with DMSO or ACSS2i for 24 hours and observed a distinctive loss of chromatin accessibility associated with inhibition of ACSS2 (Figure 6A).

We next assessed the impact of ACSS2i treatment on the transcriptome of a broader panel of metabolic pathways by performing the NanoString Metabolic Pathways assay in 786-O cells treated with DMSO or ACSS2i for 24 hours. Differential gene expression analysis identified cholesterol metabolism as the most significantly altered pathway in 786-O cells treated with ACSS2i (Figure 6B). Additionally, we confirmed that ACSS2 inhibition significantly reduced the HIF-2α target transcripts that encode hexokinase II (*HK2*, HKII) and glucose transporter 1 (*SLC2A1*, GLUT1), which represent the committed steps to glycolysis. These findings were validated by analysis of changes to protein expression following 24 hours of treatment with DMSO or ACSS2i. Protein levels of GLUT1, HKII, SREBP1, and SREBP2, all of which are established HIF-2α target proteins (29, 30), were decreased in response to ACSS2 inhibition (Figure 6C). Total cholesterol levels were assessed in 786-O cells treated with DMSO or ACSS2i for 24 hours and showed a significant reduction in response to ACSS2 inhibition (Figure 6D). Similarly, a time-course treatment performed in 786-O cells treated with DMSO or ACSS2i led to reduced glucose consumption, reflected by a greater level of glucose remaining in medium after 48 hours of culture (Figure 6E). These results demonstrate that ACSS2 activity regulates the rate-limiting enzymes in cholesterol synthesis and glucose metabolism, both of which are intimately linked to aberrant HIF-2α signaling in ccRCC.

### ACSS2 enzymatic activity is required for optimal growth and HIF-2α expression.

**Figure 7:**
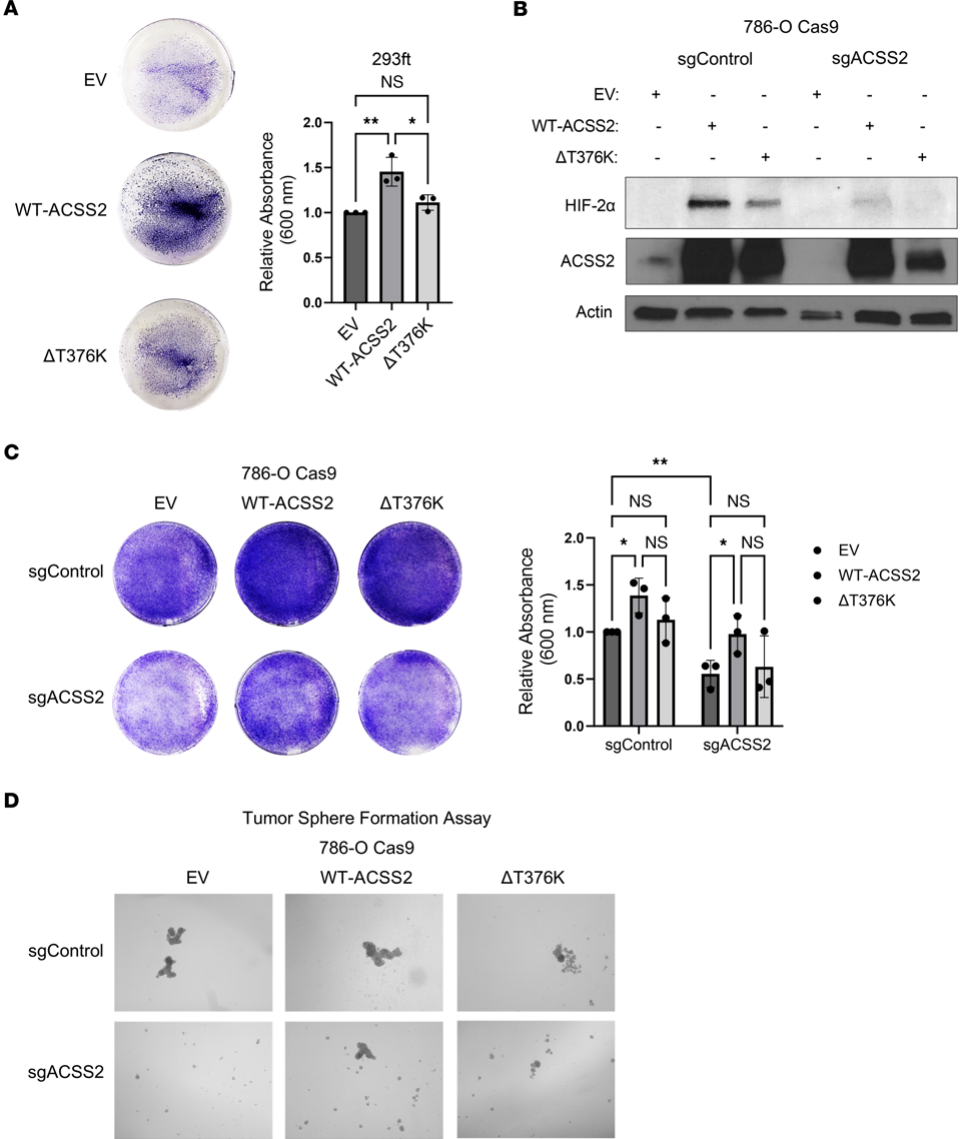
Enzymatic activity of ACSS2 is required for optimal growth and HIF-2α expression. (**A**) Left: Representative images of crystal violet staining of HEK293FT cells 72 hours after transfection with an empty vector control (EV), WT-ACSS2, or ΔT376K catalytically inactive mutant. Right: Bar graph showing quantification of 3 independent experiments in HEK293FT cells. Statistical significance was determined using a 1-way ANOVA and Tukey’s multiple-comparison test (*P < 0.05; **P < 0.01). Data are represented as mean ± SD. (**B**) Western blot images showing expression of HIF-2α (*n* = 3), ACSS2 (*n* = 3), and actin (*n* = 3) in 786-O Cas9 sgControl and sgACSS2 cells transfected with EV, WT-ACSS2, or ΔT376K. (**C**) Left: Representative images of crystal violet staining of 786-O Cas9 cells stably transduced to express sgControl or sgACSS2 72 hours after transfection with an empty vector control (EV), WT-ACSS2, or ΔT376K catalytically inactive mutant. Right: Bar graph showing quantification of 3 independent experiments in 786-O Cas9 cells. Statistical significance was determined using a 2-way ANOVA and Tukey’s multiple-comparison test (*P < 0.05; **P < 0.01). Data are represented as mean ± SD. (**D**) Representative images from day 4 of a tumor sphere formation assay where 786-O Cas9 sgControl and sgACSS2 cells were transfected with EV, WT-ACSS2, or ΔT376K.

We next sought to determine whether regulation of ccRCC growth and HIF-2α signaling by ACSS2 was dependent directly on ACSS2 enzymatic activity. A previously described catalytically inactive ACSS2 mutant was therefore generated by conversion of T376, a residue that confers a CoA binding domain, to a lysine (ΔT376K) (31). First, the effects of expressing WT-ACSS2 and ΔT376K on cell growth were compared in HEK293FT cells. Relative to cells transfected with an empty vector control, overexpression of WT-ACSS2 enhanced cell growth (Figure 7A). To eliminate background of endogenous ACSS2, 786-O Cas9 cells stably transduced with sgControl or sgACSS2 were tested to determine whether ACSS2 enzymatic activity was required for HIF-2α protein expression and cell growth in ccRCC. A limitation to this approach was that WT-ACSS2 and ΔT376K ACSS2 were not equivalently expressed (Figure 7B). Nevertheless, while WT-ACSS2 robustly enhanced HIF-2α expression, the catalytically inactive mutant (ΔT376K) did less so in 786-O Cas9 sgControl and sgACSS2 cells. Likewise, WT-ACSS2 partially rescued HIF-2α expression, yet ΔT376K was unable to fully rescue HIF-2α expression. The requirement for ACSS2 enzymatic activity to facilitate ccRCC cell growth was next tested. 786-O Cas9 sgACSS2 cells displayed significantly reduced cell growth. Restoring expression of ACSS2 with WT-ACSS2 rescued cell growth to cell densities comparable to those of control cells, while expression of ΔT376K failed to rescue growth (Figure 7C). These findings were mirrored in a tumor sphere formation assay, where reconstituting expression of ACSS2 with WT-ACSS2, but not ΔT376K, yielded improved tumor sphere formation (Figure 7D). These data suggest that the enzymatic activity of ACSS2 regulates HIF-2α protein expression to support optimal cell growth.

### Targeting ACSS2 elicits cancer cell–specific mitochondrial defects.

Having demonstrated a role for ACSS2 in the regulation of hypoxia signaling, glycolysis, and cholesterol biosynthesis, we next sought to explore which pathways were most associated with ACSS2 in ccRCC. To address this, we performed a gene set enrichment analysis on the ACSS2-expressing cancer cells from the ccRCC scRNA-Seq data set (28), in which we observed a strong enrichment for metabolic programs, with the top hit being mitochondrial oxidative phosphorylation (OXPHOS) (Supplemental Figure 4A). To explore these metabolic connections more carefully, we analyzed the protein expression of the OXPHOS complexes in pVHL-deficient 786-O cells treated with ACSS2i and observed reduced expression of the OXPHOS complexes (Supplemental Figure 4B). Conversely, ectopic overexpression of ACSS2 in 786-O cells resulted in elevated expression in complexes I and II as well as GLUT1 and HKII (Supplemental Figure 4C). Next, a colorimetric assay that detects both the nuclear-encoded mitochondrial gene SDH-A and the mitochondrial-encoded COX-I was used to assess the impact on mitochondrial biogenesis. Inhibition of ACSS2 resulted in a significant decrease in COX-I in 786-O cells (Supplemental Figure 4D). Together, these data implicate a role for ACSS2 in the regulation of OXPHOS, as well as mitochondrial biogenesis, in ccRCC.

**Figure 8:**
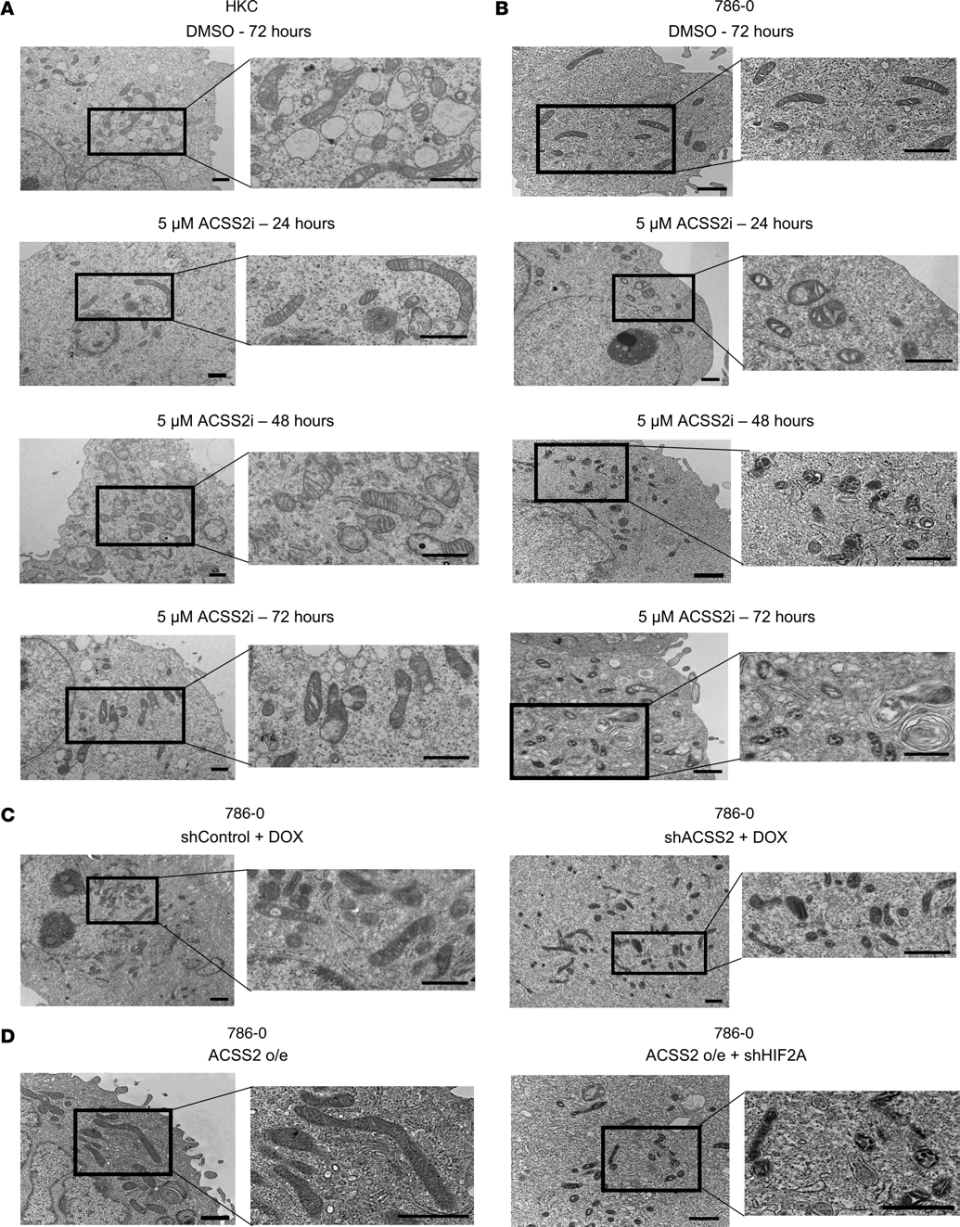
Targeting ACSS2 elicits cancer cell-specific mitochondrial defects. (**A** and **B**) Representative images of HKC control cell mitochondria (**A**) and 786-O mitochondria (**B**) treated with DMSO for 72 hours or with 5 μM ACSS2i for 24, 48, and 72 hours (*n* = 3). (**C**) 786-O pTRIPZ control (*n* = 3) and shACSS2 (*n* = 3) cells induced with 2 μg/mL doxycycline for 48 hours. (**D**) 786-O cells overexpressing ACSS2 transduced with shControl (*n* = 3) or shHIF2A (*n* = 3). Scale bars: 1 μm for all images. See also Supplemental Figure 4.

Quality of mitochondria is intimately linked to their function and ability to facilitate metabolic processes (32, 33). Notably, MUL1 has previously been characterized as having an appreciable role to regulate proteins, such as mitofusin 2, that are involved in mitochondrial dynamics (34, 35). Noting these connections to our results, we hypothesized that inhibiting ACSS2 may selectively disrupt mitochondrial homeostasis. To test the effects on mitochondrial morphology, electron microscopy was performed on HKC and 786-O cells treated with DMSO for 72 hours or with ACSS2i for 24, 48, and 72 hours (Figure 8, A and B). Strikingly, mitochondria in 786-O cells treated with ACSS2i began to fragment as early as 24 hours from the typically observed elongated morphology as seen in the DMSO control, to a smaller and spherical morphology, a process not detected in the mitochondria of HKC cells (Figure 8, A and B). Crista morphology also degenerated from linear and continuous to fragmented and spherical (Figure 8B). To validate this finding with loss of ACSS2, we performed electron microscopy on 786-O cells where shRNA targeting ACSS2 had been induced with doxycycline for 48 hours, and we observed that shACSS2 induction produced the same morphological changes as pharmacological inhibition (Figure 8C).

We next investigated whether this mitochondrial phenotype was due to a general effect of ACSS2 expression or whether it required HIF-2α. To address this, electron microscopy was performed on 786-O cells overexpressing ACSS2 where *HIF2A* was silenced (Figure 8D). While ACSS2 overexpression led to elongated and fused mitochondria, HIF-2α deficiency reversed this phenotype to regulate mitochondrial morphology. Together, these data show that ACSS2 activity intersects with a HIF-2α–dependent role to maintain the quality of mitochondria in *VHL*-mutated cancer cells.

### ACSS2 inhibition selectively impedes cancer cell growth and HIF-2α expression in ccRCC patient samples.

**Figure 9:**
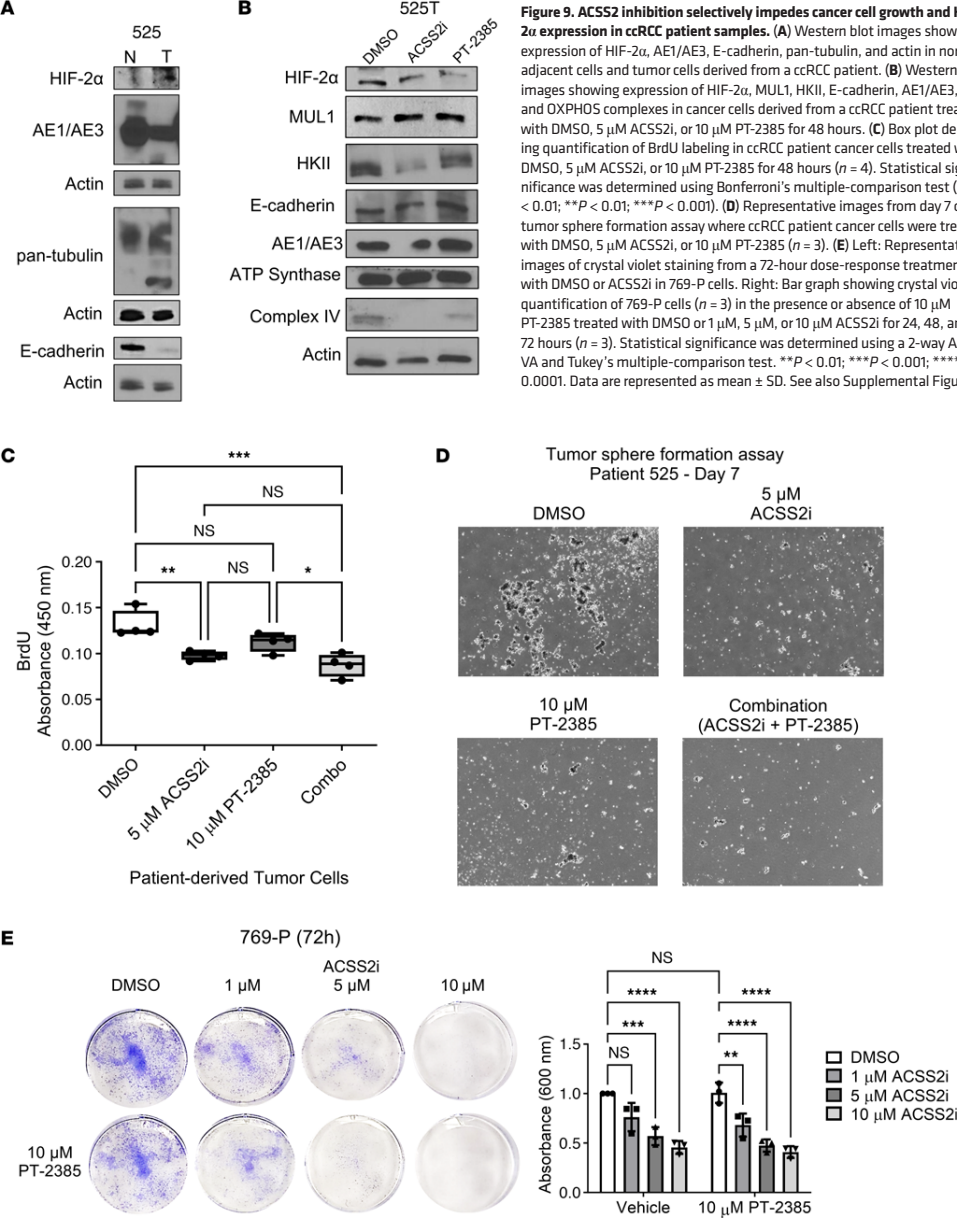
ACSS2 inhibition selectively impedes cancer cell growth and HIF-2α expression in ccRCC patient samples. (**A**) Western blot images showing expression of HIF-2α, AE1/AE3, E-cadherin, pan-tubulin, and actin in normal adjacent cells and tumor cells derived from a ccRCC patient. (**B**) Western blot images showing expression of HIF-2α, MUL1, HKII, E-cadherin, AE1/AE3, and OXPHOS complexes in cancer cells derived from a ccRCC patient treated with DMSO, 5 μM ACSS2i, or 10 μM PT-2385 for 48 hours. (**C**) Box plot depicting quantification of BrdU labeling in ccRCC patient cancer cells treated with DMSO, 5 μM ACSS2i, or 10 μM PT-2385 for 48 hours (*n* = 4). Statistical significance was determined using Bonferroni’s multiple-comparison test (*P < 0.01; **P < 0.01; ***P < 0.001). (**D**) Representative images from day 7 of a tumor sphere formation assay where ccRCC patient cancer cells were treated with DMSO, 5 μM ACSS2i, or 10 μM PT-2385 (*n* = 3). (**E**) Left: Representative images of crystal violet staining from a 72-hour dose-response treatment with DMSO or ACSS2i in 769-P cells. Right: Bar graph showing crystal violet quantification of 769-P cells (*n* = 3) in the presence or absence of 10 μM PT-2385 treated with DMSO or 1 μM, 5 μM, or 10 μM ACSS2i for 24, 48, and 72 hours (*n* = 3). Statistical significance was determined using a 2-way ANOVA and Tukey’s multiple-comparison test. **P < 0.01; ***P < 0.001; ****P < 0.0001. Data are represented as mean ± SD. See also Supplemental Figure 5.

Our findings suggest that ACSS2 may provide a novel therapeutic target in ccRCC. To test whether ACSS2 inhibition had translational effects on primary RCC cells, we compared HIF-2α expression in epithelial cells expanded from the normal adjacent tissue and from the tumor. These samples were of epithelial origin, and the tumor cells had elevated HIF-2α (Figure 9A). We compared the effect of ACSS2 inhibition on HIF-2α expression in freshly isolated cancer cells and found that 24 hours of treatment at all doses of ACSS2i was comparable to PT-2385 treatment to reduce HIF-2α, HKII, and complex IV, while other features such as ATP synthase levels were preserved (Figure 9B). Effects on proliferation were tested using tumor sphere formation assays, and showed similar reductions in growth, and further attenuation of tumor sphere growth when the inhibitors were used in combination (Figure 9, C and D). We performed a time-course, dose-response treatment on matching normal adjacent tissue and cancer cells from 3 patients and compared growth, and cancer cells were more sensitive than normal epithelial cells to ACSS2 inhibition (Supplemental Figure 5, A–D). We confirmed cancer cell–specific reduction in HIF-2α and HKII protein levels, and used transcriptomic analysis to corroborate our previous findings that ACSS2 inhibition reduced the expression of genes involved in cholesterol biosynthesis in tumors (Supplemental Figure 5, E and F). Lastly, having explored combination inhibitor strategies, we tested whether ACSS2 inhibition could be an effective means to treat ccRCCs that express HIF-2α but are resistant to PT-2385–mediated HIF-2α inhibition. We performed a dose-response, time-course ACSS2i treatment in the HIF-2α inhibitor–resistant 769-P ccRCC cell line and found that ACSS2i could block the growth of these cells when provided as a single agent (Figure 9E). Together, these results demonstrate that our in vitro and in vivo studies translate to ccRCC clinical samples, highlighting ACSS2 as a novel way to target HIF-2α and major metabolic pathways specifically in cancer cells.

## Discussion

ACSS2 has established functions in the dynamic interplay of stress responses, including interaction with HIF-2α in the setting of adaptive responses to oxygen- and nutrient-limited states (10, 22, 36–38). In ccRCC, a pseudohypoxic state is driven by constitutive HIF-2α signaling that is unimpeded by normal stress signals. ccRCC cancer cells show dependence on this signal for survival (39). Notably, even the presence or absence of p53 has little impact on the dependence of these cells on the HIF signal (40). Recent work has confirmed this model through the development of a HIF-2α–specific transcriptional inhibitor for treatment of *VHL*-associated RCCs, which has gained FDA approval (8). However, responses are incomplete, and resistance can develop over time (8, 41, 42). Parallel strategies to block HIF-2α expression or activity, such as targeting alternate mechanisms of HIF-2α protein stability, will be necessary for adequate disease control. To date, no other effective strategies to impair this critical signaling pathway have been uncovered with immediate relevance to human testing. ACSS2 is expressed in ccRCC cells and patient tissue and, our data show, plays a role to regulate HIF-2α. Finding that ccRCC cells were sensitive to inhibition or knockdown of ACSS2, we sought to directly interrogate the interaction of ACSS2-mediated acetate metabolism to maintain the stability of HIF-2α in the absence of *VHL* (3, 4, 43, 44).

Using multiple strategies to alter ACSS2 activity, we found that ccRCC cells show dependence on ACSS2 to support HIF-2α gene expression, protein stability, and downstream signaling. ccRCC cells displayed vulnerability to ACSS2 inhibition in a variety of assays of proliferation and cancer cell growth. Acetate is a major precursor to produce acetyl-CoA, particularly in times of metabolic stress. While citrate-derived acetyl-CoA contributes significantly to lipid synthesis (45, 46), acetyl-CoA produced from ACSS2 can play a key role in posttranslational and epigenetic regulation (22, 24, 47). Based on these broad roles, pharmacological or genetic inhibition of this enzyme may have a network of effects on the cell. Indeed, we found that ACSS2i reduced chromosome accessibility, and this may have contributed to reduced HIF-2α gene expression and its downstream signaling. Future studies will be aimed at dissecting the role of ACSS2 in epigenetic maintenance in ccRCC.

*VHL* is mutated in over 70% of ccRCCs, and aberrant HIF-2α signaling persists due to a lack of an effective degradation mechanism. Intriguingly, in *VHL*-deficient models we still observed proteasome-dependent degradation of HIF-2α, in addition to transcriptional downregulation. Previously, pVHL was considered to have exclusivity in directing HIF-2α proteasomal degradation; however, our data suggest the existence of an alternative mechanism. The identification and characterization of alternative HIF-2α degradation pathways could manifest into clinically relevant findings. Recently, the mitochondria-associated E3 ligase MUL1 has been shown to indirectly regulate the degradation of HIF-1α through the action of UBXN7, and was also found to promote autophagy and be suppressed in ccRCC cells (9, 48). We showed that HIF-2α expression was inversely responsive to changes in MUL1 protein expression. Further, we showed that MUL1 associates with HIF-2α in multiple ccRCC models, including patient-derived cancer cells. Future experiments will be aimed at solidifying MUL1 as an integral mechanistic link for ACSS2 to regulate HIF-2α in a tumor lacking *VHL* (Figure 9E). Beyond direct polyubiquitin- and SUMOylation-mediated degradation, MUL1 can contribute to the degradation of targets via mitophagy (10, 35). Interestingly, ACSS2 has also been linked to autophagy by its promotion of lysosomal biogenesis via acetylation (47). Thus, it is intriguing to consider the involvement of the HIF-2α posttranslational modification landscape or the role of mitophagy in the ACSS2-dependent degradation of HIF-2α.

HIF-2α is central to facilitating the metabolic reprogramming that defines the clear cell phenotype, and thus may present a unique vulnerability in these cells. Consequential to the distinct regulation of HIF-2α mediated by ACSS2, we also observed a dependence on ACSS2 to functionally sustain cholesterol biosynthesis and glucose uptake, which are classic HIF-2α–regulated metabolic programs. Further compounding its authority over ccRCC metabolism, gene set enrichment analysis identified OXPHOS as the most enriched module in ACSS2-expressing cancer cells. Supporting this analysis, we were able to demonstrate that protein expression of the OXPHOS complexes was regulated by ACSS2 activity. Apart from their role in the electron transport chain and ATP synthesis, the OXPHOS complexes are also structural components of the mitochondria and influence crista folding. Considering this along with our data, we performed electron microscopy and observed small, spherical mitochondria and crista deformation associated with ACSS2 inhibition. Intriguingly, silencing HIF2A in cells overexpressing ACSS2 resulted in a similar phenotype, suggesting that HIF-2α has a central role in regulation of mitochondrial homeostasis in ccRCC cells. Collectively, these data demonstrate the requirement for ACSS2 in supporting the metabolic environment of ccRCC.

Taken together, our data suggest that treatment with an ACSS2 inhibitor could be effective in ccRCC patients who have a *VHL* mutation. Novel small molecules targeting ACSS2 are in development and are being characterized in preclinical models of cancer (49). Recently, the first-in-human clinical trial for one of these inhibitors, MTB-9655, started treating patients with advanced solid tumors, not including ccRCC, and has reported minimal toxicity during dose escalation (50). Indeed, while HIF-2α inhibition with belzutifan has been revolutionary for pVHL syndrome, the effect is slow. We have demonstrated in clinically relevant models that ACSS2 inhibition is well tolerated and offers a more potent blockade, targeting hypoxia signaling and metabolism in a way that proves to be especially lethal to cells that are addicted to HIF-2α signaling. To our knowledge, this is the first study in ccRCC to highlight ACSS2 as an integral mediator of HIF-2α, both transcriptionally and by pVHL-independent protein degradation. These novel methods of regulating HIF-2α could provide a much-needed approach to complement and enhance the efficacy of treatment in populations resistant to HIF-2α inhibition.

## Methods

### Sex as a biological variable.

Our study exclusively examined male mice, because ccRCC is more common in males. It is unknown whether the findings are relevant to female mice.

### Materials.

Pharmacological agents used in this study were acquired commercially from Selleckchem (ACSS2 inhibitor, catalog S8588; PT-2385, catalog S8352). Mice used in this study were acquired commercially from The Jackson Laboratory. Stable cell lines carrying targeted shRNA or with different constructs are available through establishment of a material transfer agreement between Vanderbilt University Medical Center (VUMC) and the requesting institution. Optima-grade liquid chromatography–mass spectrometry (LC-MS) solvents and chemicals (ammonium bicarbonate and ammonium formate) for the LC-MS analyses were obtained from Thermo Fisher Scientific.

### Cell cultures.

HKC cells were a gift from L.C. Racusen (Johns Hopkins University, Baltimore, Maryland, USA) (51). HEK293FT, A498, 786-O, and 769-P cell lines were obtained from ATCC. 786-O Cas9 cells were purchased from Genecopoeia. Before experimentation, all cell lines were tested for mycoplasma contamination using the ATCC Universal Mycoplasma detection kit (catalog 30-1012K). HEK293FT, HKC, and A498 cell lines were cultured using Gibco DMEM supplemented with 10% FBS, 1% l-glutamine, and 10 mL/L penicillin/streptomycin (MilliporeSigma). 768-O and 769-P cell lines were cultured using Gibco RPMI 1640 supplemented with 10% FBS, 1% l-glutamine (MilliporeSigma), and 10 mL/L penicillin/streptomycin (MilliporeSigma). All cells were maintained at 37°C in a 5% CO_2_ incubator.

### Animal studies.

Six-week-old male NOD scid IL2Rg^null^ (NSG) mice were purchased from The Jackson Laboratory (catalog 005557) and used in all tumorigenesis xenograft models. Mice were housed 5 to a cage in a temperature- and humidity-controlled space (20°C–25°C, 45%–64% humidity) with regulated water and lighting (12 hours light/12 hours dark) within the animal facility. Before inoculation, 786-O cells were cultured as described above, trypsinized, counted, and resuspended in growth medium and Matrigel at a 1:1 ratio. For all in vivo experiments, NSG mice were subcutaneously injected with 100 μL of the cell suspension providing 5 × 10^6^ cells. Mice in pharmacological studies were subjected to daily treatment with vehicle control or 15 mg/kg ACSS2i delivered via intraperitoneal injection. Alternatively, mice that were inoculated with 786-O cells transduced to express the pTRIPZ doxycycline-inducible shRNA system were given a 200 mg/kg doxycycline rodent diet (Bio-Serv) and allowed to feed ad libitum. Tumor burden was monitored via weekly manual, digital caliper measurement with intermittent measurements taken as needed. Mice were euthanized once the first tumor reached size endpoint (1,000 mm^3^) or if discomfort was observed as outlined in the IACUC-approved protocol.

### Patient samples.

Primary cell cultures were derived from tumors of patients diagnosed with clear cell renal cell carcinoma being treated at VUMC. Single-cell suspensions were generated from the tumors and were subsequently cultured in Gibco RPMI 1640 medium supplemented with 10% FBS, 1% l-glutamine (MilliporeSigma), B-27 supplement (Gibco; 10 mL, 50× stock), and 1× Antibiotic-Antimycotic (Gibco). Fresh growth medium was provided every other day for approximately 10 days until a monolayer of epithelial cells was established, at which point the primary cells were able to be passaged.

### Protein extraction and Western blotting.

Cells were harvested, suspended in RIPA buffer supplemented with 1× Halt protease inhibitor cocktail (Thermo Fisher Scientific), and subjected to mechanical needle lysing with a 25-gauge needle (BD Biosciences) while being kept on ice. Cell lysates were pelleted via centrifugation at 14,000*g* for 5 minutes at 4°C, and supernatants were transferred to fresh Eppendorf tubes for immediate analysis via SDS-PAGE or preservation at –20°C. Protein concentrations were determined using the BCA protein quantification method. For SDS-PAGE analysis, 50 μg of protein per sample was loaded into and run on a 4%–20% gradient polyacrylamide gel (Bio-Rad), followed by transfer onto a PVDF membrane. The membranes were subjected to immunoblotting with the primary antibodies found in Supplemental Table 1. For Western blot analysis, β-actin served as the loading control. In instances of parallel gel use for figure assembly, individual loading controls for the corresponding gels are presented. All blots presented in figures are representative of results obtained from experiments performed in biological triplicate with each target being analyzed at least twice.

### Immunoprecipitation.

Cell lysates were prepared as described above, and 500 μg of total protein per sample was first cleared via incubation with protein A–agarose beads at 4°C for 1 hour, then spun down at 3,500*g* for 10 minutes at 4°C. The precleared lysates were transferred to fresh Eppendorf tubes and incubated with the primary antibody overnight at 4°C. The following day, 30 μL of a 50% protein A–agarose bead slurry was added to the immunoprecipitation reactions and continued incubating for 2 hours at 4°C on a rotator. The immunoprecipitations were then microcentrifuged at 3,500*g* for 10 minutes at 4°C to collect the beads, which were washed 5 times with RIPA buffer supplemented with protease inhibitors. After the final wash, the pelleted beads were resuspended with 3× SDS sample buffer, vortexed briefly, and microcentrifuged at 14,000*g* for 1 minute at room temperature. Samples were heated at 95°C for 5 minutes, microcentrifuged at 14,000*g* for 1 minute at room temperature, and loaded into a gel for SDS-PAGE to be analyzed via Western blotting.

### Lentiviral transduction.

HEK293FT cells were allowed to reach 70% confluence, and on the day of transfection, 1 hour before transfection growth medium was replaced with Opti-MEM (Gibco). For each plate, a transfection cocktail was prepared using 20 μg of experimental plasmid (Supplemental Table 2), 15 μg PAX2, 6.5 μg of pMD2.G, 62 μL of CaCl_2_, and sterile dH_2_O to bring the total volume to 500 μL. Five hundred microliters of 2× HEPES-buffered saline was added and mixed by pipetting. The transfection medium was added to the HEK293FT cells, gently swirled, and incubated for 5–7 hours at 37°C. Transfection medium was discarded and replaced with 10–12 mL of fresh growth medium, and the cells were allowed to produce viral particles for 48–72 hours. The lentiviral supernatants were collected and passed through a 0.45 μm filter to be used immediately or stored at –80°C for later use.

Lentiviral transduction of 786-O cells was performed by mixing of 1 mL lentivirus, 6.5 μL of 10 mg/mL Polybrene, and 7 mL growth medium and incubation for 24 hours at 37°C. The next day, transduction medium was replaced with fresh growth medium, and cells were returned to a 37°C incubator for another 24 hours. On the following day, the medium of the transduced cells was supplemented with the antibiotic corresponding to the selectable antibiotic resistance marker in each plasmid for 24 hours, and resistant clones were expanded for experimental use.

### ATAC-seq.

ATAC-seq library preparations, sequencing reactions, and bioinformatics analysis were conducted at Azenta Life Sciences as follows:

Live cell samples were thawed, washed, and treated with DNase I (Life Tech, catalog EN0521) to remove genomic DNA contamination. Live cell samples were quantified and assessed for viability using a Countess Automated Cell Counter (Thermo Fisher Scientific). After cell lysis and cytosol removal, nuclei were treated with Tn5 enzyme (Illumina, catalog 20034197) for 30 minutes at 37°C and purified with a Minelute PCR Purification Kit (Qiagen, catalog 28004) to produce tagmented DNA samples. Tagmented DNA was barcoded with Nextera Index Kit v2 (Illumina, catalog FC-131-2001) and amplified via PCR before an SPRI Bead (Beckman Coulter) clean-up to yield purified DNA libraries.

The sequencing libraries were clustered on a single lane of a flow cell. After clustering, the flow cell was loaded on the Illumina HiSeq instrument (4000 or equivalent) according to the manufacturer’s instructions. The samples were sequenced using a 2 × 150 bp paired-end configuration. Image analysis and base calling were conducted with HiSeq Control Software. Raw sequence data (.bcl files) generated from Illumina HiSeq were converted into FASTQ files and demultiplexed using Illumina’s bcl2fastq 2.17 software. One mismatch was allowed for index sequence identification.

After investigation of the quality of the raw data, sequencing adapters and low-quality bases were trimmed using Trimmomatic 0.38. Cleaned reads were aligned to reference genome hg38 using Bowtie 2 (https://github.com/BenLangmead/bowtie2). Aligned reads were filtered using Samtools 1.9 (https://github.com/samtools/samtools/releases/) to keep alignments that (a) had a minimum mapping quality of 30, (b) were aligned concordantly, and (c) were the primary called alignments. PCR or optical duplicates were marked using Picard 2.18.26 (https://github.com/broadinstitute/picard/releases) and removed. Before peak calling, reads mapping to mitochondria were called and filtered, and reads mapping to unplaced contigs were removed.

MACS2 2.1.2 (https://github.com/macs3-project/MACS/releases) was used for peak calling to identify open chromatin regions. Valid peaks from each group or condition were merged, and peaks called in at least 66% of samples were kept for downstream analyses. For each pairwise comparison, peaks from condition A and condition B were merged, and peaks found in either condition were kept for downstream analyses. Reads falling beneath peaks were counted in all samples, and these counts were used for differential peak analyses using the R package Diffbind.

To explore the ATAC signals across the genome, we used the python package deepTools (v3.5.0). Briefly, the BAM files from Genewiz were first transformed to bigWig files using the command “bamCoverage” with parameters “-bs 10 --normalizeUsing CPM.” Then command “computeMatrix” and “plotHeatmap” were used to generate heatmaps of ATAC signal 5 kb around the accessible regions from the bigWig files. The peaks called from different groups were merged with bedtools (https://github.com/arq5x/bedtools2) and used as the reference regions in the heatmap.

### Metabolic gene expression.

To analyze changes in expression of genes involved in metabolism, we extracted RNA as described above and performed a gene expression assay using the NanoString nCounter Metabolic Pathways panel. The assay was performed by Vanderbilt Advanced Technologies for Genomics (VANTAGE) core facility following the manufacturer’s recommended procedures. RNA concentrations were determined via Qubit (Thermo Fisher Scientific) and normalized to 20 ng/μL. For each sample hybridization reaction, we used 100 ng of total RNA, and hybridizations were performed for a total of 20 hours. Analysis of raw data was performed using nSolver Analysis Software 4.0 (NanoString). For all analyses, the background threshold parameters were set to the mean of 8 negative control spike-in genes, and mRNA expression was normalized to the geometric mean of 6 positive control spike-in genes and 20 housekeeping genes (*ABCF1*, *AGK*, *COG7*, *DHX16*, *DNAJC14*, *EDC3*, *FCF1*, *G6PD*, *MRPS5*, *NRDE2*, *OAZ1*, *POLR2A*, *SAP130*, *SDHA*, *STK11IP*, *TBC1D10B*, *TBP*, *TLK2*, *UBB*, and *USP39*).

Data were analyzed by ROSALIND (https://rosalind.bio/), with a HyperScale architecture developed by ROSALIND Inc. Read distribution percentages, violin plots, identity heatmaps, and sample multidimensional scaling plots were generated as part of the quality control step. The limma R library was used to calculate fold changes and *P* values and perform optional covariate correction. Clustering of genes for the final heatmap of differentially expressed genes was done by the partitioning around medoids (PAM) method using the fpc R library, which takes into consideration the direction and type of all signals on a pathway, the position, role, and type of every gene, etc. Hypergeometric distribution was used to analyze the enrichment of pathways, gene ontology, domain structure, and other ontologies. The topGO R library was used to determine local similarities and dependencies between Gene Ontology terms to perform Elim pruning correction. Several database sources were referenced for enrichment analysis, including InterPro, NCBI, MSigDB, Reactome, and WikiPathways.

### Crystal violet cell growth assay.

To assess the impact of the experimental conditions on cell growth, 2.5 × 10^4^ or 1 × 10^5^ cells were seeded into 12- well or 6-well plates, respectively, and allowed to adhere for 24 hours. On the following day, growth medium was supplemented with vehicle, 2 μg/mL doxycycline, or various doses of the ACSS2i, and the effect on growth was monitored over time by staining with a 1% crystal violet solution prepared in 20% methanol and 80% dH_2_O. After imaging, the crystal violet stain was stripped using a 1% deoxycholate solution, which was transferred to a 96-well plate and read on a Promega GloMax plate reader at 600 nm to quantify the crystal violet staining.

### BrdU cell proliferation assay.

To determine the impact of experimental conditions on proliferation, we used a BrdU cell proliferation ELISA kit (Abcam, catalog ab126556) following the manufacturer’s suggested protocol. Briefly, cells were seeded at a density of 1 × 10^4^ cells per well in normal growth medium. Additionally, a duplicate set of cells like those in the experiment were seeded and did not receive BrdU labeling reagent, while several wells were also left empty of cells to thoroughly account for background. For 24-hour measurements, growth medium was supplemented with ACSS2i or doxycycline, where applicable, and allowed to incubate for 22 hours at 37°C. BrdU labeling solution was added for the final 2 hours of the 24-hour incubation before continuation of the ELISA protocol. For 48-hour measurements, growth medium was supplemented with ACSS2i and incubated for 24 hours at 37°C before addition of the BrdU labeling solution for the final 24 hours of the 48-hour incubation. After BrdU labeling, cells were fixed for 30 minutes with 1× fixing solution, washed 3 times with 1× wash buffer, and incubated with detector antibody for 1 hour at room temperature. After incubation with detector antibody, cells were washed 3 times with 1× wash buffer followed by addition of 1× peroxidase–goat anti-mouse IgG conjugate for 30 minutes at room temperature. The ELISA reactions were washed 3 more times with 1× wash buffer and a final time with distilled water. After plates were patted dry, TMB peroxidase substrate was added and reactions incubated for 30 minutes at room temperature in the dark. The reactions were stopped via addition of stop solution, and BrdU incorporation was quantified by reading of plates at 450 nm on a GloMax plate reader.

### Tumor sphere formation assay.

786-O cells were seeded onto 12-well ultra-low-attachment plates (Corning) at a density of 1 × 10^3^ cells per well. Cells were subjected to treatment immediately after plating, and fresh growth medium with vehicle or the ACSS2i was provided every other day. Sphere formation was monitored via microscope daily, and after 7 days, images were captured at ×4 and ×10 magnification on a Keyence BZ-X800 microscope.

### Anchorage-independent growth assay.

Tumorigenic potential was evaluated using an anchorage-independent growth assay. Briefly, a 2.5% agarose solution was prepared using low-melt agarose and autoclaved dH_2_O. This solution was diluted 1:4 in growth medium and used to coat 6-well plates, which were returned to the 37°C incubator and allowed to set, while cells were trypsinized and counted. For each condition, 1 × 10^5^ cells were resuspended in 9 mL of growth medium and 1 mL of a 3% agarose solution (0.3% final concentration), and 1 mL (1 × 10^4^ cells) of the cell solution was added per well. The top layer was allowed to set for an hour at 37°C, at which point 2 mL growth medium was added to each well. Growth medium and treatments, where applicable, were exchanged every other day, and colony formation was monitored via microscope. Images of colonies were captured using an EVOS imaging system (Thermo Fisher Scientific).

### Tissue microarray immunofluorescence staining and imaging.

Human ccRCC tissue microarray (TMA) was provided by Scott Haake (IRB 10565510-19) and the Translational Pathology Shared Research Core at VUMC (National Cancer Institute Cancer Support Grant P30CA068485). Paraffin-embedded TMA slides were prepared for immunofluorescence and stained with anti-ACSS2 (rabbit; Abcam, 133664; 1:250), anti-HIF2A (rabbit; Novus, NB100-122; 1:250), and anti-AE1/AE3 (mouse; Abcam, 27988; 1:500) as previously described (52). Briefly, slides were deparaffinized in xylene and rehydrated in serial ethanol dilutions. Antigen retrieval was performed by heating of slides for 17 minutes in Tris-EDTA buffer, pH 9, in a pressure cooker at 110°C. Slides were cooled to room temperature and blocked with 2.5% horse serum (Vector Laboratories). After blocking, slides were incubated overnight at 4°C with primary antibody in horse serum. Slides were incubated in anti-rabbit or -mouse HRP-conjugated secondary antibody (Vector Laboratories) for 1 hour at room temperature the following day and subsequently incubated in 1:500 Opal 520, Opal 570, or Opal 590 (Akoya) for 10 minutes. For serial staining, slides were stripped using citric acid buffer, pH 6.1, in a pressure cooker at 110°C for 2 minutes, and staining was repeated using different antibody and Opal fluorophore. After the last Opal staining, slides were mounted using antifade gold mount with DAPI (Invitrogen). Stained images were acquired using an Aperio Versa 200 Automated Slide imaging system (Leica/Aperio) via the VUMC Digital Histology Shared Resource core and analyzed with Fiji software (https://fiji.sc/). Quantification of markers was done by measurement of total amount of fluorescence divided by total area of tissue (H&E). Representative ×63 images were taken using a Zeiss LSM 880 confocal microscope at the Vanderbilt Cell Imaging Shared Research Core.

### Immunohistochemistry staining and imaging.

Tumor specimens were harvested immediately after animal euthanasia. Each tumor was washed with cold PBS and fixed overnight with 10% neutral-buffered formalin. The tumors were paraffin-embedded, sectioned, and stained by the Translational Pathology Shared Resource facility at VUMC using standard immunohistochemistry procedures. Images of stained tumor sections were obtained using an Olympus light microscope.

### HIF-2α protein stability assay.

To assess the impact of ACSS2 inhibition on HIF-2α protein stability, 786-O cells were treated with 1 μM of MG132 for 1 hour. After initial incubation with MG132, growth medium was exchanged and supplemented with DMSO or the ACSS2i for 24 hours. Upon completion of treatments, cell lysates were prepared as described above and analyzed via SDS-PAGE and Western blotting. HIF-2α expression was quantified using ImageJ and normalized to β-actin.

### Gas chromatography–mass spectrometry for cholesterol quantification.

Lipids were extracted using a previously described method (53). Briefly, extracts were filtered, and lipids recovered in the chloroform phase. Individual lipid classes were separated by thin-layer chromatography using Silica Gel 60 Å plates developed in petroleum ether, ethyl ether, acetic acid (80:20:1) and visualized by rhodamine 6G. Phospholipids, diglycerides, triglycerides, and cholesteryl esters were scraped from the plates and methylated using BF3/methanol as described previously (54). The methylated fatty acids were extracted and analyzed by gas chromatography. Gas chromatographic analyses were carried out on an Agilent 7890A gas chromatograph equipped with flame ionization detectors and a capillary column (SP2380, 0.25 mm × 30 m, 0.25 μm film, Supelco). Helium was used as a carrier gas. The oven temperature was programmed from 160°C to 230°C at 4°C/min. For total cholesterol, internal standard (5-a-cholestane) was added to a portion of the lipid extract and saponified at 80°C in 1N KOH in 90% methanol for 1 hour. For unesterified cholesterol, internal standard was added to a portion of the lipid extract, concentrated under nitrogen, and solubilized in hexane to inject onto the gas chromatograph. The Agilent 7890A gas chromatograph was equipped with an HP-50+ column (0.25 mm inner diameter × 30 m, Agilent) and a flame ionization detector. The oven temperature was programmed from 260°C to 280°C, and helium was used as the carrier gas (55).

### Acetate quantitation assay.

To monitor ACSS2 activity, acetate concentrations in the growth medium were measured over time by an acetate quantitation colorimetric assay (BioVision, catalog K658) using the manufacturer’s suggested protocol. Briefly, cells were seeded at a density of 2.5 × 10^5^ cells per well in a 6-well plate. The following day, fresh growth medium was added to the cells, as well as a blank well, supplemented with ACSS2i or doxycycline, where applicable, and incubated for 24 hours at 37°C. Growth medium was collected after the 24-hour incubation and immediately processed for analysis with the colorimetric assay. For each sample, 25 μL of medium was combined with 25 μL of assay buffer and 50 μL of the reaction buffer using the manufacturer-provided reagents and guidelines. Following a 40-minute incubation at room temperature, acetate concentration measurements were obtained via colorimetric detection at OD_450nm_ using a GloMax plate reader (Promega).

### Transmission electron microscopy.

All electron microscopy reagents were purchased from Electron Microscopy Sciences. Cell cultures were grown to approximately 80% confluence, subjected to treatment conditions, and fixed with 2.5% glutaraldehyde in 0.1 M cacodylate for 1 hour at room temperature followed by 24 hours at 4°C. After fixation the cells were mechanically lifted from the tissue culture plates and pelleted, then sequentially postfixed with 1% tannic acid, 1% OsO_4_, and stained en bloc with 1% uranyl acetate. Samples were dehydrated in a graded ethanol series, infiltrated with Quetol 651–based Spurr’s resin (Electron Microscopy Sciences), and polymerized at 60°C for 48 hours. Ultrathin sections were prepared on a UC7 ultramicrotome (Leica) with a nominal thickness of 70 nm and collected onto 300-mesh nickel grids. Sections were stained with 2% uranyl acetate and lead citrate.

Samples were imaged using a Tecnai T12 operating at 100 kV equipped with an AMT NanoSprint CMOS camera using AMT imaging software. Analysis of transmission electron microscopy data was performed in Fiji.

### Mitochondrial biogenesis assay.

The synthesis of mitochondria was assessed using a Mitochondrial Biogenesis In-Cell ELISA colorimetric kit (Abcam, ab110217), which measures the abundance of subunit I of complex IV (mitochondrial DNA–encoded) and the 70 kDa subunit of complex II (nuclear-encoded). For each experiment, the manufacturer’s suggested procedures were followed. Briefly, 1.5 × 10^4^ cells were seeded into the wells of a 96-well plate and subjected to treatments 24 hours later. Upon completion of the experimental conditions, cells were fixed for 20 minutes with 4% paraformaldehyde and then washed 3 times with PBS, and the plates were blotted dry. Subsequently, 100 μL of 0.5% acetic acid was added to each well for 5 minutes to block endogenous alkaline phosphatase activity. The wells were washed once again with PBS followed by addition of 1× permeabilization buffer for 30 minutes. After permeabilization, cells were incubated with 1× blocking solution for 2 hours followed by overnight incubation with primary antibody at 4°C. After incubation with primary antibody, cells were washed 3 times with PBS and incubated with secondary antibody for 1 hour at room temperature. Secondary antibody was removed, and cells were washed 4 times with 1× wash buffer. Expression of the 70 kDa subunit of complex II (SDH-A) and subunit I of complex IV (COX-I) was detected and quantified by wavelength detection at 405 nm and 600 nm, respectively, using a GloMax plate reader.

### Statistics.

Details regarding the statistical analysis of experiments throughout the study can be found in the figure legends. All statistical analyses were performed using GraphPad Prism 6 following the suggested test parameters. In the determination of statistical significance, a minimum of 3 independent experiments were performed, and a *P* value of less than 0.05 was set as the threshold. All data are represented as mean ± SD. For all box-and-whisker plots, the data are graphed to display all data points (min-to-max) where the whiskers go down to the smallest value and up to the largest. Per the GraphPad Prism website, the bounds of the boxes always extend from the 25th to 75th percentiles. The line intersecting each box is representative of the median.

### Study approval.

All in vivo mouse studies were designed and performed in accordance with animal protocols approved by the Institutional Animal Care and Use Committee of VUMC. For ex vivo studies with ccRCC patient cells, primary cell cultures were derived from tumors of patients diagnosed with clear cell renal cell carcinoma being treated at VUMC. Informed written consent was collected from all patients whose samples were used in this study, and all samples were processed and utilized in accordance with the IRB protocol (151549).

### Data availability.

The ATAC-seq data set has been deposited in the Gene Expression Omnibus repository (45) and made publicly accessible (GSE204947). The unedited blots are provided as an individual file that is part of the supplemental material. Data used for graphing/quantification purposes are provided in the Supporting Data Values file. All raw data used for quantification and statistical analysis are available in a single GraphPad Prism file that can be made available upon request.

## Author contributions

ZAB performed most of the experimental work, data analysis, and statistical analysis. ZAB, JCR, and WKR participated in study conception and design, data interpretation, and drafting of the manuscript. EKA and WAB assisted with experimental work. ENA performed immunofluorescence staining and data analysis of TMAs. LMV performed analysis of the scRNA-Seq data set. ESK was responsible for obtaining electron microscopy images. MMW, RAH, and ML processed patient samples and prepared primary cell cultures for experimental work. XY performed bioinformatics analysis on ATAC-seq data. KEB provided access to patient samples. All coauthors assisted with reviewing and editing the manuscript.

## Supplementary Material

Supplemental data

Unedited blot and gel images

Supporting data values

## Version 1 06/17/2024

Electronic publication

## References

[1] Cancer Genome Atlas Research Network (2013). Comprehensive molecular characterization of clear cell renal cell carcinoma. Nature.

[2] Toma MI (2008). Loss of heterozygosity and copy number abnormality in clear cell renal cell carcinoma discovered by high-density Affymetrix 10K single nucleotide polymorphism mapping array. Neoplasia.

[3] Kondo K (2002). Inhibition of HIF is necessary for tumor suppression by the von Hippel-Lindau protein. Cancer Cell.

[4] Shen C (2011). Genetic and functional studies implicate HIF1α as a 14q kidney cancer suppressor gene. Cancer Discov.

[5] Du W (2017). HIF drives lipid deposition and cancer in ccRCC via repression of fatty acid metabolism. Nat Commun.

[6] Qiu B (2015). HIF2α-dependent lipid storage promotes endoplasmic reticulum homeostasis in clear-cell renal cell carcinoma. Cancer Discov.

[7] Wallace EM (2016). A small-molecule antagonist of HIF2α is efficacious in preclinical models of renal cell carcinoma. Cancer Res.

[8] Jonasch E (2021). Belzutifan for renal cell carcinoma in von Hippel-Lindau disease. N Engl J Med.

[9] Cilenti L (2020). Mitochondrial MUL1 E3 ubiquitin ligase regulates Hypoxia Inducible Factor (HIF-1α) and metabolic reprogramming by modulating the UBXN7 cofactor protein. Sci Rep.

[10] Li J (2015). Mitochondrial outer-membrane E3 ligase MUL1 ubiquitinates ULK1 and regulates selenite-induced mitophagy. Autophagy.

[11] Courtney KD (2018). Isotope tracing of human clear cell renal cell carcinomas demonstrates suppressed glucose oxidation in vivo. Cell Metab.

[12] Brooks SA (2016). Alternate metabolic programs define regional variation of relevant biological features in renal cell carcinoma progression. Clin Cancer Res.

[13] Liu Y (2016). The place of FDG PET/CT in renal cell carcinoma: value and limitations. Front Oncol.

[14] Reinfeld BI (2021). Cell-programmed nutrient partitioning in the tumour microenvironment. Nature.

[15] Kaushik AK (2022). In vivo characterization of glutamine metabolism identifies therapeutic targets in clear cell renal cell carcinoma. Sci Adv.

[16] Oyama N (2014). Diagnosis of complex renal cystic masses and solid renal lesions using PET imaging: comparison of 11C-acetate and 18F-FDG PET imaging. Clin Nucl Med.

[17] Ho CL (2012). Dual-tracer PET/CT in renal angiomyolipoma and subtypes of renal cell carcinoma. Clin Nucl Med.

[18] Kamphorst JJ (2014). Quantitative analysis of acetyl-CoA production in hypoxic cancer cells reveals substantial contribution from acetate. Cancer Metab.

[19] Tan SK (2023). Fatty acid metabolism reprogramming in ccRCC: mechanisms and potential targets. Nat Rev Urol.

[20] Zhang S (2018). Acetyl-CoA synthetase 2 enhances tumorigenesis and is indicative of a poor prognosis for patients with renal cell carcinoma. Urol Oncol.

[21] Schug ZT (2015). Acetyl-CoA synthetase 2 promotes acetate utilization and maintains cancer cell growth under metabolic stress. Cancer Cell.

[22] Chen R (2017). Coordinate regulation of stress signaling and epigenetic events by Acss2 and HIF-2 in cancer cells. PLoS One.

[23] Chen R (2015). The acetate/ACSS2 switch regulates HIF-2 stress signaling in the tumor cell microenvironment. PLoS One.

[24] Nagati JS (2021). Mammalian acetate-dependent acetyl CoA synthetase 2 contains multiple protein destabilization and masking elements. J Biol Chem.

[25] Clark DJ (2019). Integrated proteogenomic characterization of clear cell renal cell carcinoma. Cell.

[26] Linehan WM, Ricketts CJ (2019). The Cancer Genome Atlas of renal cell carcinoma: findings and clinical implications. Nat Rev Urol.

[27] Ricketts CJ (2018). The Cancer Genome Atlas comprehensive molecular characterization of renal cell carcinoma. Cell Rep.

[28] Bi K (2021). Tumor and immune reprogramming during immunotherapy in advanced renal cell carcinoma. Cancer Cell.

[29] Covello KL (2006). HIF-2α regulates Oct-4: effects of hypoxia on stem cell function, embryonic development, and tumor growth. Genes Dev.

[30] Hu CJ (2006). Differential regulation of the transcriptional activities of hypoxia-inducible factor 1 alpha (HIF-1α) and HIF-2α in stem cells. Mol Cell Biol.

[31] Li X (2017). Nucleus-translocated ACSS2 promotes gene transcription for lysosomal biogenesis and autophagy. Mol Cell.

[32] Palikaras K (2015). Balancing mitochondrial biogenesis and mitophagy to maintain energy metabolism homeostasis. Cell Death Differ.

[33] Shi G, McQuibban GA (2017). The mitochondrial rhomboid protease PARL is regulated by PDK2 to integrate mitochondrial quality control and metabolism. Cell Rep.

[34] Tang FL (2015). VPS35 deficiency or mutation causes dopaminergic neuronal loss by impairing mitochondrial fusion and function. Cell Rep.

[35] Yun J (2014). MUL1 acts in parallel to the PINK1/parkin pathway in regulating mitofusin and compensates for loss of PINK1/parkin. Elife.

[36] Comerford SA (2014). Acetate dependence of tumors. Cell.

[37] Mashimo T (2014). Acetate is a bioenergetic substrate for human glioblastoma and brain metastases. Cell.

[38] Xu M (2014). An acetate switch regulates stress erythropoiesis. Nat Med.

[39] Kaelin WG (2022). Von Hippel–Lindau disease: insights into oxygen sensing, protein degradation, and cancer. J Clin Invest.

[40] Stransky LA (2022). Sensitivity of VHL mutant kidney cancers to HIF2 inhibitors does not require an intact p53 pathway. Proc Natl Acad Sci U S A.

[41] Cho H, Kaelin WG (2016). Targeting HIF2 in clear cell renal cell carcinoma. Cold Spring Harb Symp Quant Biol.

[42] Choueiri TK (2021). Inhibition of hypoxia-inducible factor-2α in renal cell carcinoma with belzutifan: a phase 1 trial and biomarker analysis. Nat Med.

[43] Kondo K (2003). Inhibition of HIF2α is sufficient to suppress pVHL-defective tumor growth. PLoS Biol.

[44] Gordan JD (2008). HIF-α effects on c-Myc distinguish two subtypes of sporadic VHL-deficient clear cell renal carcinoma. Cancer Cell.

[45] Wellen KE (2009). ATP-citrate lyase links cellular metabolism to histone acetylation. Science.

[46] Zhao S (2016). ATP-citrate lyase controls a glucose-to-acetate metabolic switch. Cell Rep.

[47] Bulusu V (2017). Acetate recapturing by nuclear acetyl-CoA synthetase 2 prevents loss of histone acetylation during oxygen and serum limitation. Cell Rep.

[48] Yuan Y (2019). Mitochondrial E3 ubiquitin ligase 1 promotes autophagy flux to suppress the development of clear cell renal cell carcinomas. Cancer Sci.

[49] Miller KD (2021). Targeting ACSS2 with a transition-state mimetic inhibits triple-negative breast cancer growth. Cancer Res.

[50] Perets R (2022). Phase 1 first-in-human trial of MTB-9655, the first oral inhibitor of ACSS2, in patients with advanced solid tumors. J Clin Oncol.

[51] Racusen LC (1997). Cell lines with extended in vitro growth potential from human renal proximal tubule: characterization, response to inducers, and comparison with established cell lines. J Lab Clin Med.

[52] Sorrelle N (2019). Improved multiplex immunohistochemistry for immune microenvironment evaluation of mouse formalin-fixed, paraffin-embedded tissues. J Immunol.

[53] Folch J (1957). A simple method for the isolation and purification of total lipids from animal tissues. J Biol Chem.

[54] Morrison WR, Smith LM (1964). Preparation of fatty acid methyl esters and dimethylacetals from lipids with boron fluoride–methanol. J Lipid Res.

[55] Rudel LL (1998). Dietary monounsaturated fatty acids promote aortic atherosclerosis in LDL receptor–null, human apoB100–overexpressing transgenic mice. Arterioscler Thromb Vasc Biol.

