# Supplementary material for: HIF-2α expression and metabolic signaling require ACSS2 in clear cell renal cell carcinoma
Source: J Clin Invest. 2024 Jun 17;134(12):e164249. doi: 10.1172/JCI164249 (PMC11178540; doi:10.1172/JCI164249)
Supplement: Supplemental data [file jci-134-164249-s027.pdf]

SUPPLEMENTAL TABLES AND FIGURES

Table S1. List of primary antibodies used throughout the study.

Antibody	Application	Concentration	Source	Catalog Number
ACSS2	WB	1:1,000	Cell Signaling	3658S
B-Actin	WB	1:5,000	Abcam	ab8227
EGLN3	WB	1:1,000	Novus	NB100-139
EPAS1	WB, IP	1:500, 1:50	Santa Cruz	sc-46691
Epo	WB	1:1,000	Santa Cruz	sc-5290
GLUT1	WB	1:1,000	Abcam	ab115730
Hexokinase II	WB	1:1,000	Cell Signaling	2867S
HIF-2α	WB, IP	1:500, 1:100	Novus	NB100-122
K48 polyubiquitin	WB	1:1,000	Cell Signaling	8081S
LC3 A/B	WB	1:1,000	Cell Signaling	12741S
MUL1	WB, IP	1:500; 1:100	Abcam	ab84067
SREBP1	WB	1:1,000	Novus	NB600-582
SREBP2	WB	1:1,000	Novus	NBP1-54446
VEGFR2	WB	1:500	Cell Signaling	9698S

Table S2. List of plasmid constructs used throughout the study.

Plasmid	Clone	Source	Catalog Number
TRIPZ Human Non-silencing shRNA	N/A	Horizon Discovery	RHS4743
TRIPZ Human ACSS2 shRNA	V3THS_366720	Horizon Discovery	RHS4696-200764456
TRIPZ Human ACSS2 shRNA	V3THS_366722	Horizon Discovery	RHS4696-200767025
pLX304 CCSB-Broad LentiORF	ACSS2	Horizon Discovery	OHS6085-213584639
pLX304 CCSB-Broad LentiORF	MUL1	Horizon Discovery	OHS5898-202617308
pLX304 empty vector	N/A	Addgene	25890
GIPZ EPAS1 shRNA	V2LHS_113750,113752, 318637, 402392, 318640	Horizon Discovery	RHS4531-EG2034
pLKO MUL1 shRNA	TRCN0000033937, TRCN0000236073	Sigma	N/A
pLV[Exp]-Bsd-CMV+intron>{W	NP_001070020.2	VectorBuilder	VB240130-1368ddq

T-ACSS2human}/mCherry			
pLV[Exp]-Bsd-CMV+intron>{T376K}/T2A/mCherry	NP_001070020.2 with T376K substitution	VectorBuilder	VB240130-1394xvq

Figure S1

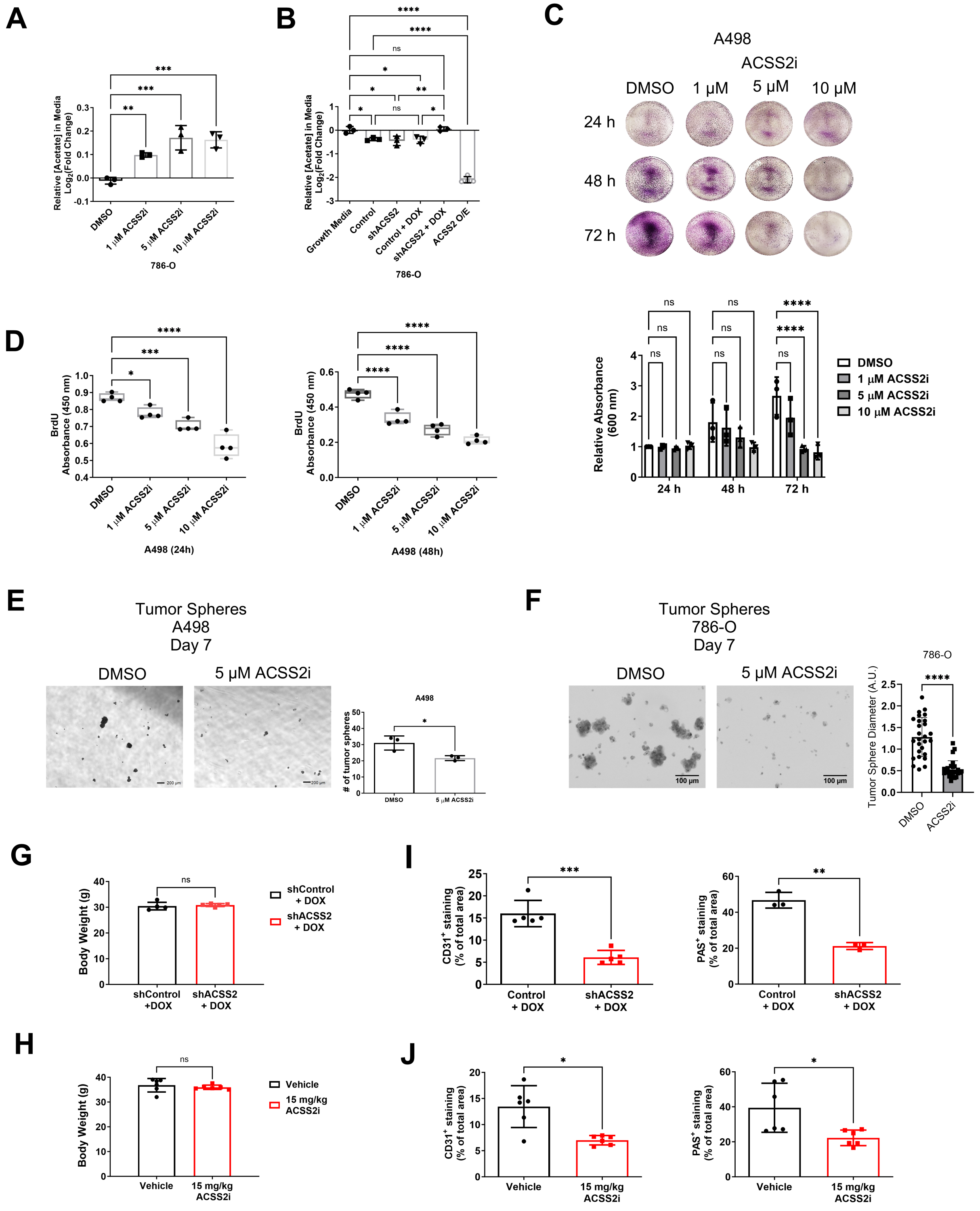
A
B
C
A498
ACSS2i
DMSO
1 μM
5 μM
10 μM
24 h
48 h
72 h
786-O
D
A498 (24h)
A498 (48h)
DMSO
1 μM ACSS2i
5 μM ACSS2i
10 μM ACSS2i
Relative Absorbance (600 nm)
E
Tumor Spheres
A498
Day 7
DMSO
5 μM ACSS2i
F
Tumor Spheres
786-O
Day 7
Tumor Sphere Diameter (A.U.)
G
H
I
J
Body Weight (g)
shControl + DOX
shACSS2 + DOX
Vehicle
15 mg/kg ACSS2i
CD31+ staining (% of total area)
PAS+ staining (% of total area)
Control + DOX

Figure S1. Effects of ACSS2 inhibition on ccRCC growth, Related to Figures 2 and 3. A) Bar graph showing relative concentrations of acetate in growth media of 786-O cells treated with DMSO, 1 μM, 5 μM, or 10 μM ACSS2 inhibitor for 24 hours. Data are presented as Log_2 values of fold change. Statistical significance determined using an ordinary one-way ANOVA and Bonferroni's multiple comparisons test (**, P-value < 0.01; ***, P-value < 0.001). **B)** Bar graph showing relative concentrations of acetate in growth media of 786-O cells transduced to express V5-ACSS2 overexpression vector, or the DOX-inducible shControl or shACSS2 constructs. Data are presented as Log_2 values of fold change. Statistical significance determined using an ordinary one-way ANOVA and Tukey's multiple comparisons test (*, P-value < 0.05; **, P-value < 0.01; ****, P-value < 0.0001). Data are represented as mean +/- SD. **C)** Representative images of crystal violet staining from a dose-response time-course treatment with DMSO or ACSS2 inhibitor in A498 cells. Quantification of three-independent replicates are provided in the bar graph. Statistical significance was determined using Tukey's multiple comparisons test. **D)** Box and whisker plots showing the absorbance values detected at OD450 nm of BrdU ELISA assays performed on A498 cells treated for 24 hours (left) or 48 hours (right) with DMSO, 1 μM, 5 μM, or 10 μM ACSS2 inhibitor (n=4). Statistical significance was determined using Bonferroni's multiple comparisons test (*, P < 0.05; ***, P < 0.001; ****, P < 0.0001). **E)** Representative images of A498 tumor spheres at day 7 of growth in ultra-low attachment plates treated with either DMSO or 5 μM ACSS2i. Quantification of number of spheres from three-independent replicates are provided in the bar graph. Statistical significance was determined using an unpaired, two-tailed t-test (*, P < 0.05). **F)** Representative images of 786-O tumor spheres at day

7 of growth in ultra-low attachment plates treated with either DMSO (n=3) or 5 μM ACSS2i (n=3). Quantification of tumor sphere diameter measurements (n=27 total per condition; n=9 per replicate) from three-independent replicates are provided in the bar graph. Statistical significance was determined using an unpaired, two-tailed t-test (****, $P < 0.0001$). **G)** Bar graph showing the body weight of shControl + DOX and shACSS2 + DOX mice at the endpoint of study. **H)** Bar graph showing the body weight of Vehicle- and 15 mg/kg ACSS2i-treated mice at the endpoint of study. **I)** Bar graphs showing quantification of the percentage of total area staining positive for CD31 (left) or Periodic Acid Schiff (right) in tumor sections from shControl + DOX or shACSS2 + DOX mice. Statistical significance determined using an unpaired, two-tailed t-test with Welch's correction (**, P-value < 0.005; ***, P-value < 0.001). **J)** Bar graphs showing quantification of the percentage of total area staining positive for CD31 (left) or Periodic Acid Schiff (right) in tumor sections from mice treated with Vehicle or 15 mg/kg ACSS2i. Statistical significance determined using an unpaired, two-tailed t-test with Welch's correction (*, P-value < 0.05). Data are represented as mean +/- SD.

Figure S2

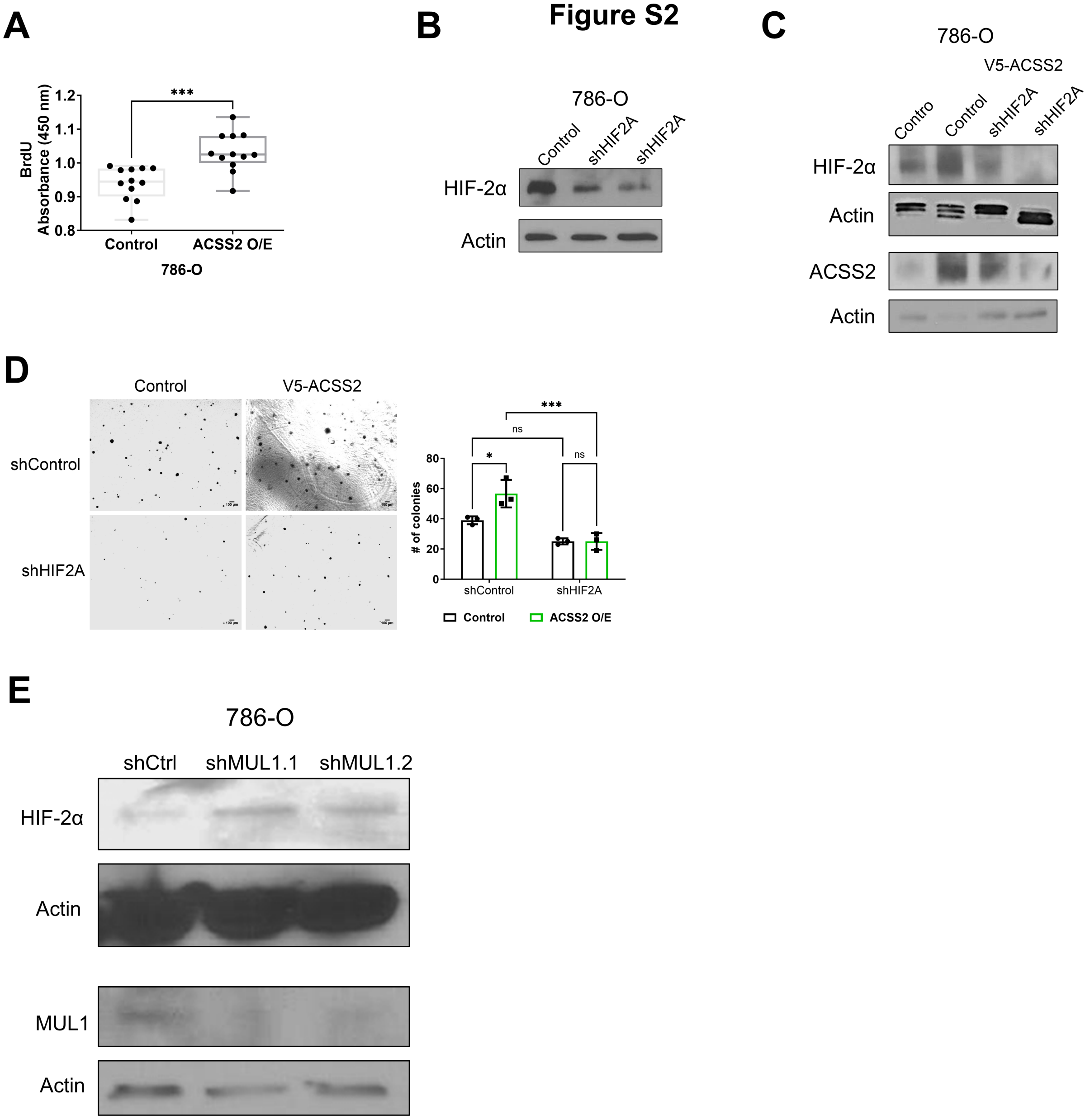

Figure S2. ACSS2 activity regulates HIF-2α expression and ccRCC growth, Related to Figures 4 and 5. A) Box and whisker plot showing the absorbance values detected at OD450 nm of BrdU ELISA assays performed on 786-O cells transduced pLX304 empty vector or pLX304 V5-ACSS2 overexpression vector. Statistical significance was determined using an unpaired, two-tailed t-test (***, $P < 0.001$). **B)**

Western blot analysis of HIF-2α (n=2) and Actin (n=2) in 786-O cells transduced with shControl or shHIF2A. **C)** Western blot analysis of HIF-2α (n=2), ACSS2 (n=2), and Actin (n=2) in the 786-O cells stably overexpressing V5-ACSS2 (bottom) transduced with shControl or shHIF2A targeting constructs. **D)** Representative images taken at day 21 of an anchorage-independent growth assay performed using 786-O control or V5-ACSS2 cells transduced express shControl or shHIF2A (n=3 for all conditions). Bar graph showing the number of colonies formed for each condition from three-independent experiments. Statistical significance determined using two-way ANOVA and Tukey's multiple comparisons test (*, P-value < 0.05; ***, P-value < 001). Data are represented as mean +/- SD. **E)** Western blot analysis for HIF-2α (n=2), MUL1 (n=2), and Actin (n=2) from 786-O cells transduced with shControl or shMUL1 constructs. Data are represented as mean +/- SD. Statistical significance was determined using a two-way ANOVA with Tukey's multiple comparisons test (*, P-value < 0.05; **, P-value < 0.01; ***, P-value < 0.001; ****, P-value < 0.0001).

Figure S3

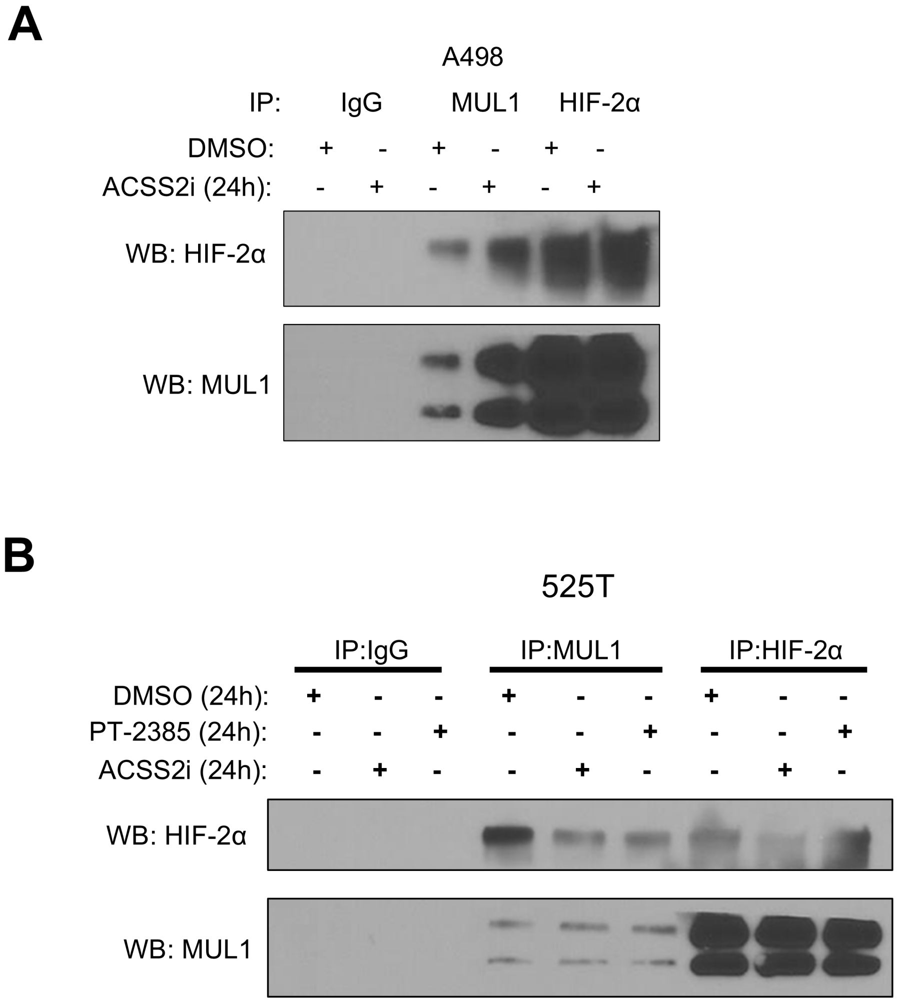

Figure S3. HIF-2α directly interacts with MUL1, Related to Figure 5. A) Western blot images for HIF-2α and MUL1 from immunoprecipitations performed in A498 cells treated with DMSO or 5 μM ACSS2i for 24 hours (n=2) using IgG, MUL1, and HIF-2α antibodies. **B)** Western blot images for HIF-2α and MUL1 from immunoprecipitations performed in patient-derived cancer cells treated with DMSO, 5 μM ACSS2i, or 10 μM PT-2385 for 48 hours (n=1) using IgG, MUL1, and HIF-2α antibodies.

Figure S4

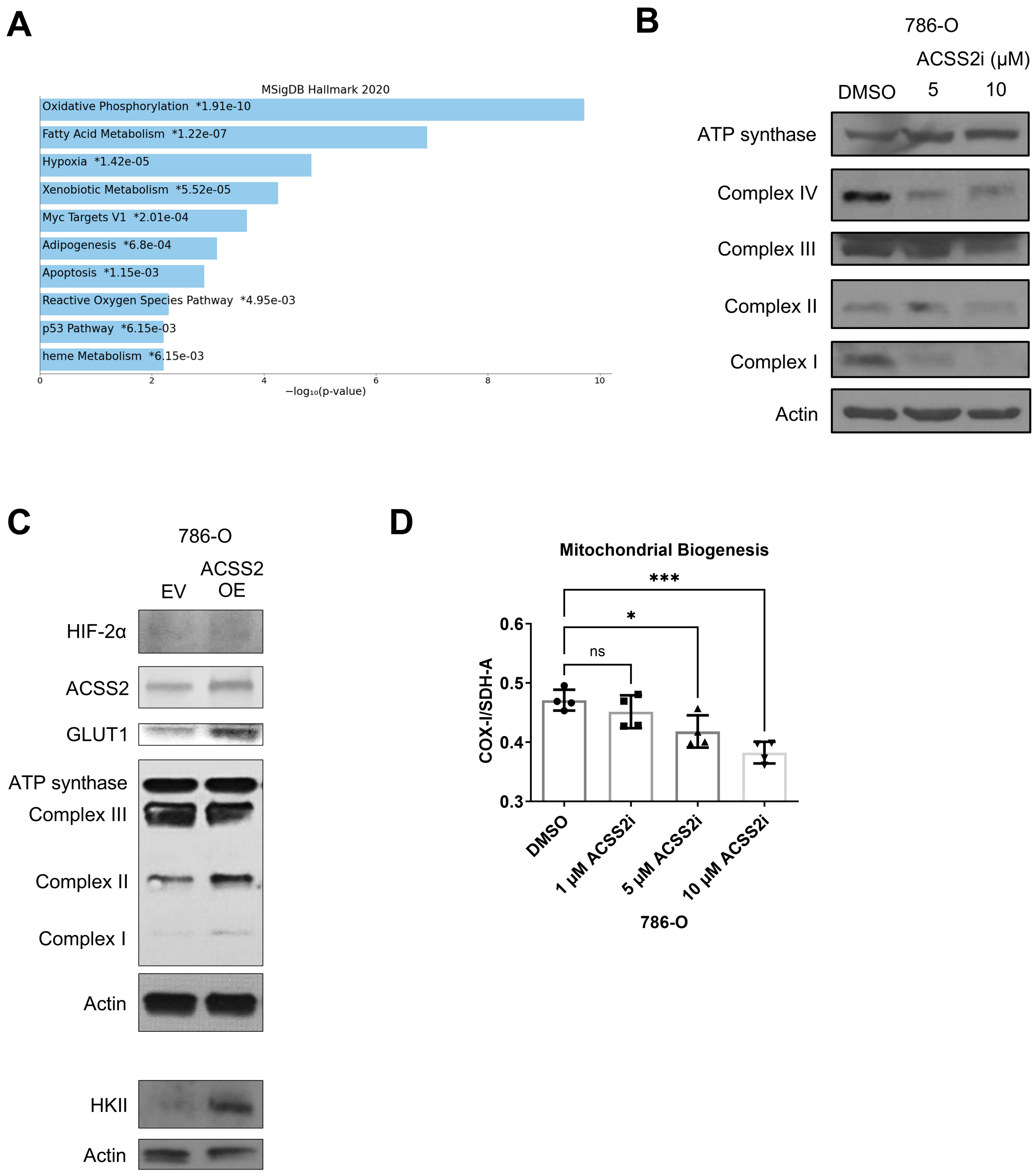

Figure S4. Regulation of OXPHOS by ACSS2, Related to Figure 8. A) Histogram depicting pathway hits from a gene-set enrichment analysis in ccRCC cells expressing ACSS2 from scRNA dataset. **B)** Western blot analysis for ATP synthase (n=3), Complex

IV (n=3), Complex III (n=3), Complex II (n=3), Complex I (n=3), and Actin (n=3) in 786-O cells treated with DMSO, 5 μM, or 10 μM ACSS2 inhibitor for 24 hours. **C)** Western blot analysis for HIF-2α (n=3), ACSS2 (n=3), GLUT1 (n=2), HKII (n=2) ATP synthase (n=3), Complex IV (n=3), Complex III (n=3), Complex II (n=3), Complex I (n=3), and Actin (n=6) in 786-O cells overexpressing EV or ACSS2. **D)** Bar graph with individual data points showing quantification of mitochondrial biogenesis in 786-O cells treated with DMSO, 1 μM, 5 μM or 10 μM ACSS2 inhibitor for 24 hours (n=4 for all conditions). Data is shown as the ratio of expression of COX-I to SDH-A quantified by wavelength detection at 405 nm (SDH-A) and 600 nm (COX-I). Statistical significance determined using an ordinary one-way ANOVA and Bonferroni's multiple comparisons test (*, P-value < 0.05; ***, P-value < 0.001). Data are represented as mean +/- SD.

Figure S5

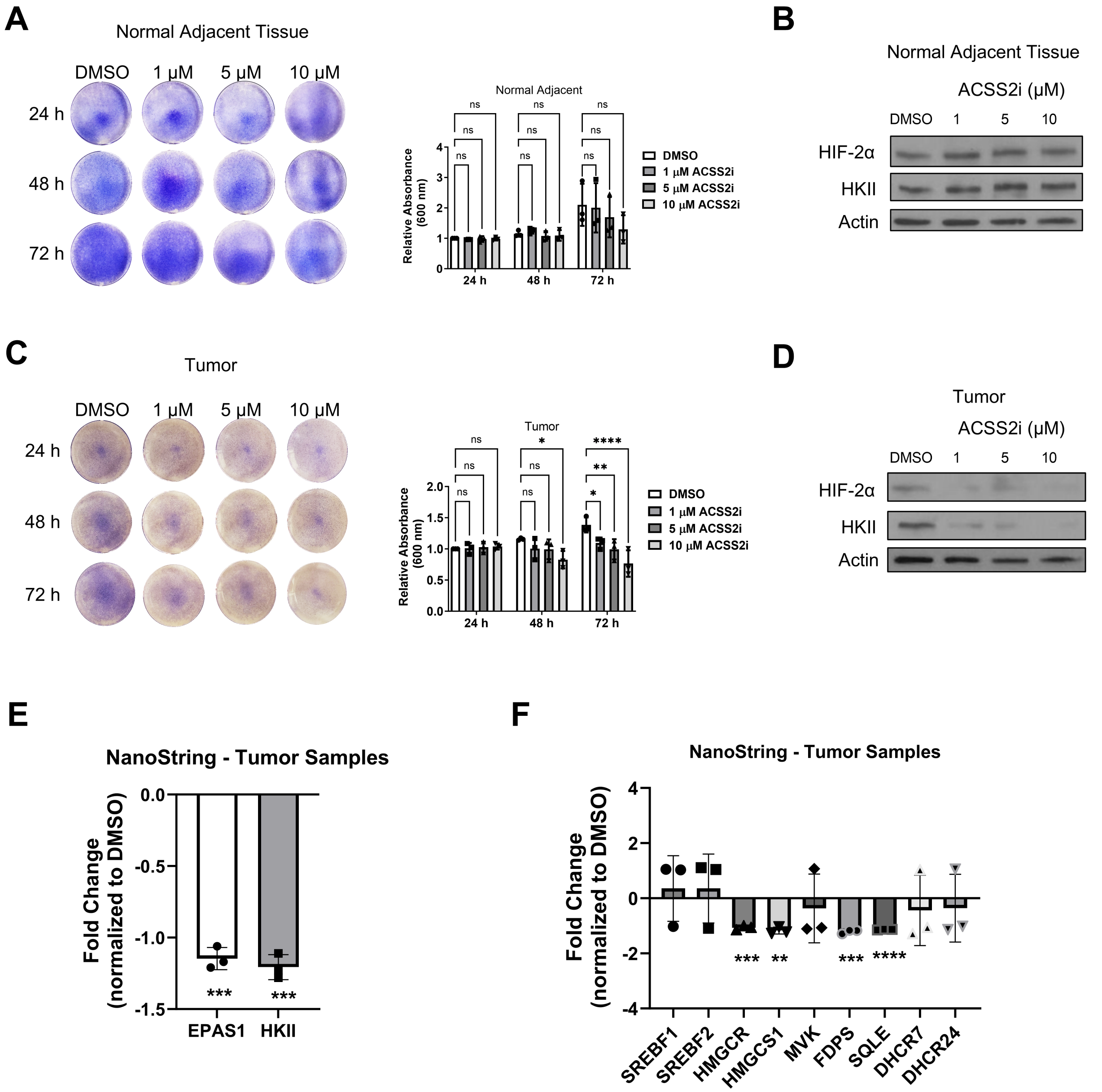

Figure S5. Effects of ACSS2 inhibition in clinical samples, Related to Figure 9. A) Representative images of crystal violet staining from a dose-response time-course treatment with DMSO (n=3) or ACSS2 inhibitor (n=3) in epithelial cells isolated from ccRCC patient's normal adjacent tissue. Bar graph representing the quantification of three biological replicate experiments. Statistical significance determined using two-way

ANOVA and Tukey's multiple comparisons test. **B)** Western blot images showing expression of HIF-2α and HKII in cells isolated from ccRCC patient normal adjacent tissue treated with DMSO, 1μM, 5 μM, or 10 μM ACSS2 inhibitor for 24 hours. **C)** Representative images of crystal violet staining from a dose-response time-course treatment with DMSO (n=3) or ACSS2 inhibitor (n=3) in epithelial cells isolated from ccRCC patient tumor sections. Bar graph representing the quantification of three biological replicate experiments. Statistical significance determined using two-way ANOVA and Tukey's multiple comparisons test (*, P-value < 0.05; **, P-value < 0.005; ****, P-value < 0.0001). **D)** Western blot images showing expression of HIF-2α and HKII in cells isolated from ccRCC patient cancer cells treated with DMSO, 1μM, 5 μM, or 10 μM ACSS2 inhibitor for 24 hours. **E)** Bar graph showing average fold change values for gene expression of *HK2* and *EPAS1* extracted from Nanostring experiment performed in ccRCC patient tumor samples treated with DMSO (n=3) or 5 μM ACSS2i (n=3) for 48 hours. Statistical significance determined by performing unpaired, two-tailed t-tests (***, P-value < 0.001). **F)** Bar graph showing average fold change values for gene expression of genes involved in the cholesterol biosynthesis pathway extracted from Nanostring experiment performed in ccRCC patient tumor samples treated with DMSO (n=3) or 5 μM ACSS2i (n=3) for 48 hours. Statistical significance determined by performing multiple unpaired, two-tailed t-tests (**, P-value < 0.01; ***, P-value < 0.001; ****, P-value < 0.0001). Data are represented as mean +/- SD.
