# Supplementary material for: HIF-2α expression and metabolic signaling require ACSS2 in clear cell renal cell carcinoma
Source: J Clin Invest. 2024 Jun 17;134(12):e164249. doi: 10.1172/JCI164249 (PMC11178540; doi:10.1172/JCI164249)

Uncropped/Unedited Full Blot Images

Figure 4

Figure 4, Panel B, Ab: ACSS2

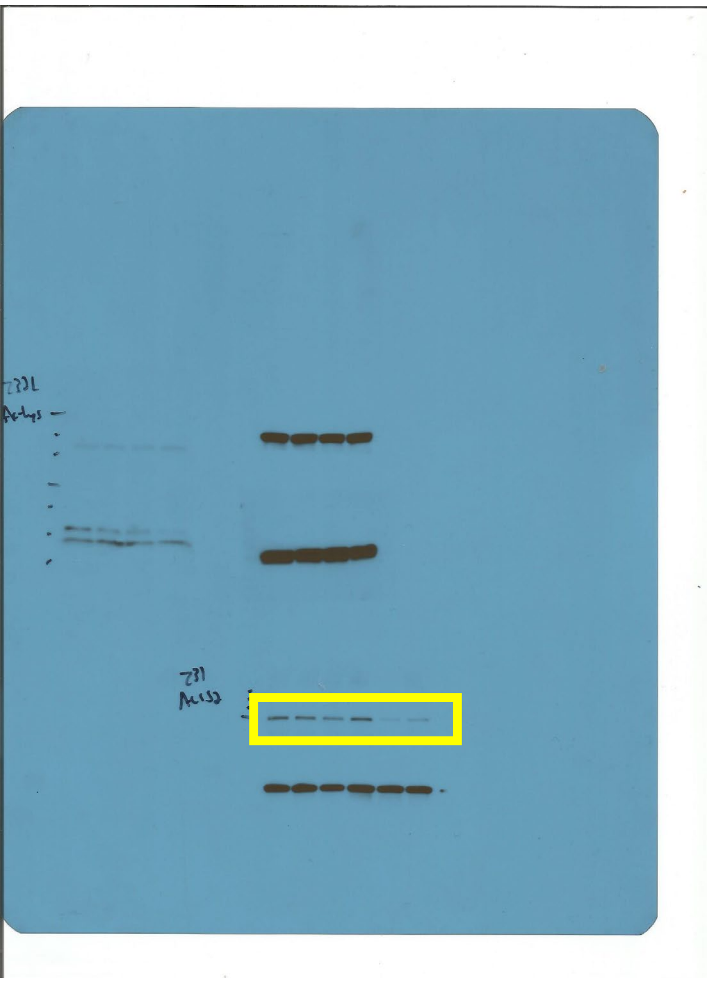

Figure 4, Panel B, Ab: HIF-2α

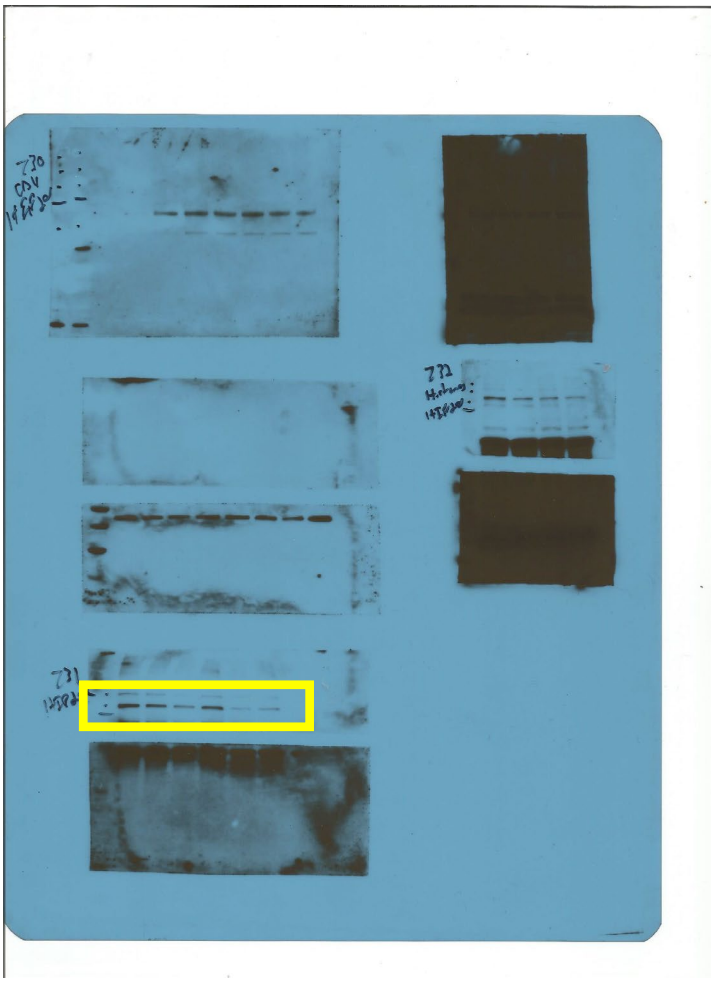

Figure 4, Panel B, Ab: Actin

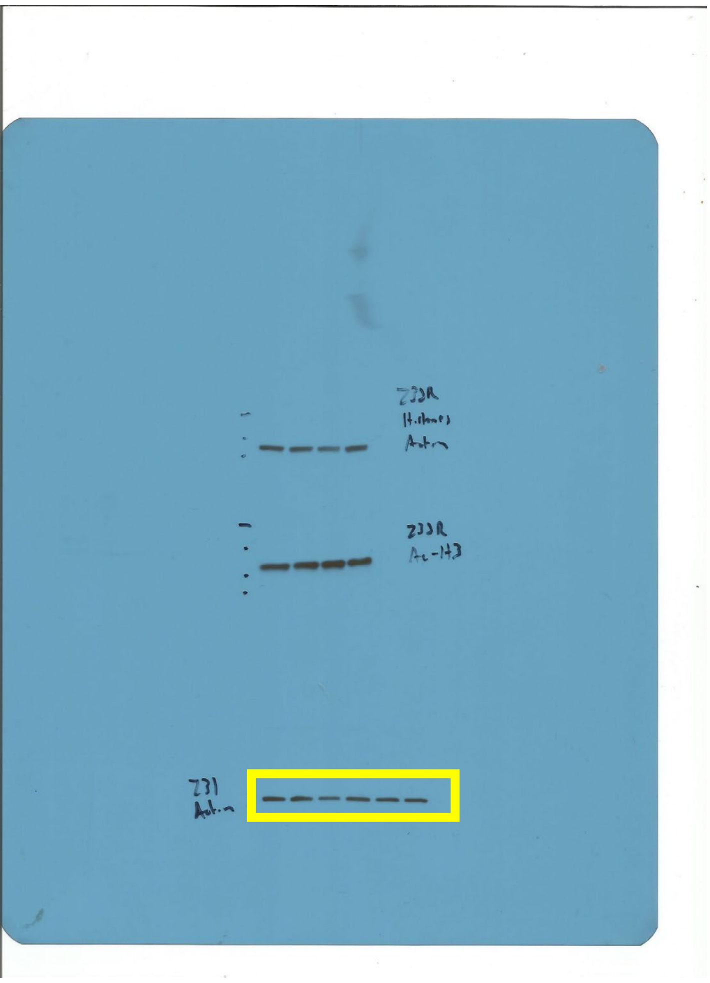

NEW FIGURE 4C

Figure 4, Panel C, Ab: HIF-2α for Epo gel

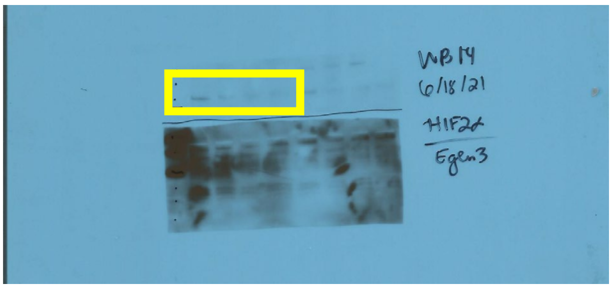

Figure 4, Panel C, Ab: VEGFR2

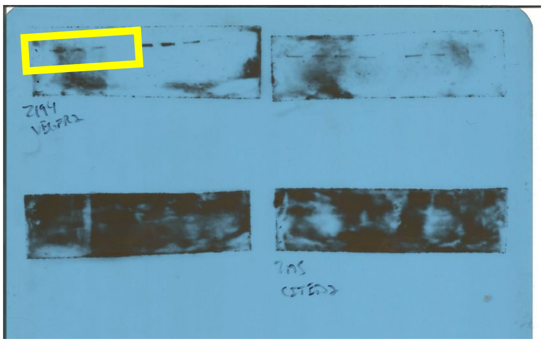

Figure 4, Panel C, Ab: Epo

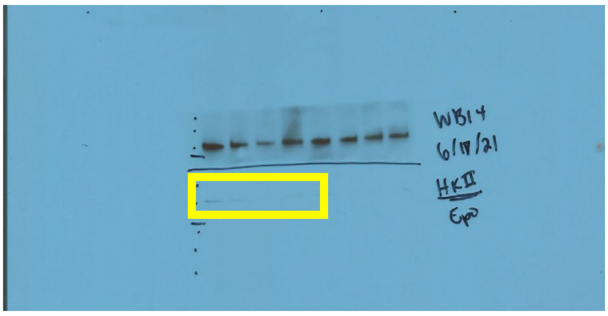

Figure 4, Panel C, Ab: HIF-2α

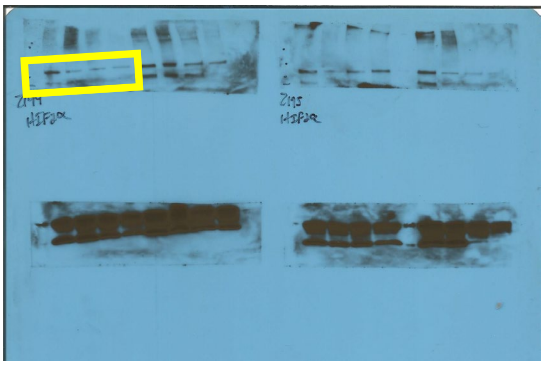

Figure 4, Panel C, Ab: Actin for Epo gel

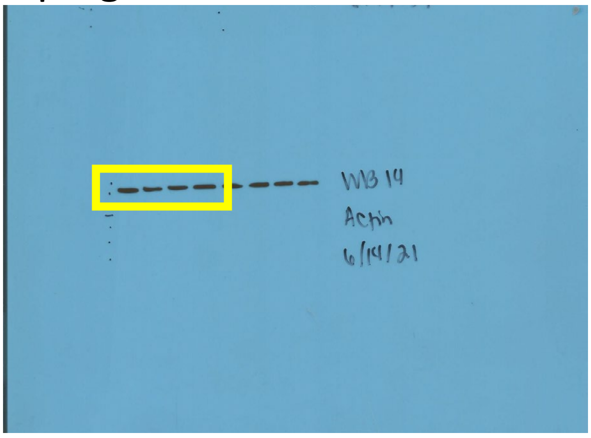

Figure 4, Panel C, Ab: Actin

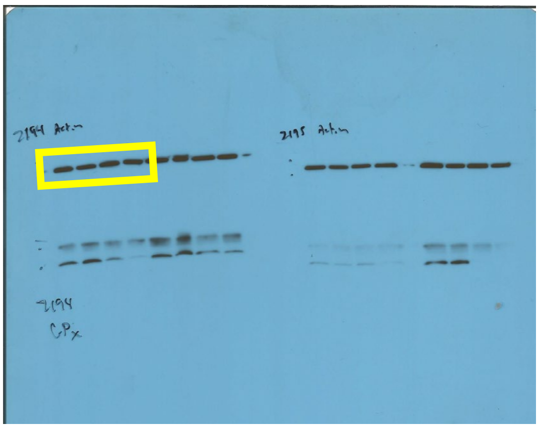

NEW FIGURE 4D

Figure 4, Panel D, Ab: Epo

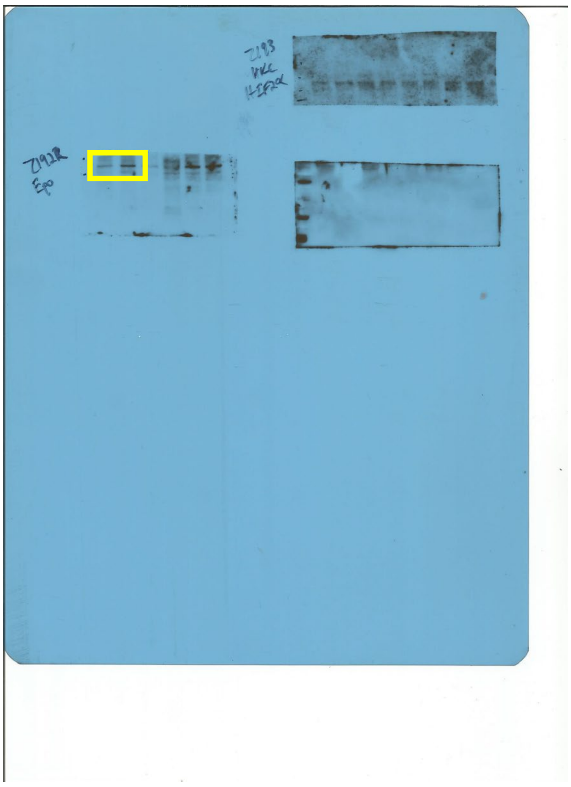

Figure 4, Panel D, Ab: ACSS2

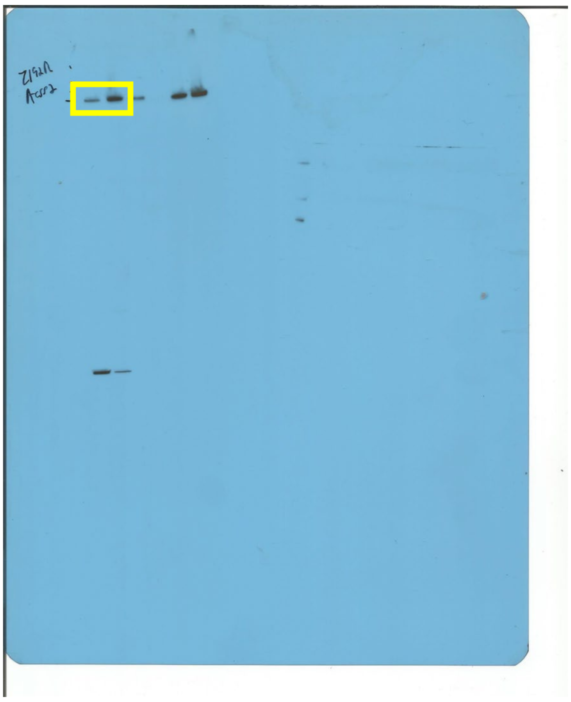

Figure 4, Panel D, Ab: VEGFR2

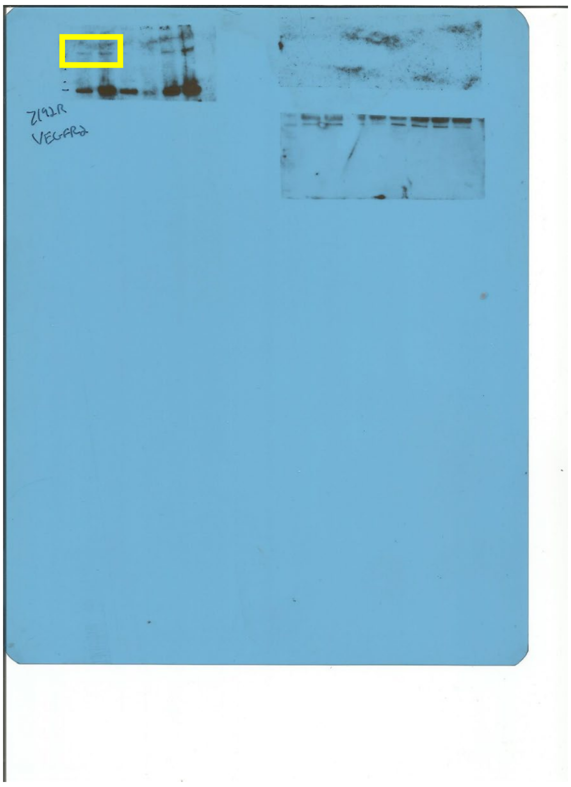

Figure 4, Panel D, Ab: Actin

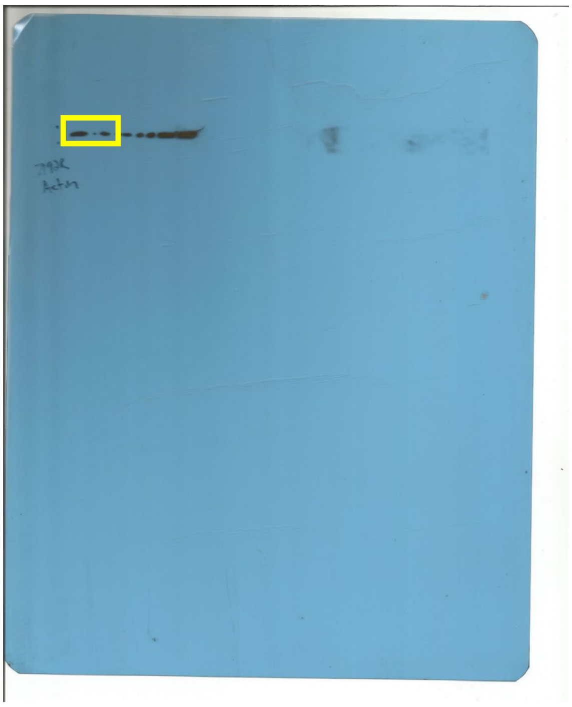

Figure 4, Panel D, Ab: HIF-2α

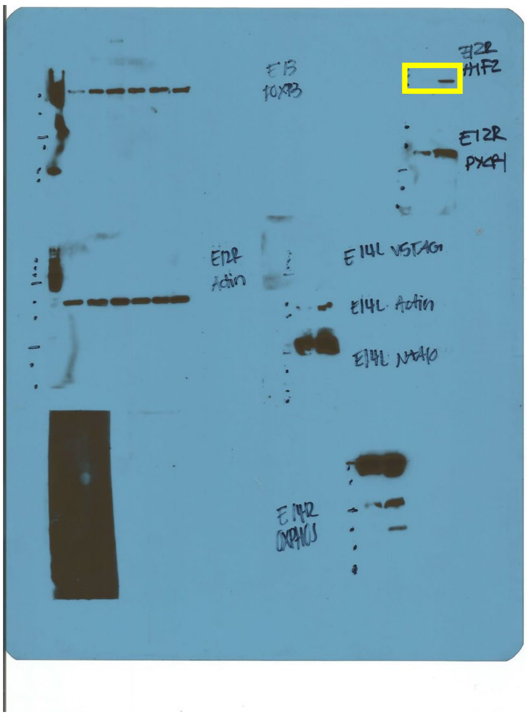

Figure 4, Panel D, Ab: Actin

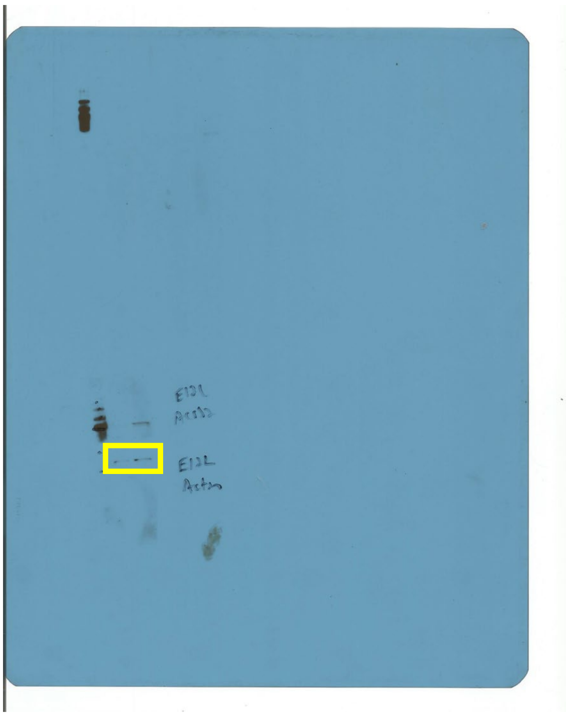

NEW FIGURE 4D (continued)

Figure 4, Panel D, Ab: EGLN3

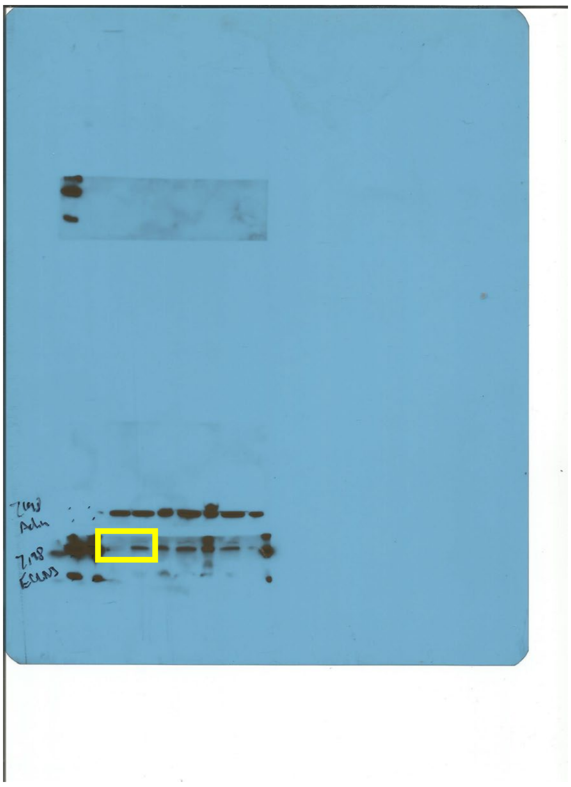

Figure 4, Panel D, Ab: Actin

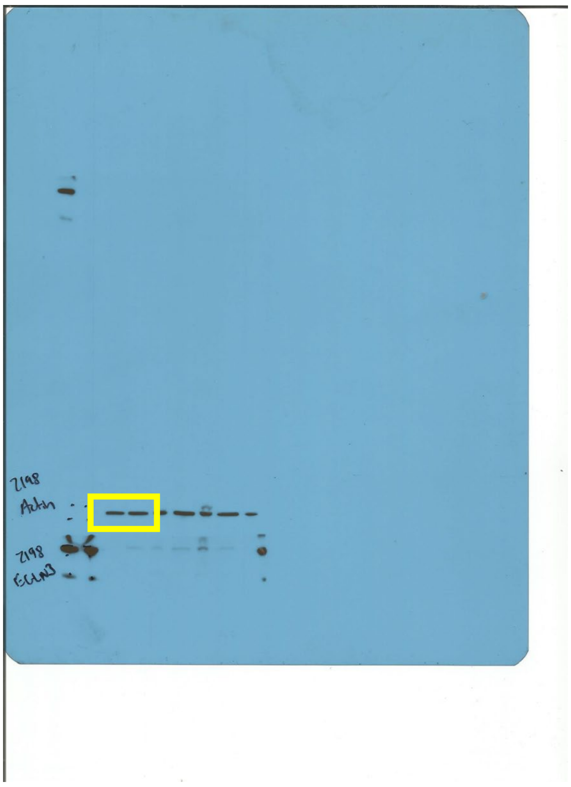

Figure 4, Panel E, Ab: HIF-2α

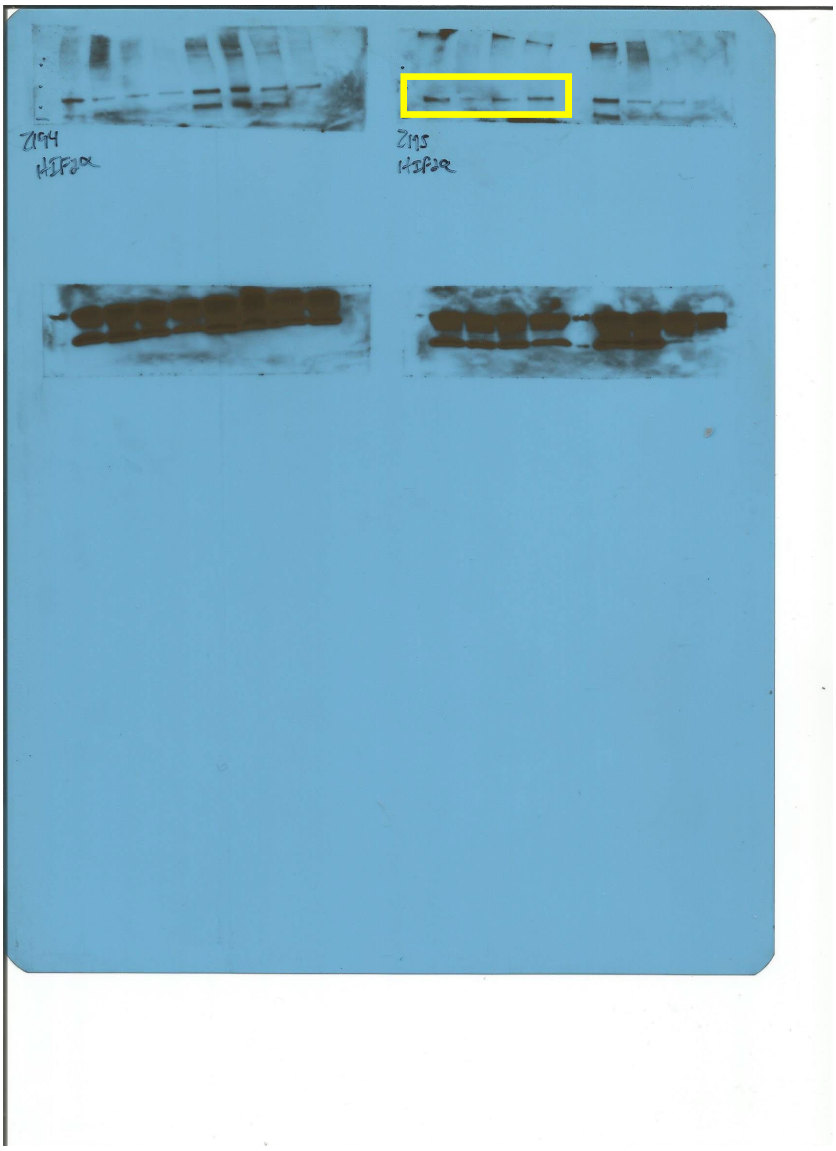

Figure 4, Panel E, Ab: Actin

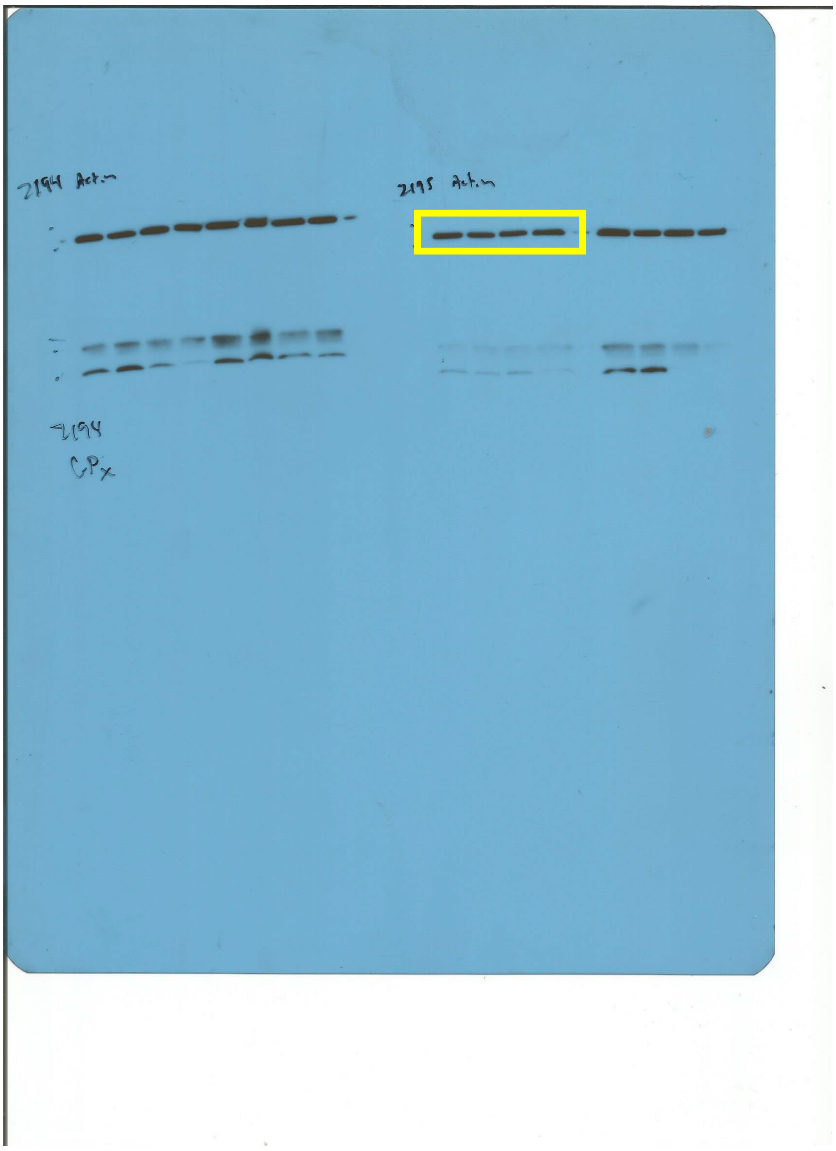

Figure 4, Panel F, Ab: LC3 A/B

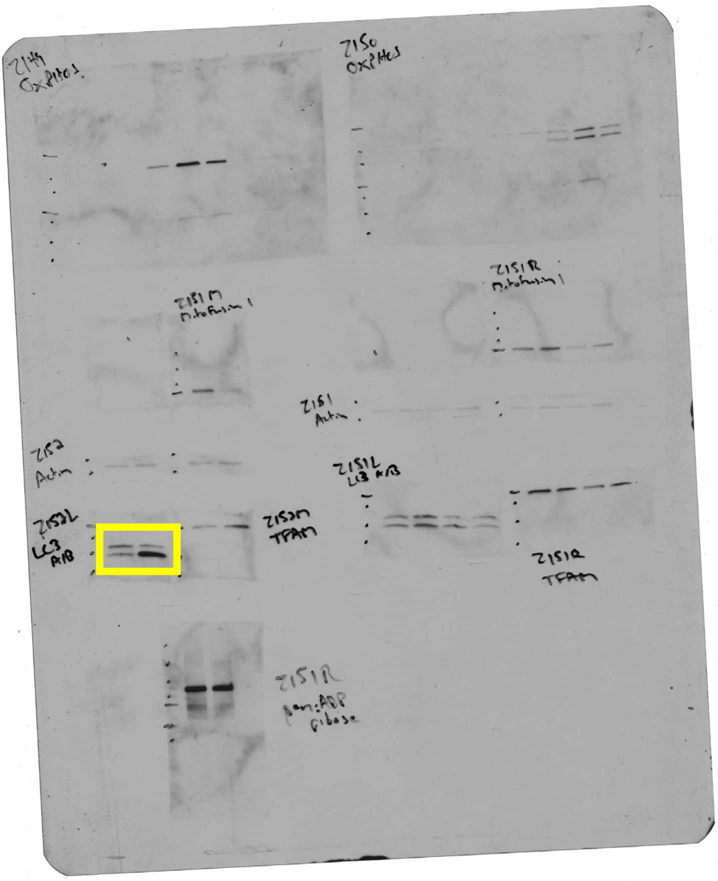

Figure 4, Panel F, Ab: pan-K48 PolyUb

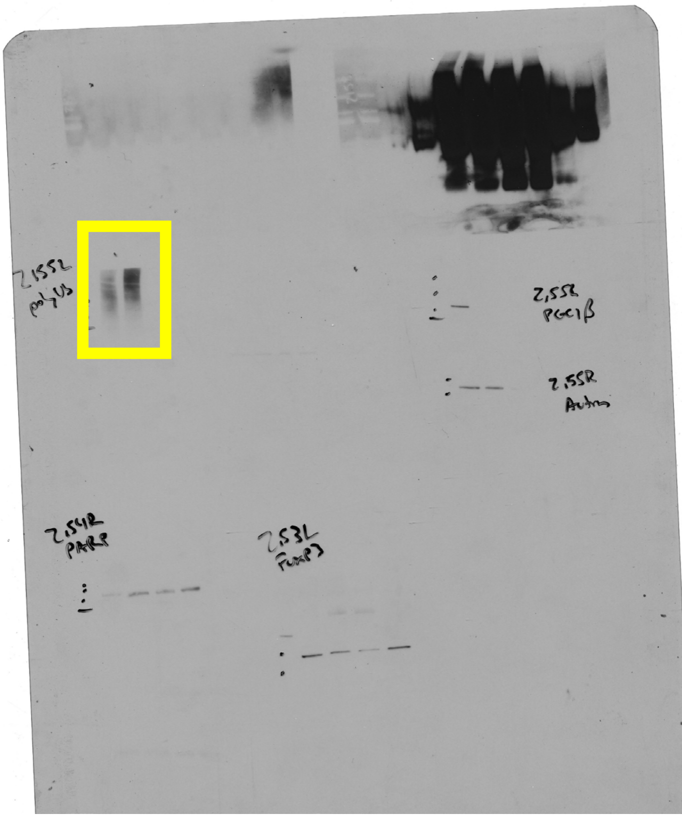

Figure 4, Panel F, Ab: Actin

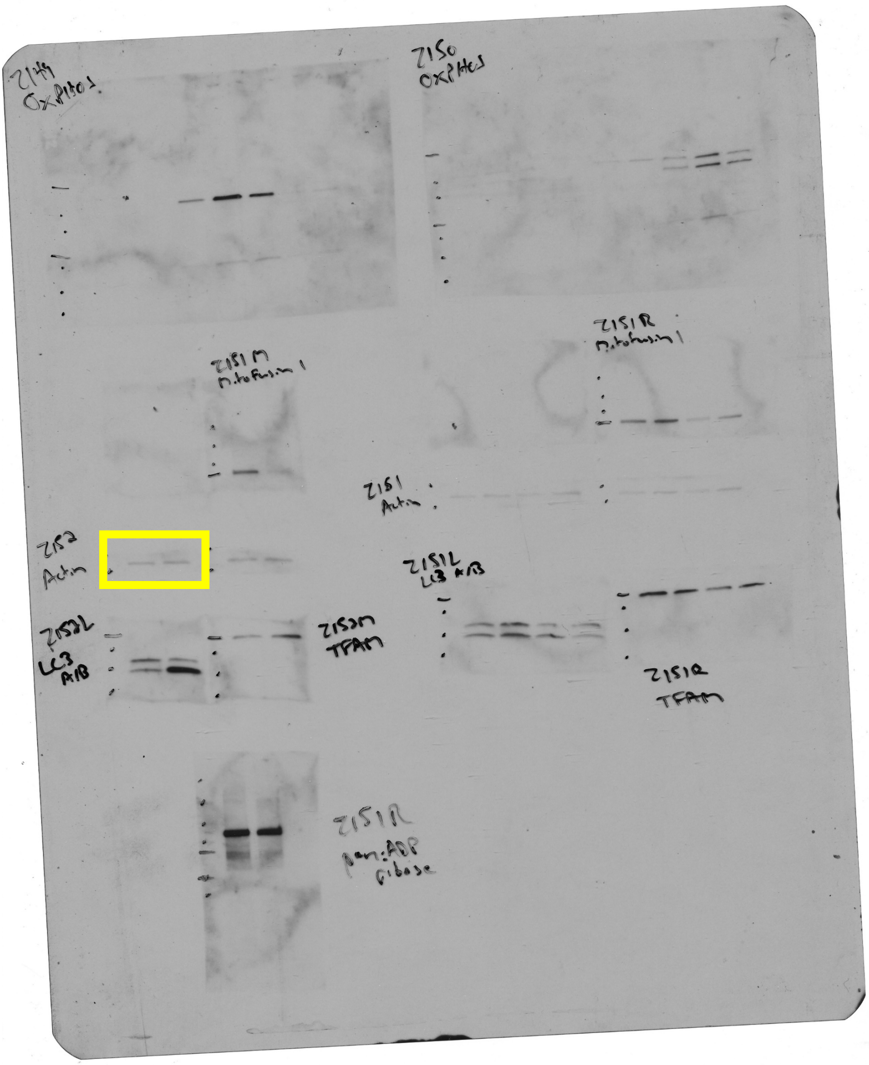

Figure 4, Panel F, Ab: MUL1

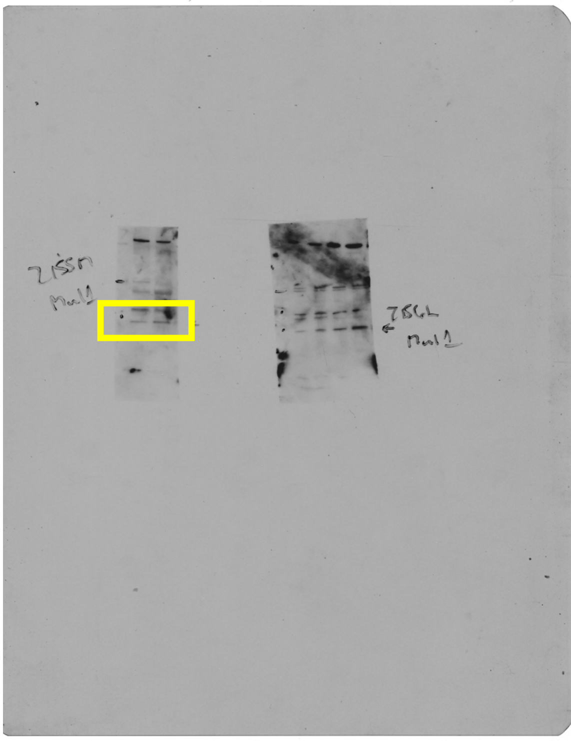

Figure 5

Figure 5, Panel A, Ab: HIF-2α

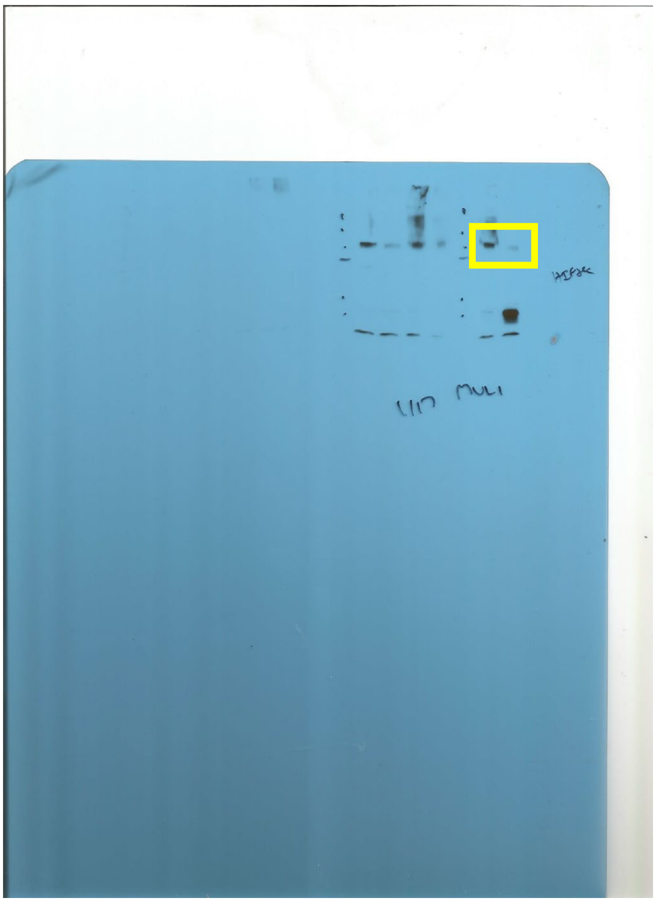

Figure 5, Panel A, Ab: ACSS2

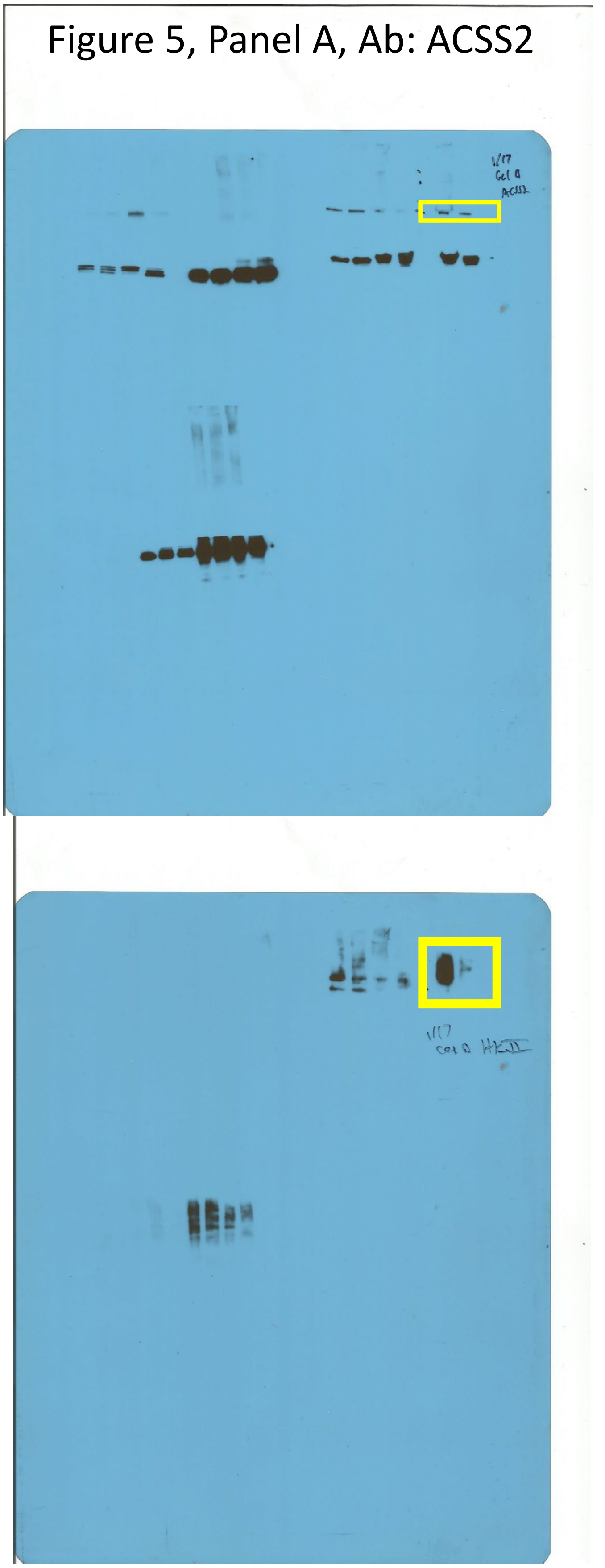

Figure 5, Panel A, Ab: Actin

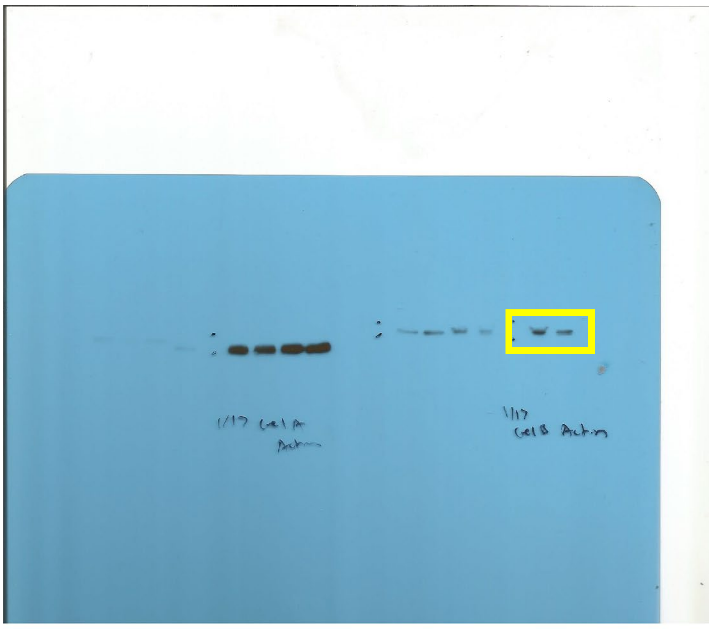

Figure 5, Panel A, Ab: MUL1

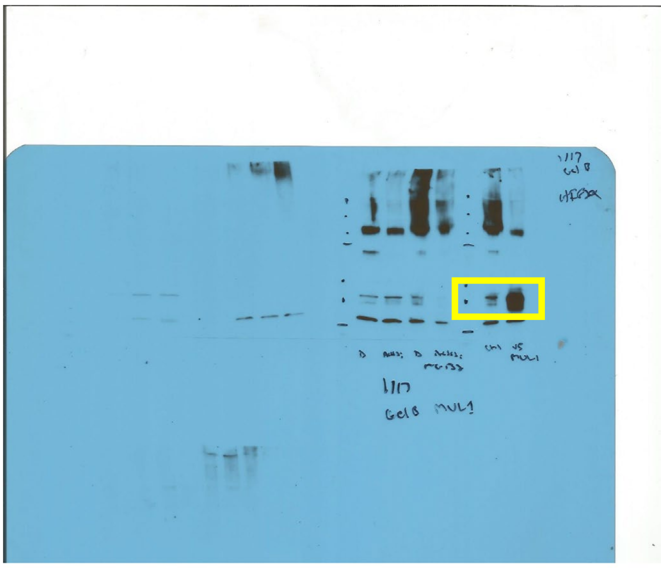

Figure 5, Panel B, Ab: HIF-2α

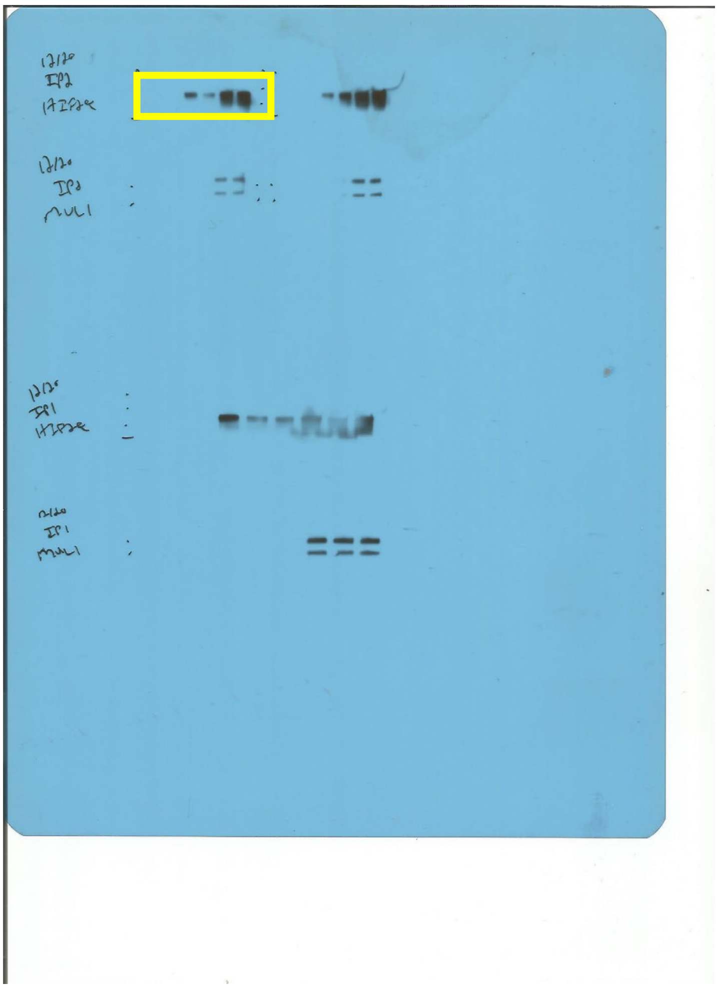

Figure 5, Panel B, Ab: MUL1

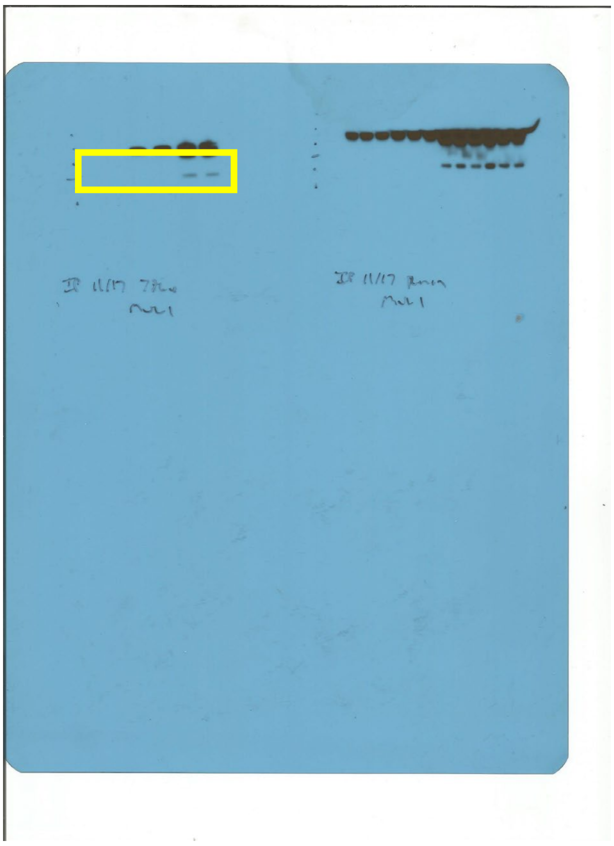

Figure 5, Panel B, Ab: MUL1
Dark exposure

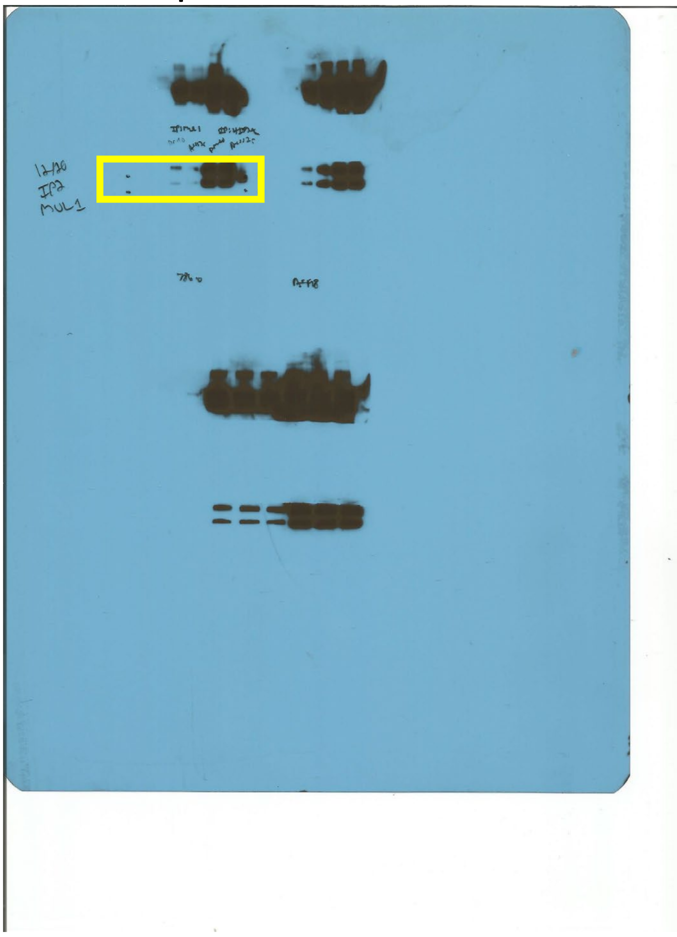

Figure S2

Figure S2, Panel B, Ab: HIF-2α

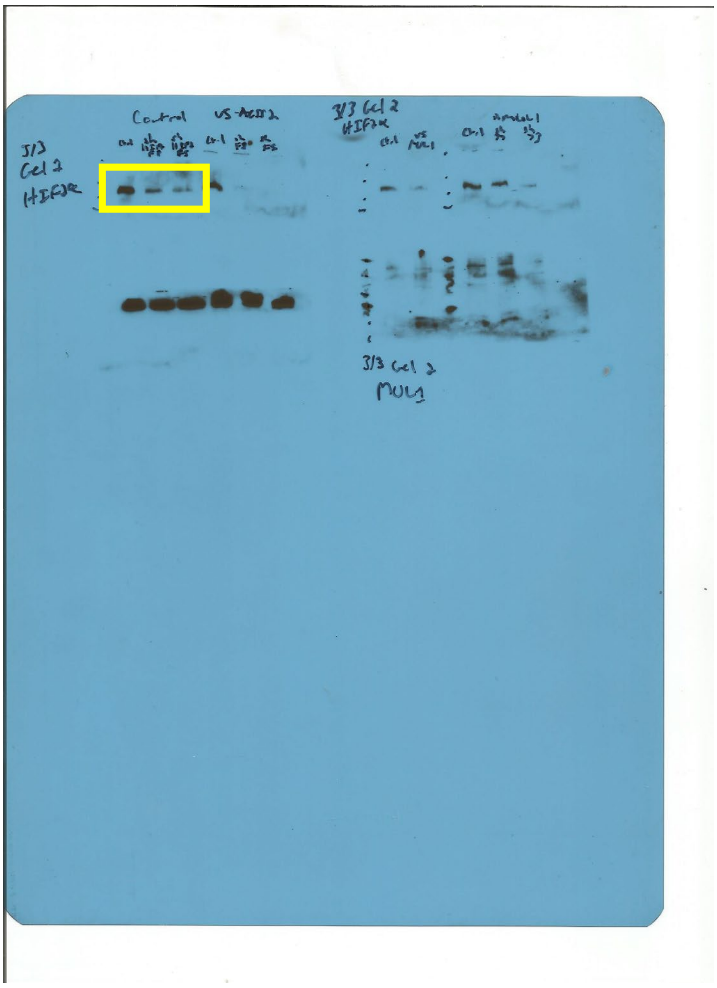

Figure S2, Panel B, Ab: Actin

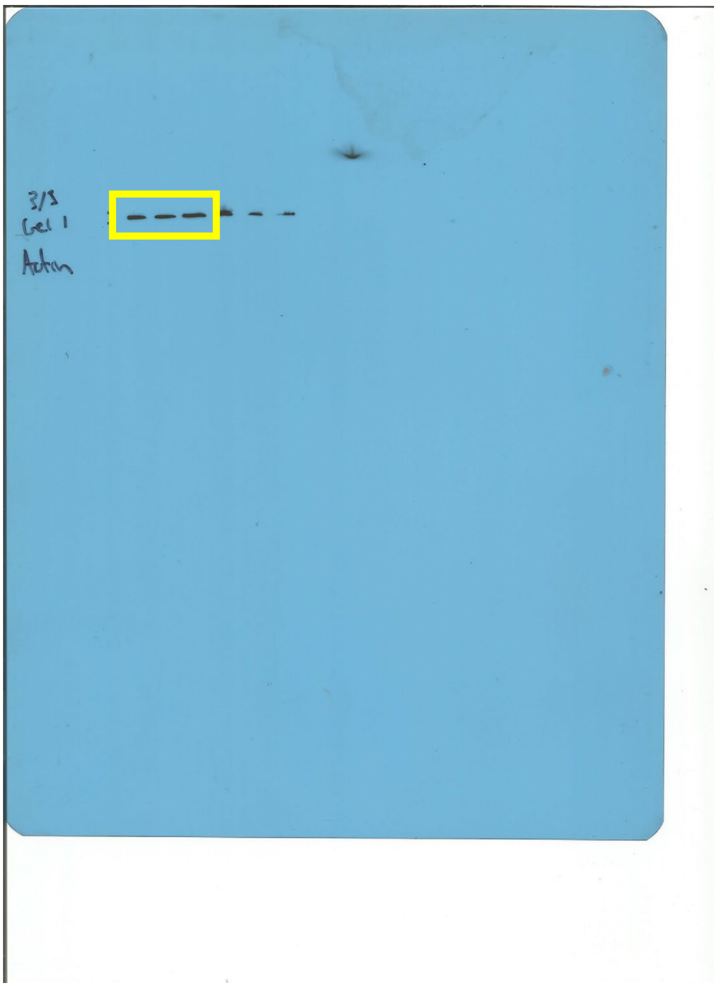

Figure S2, Panel C, Ab: HIF-2α

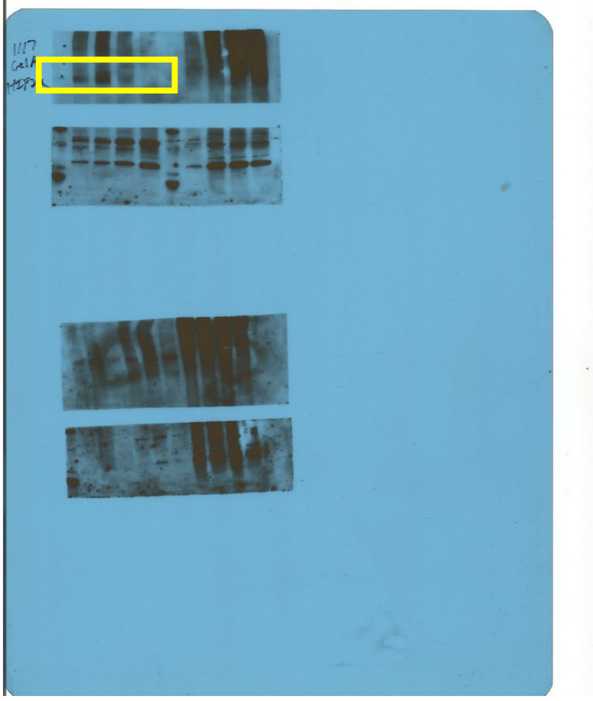

Figure S2, Panel C, Ab: ACSS2

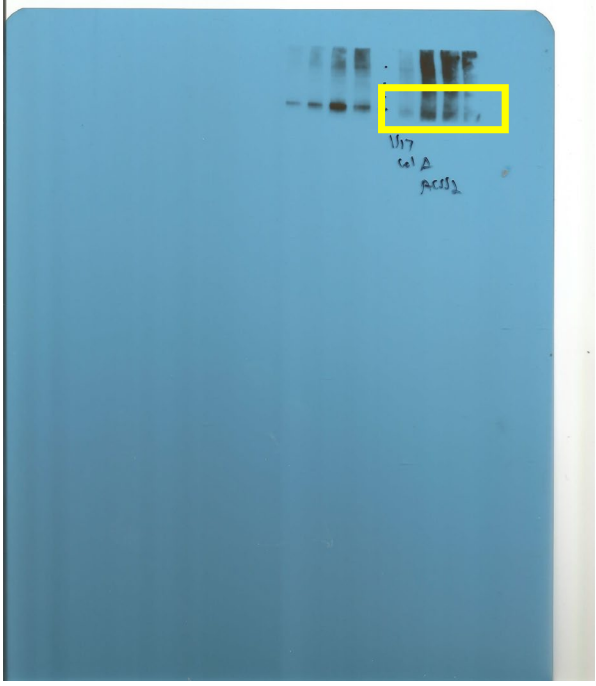

Ab: Actin; not used in figure

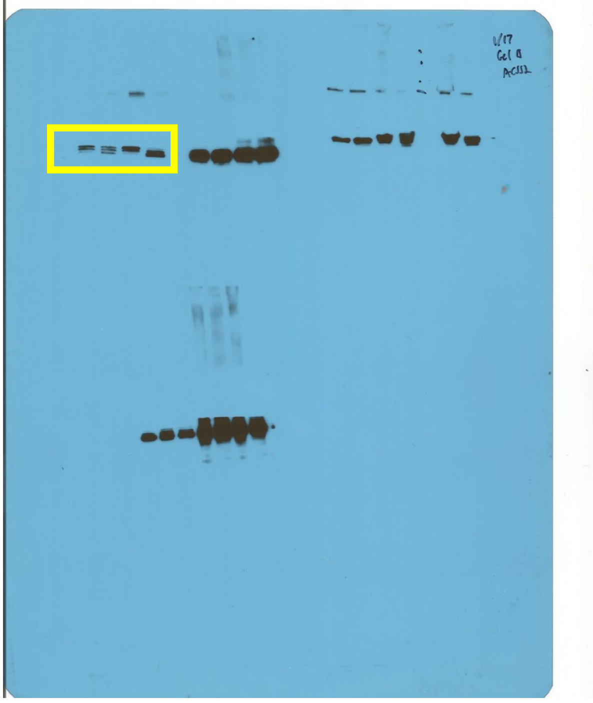

Figure S2, Panel C, Ab: Actin

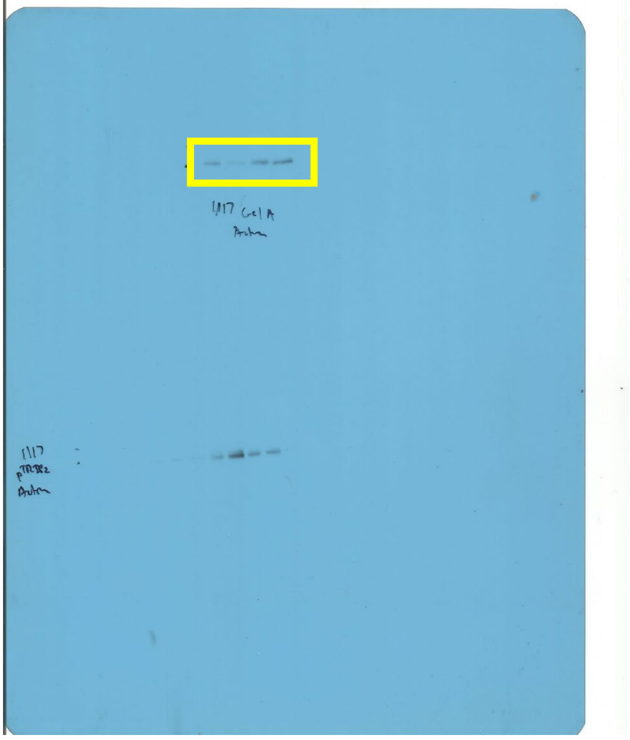

Lysate were loaded in duplicate on same gel.

Figure S2, Panel E, Ab: MUL1

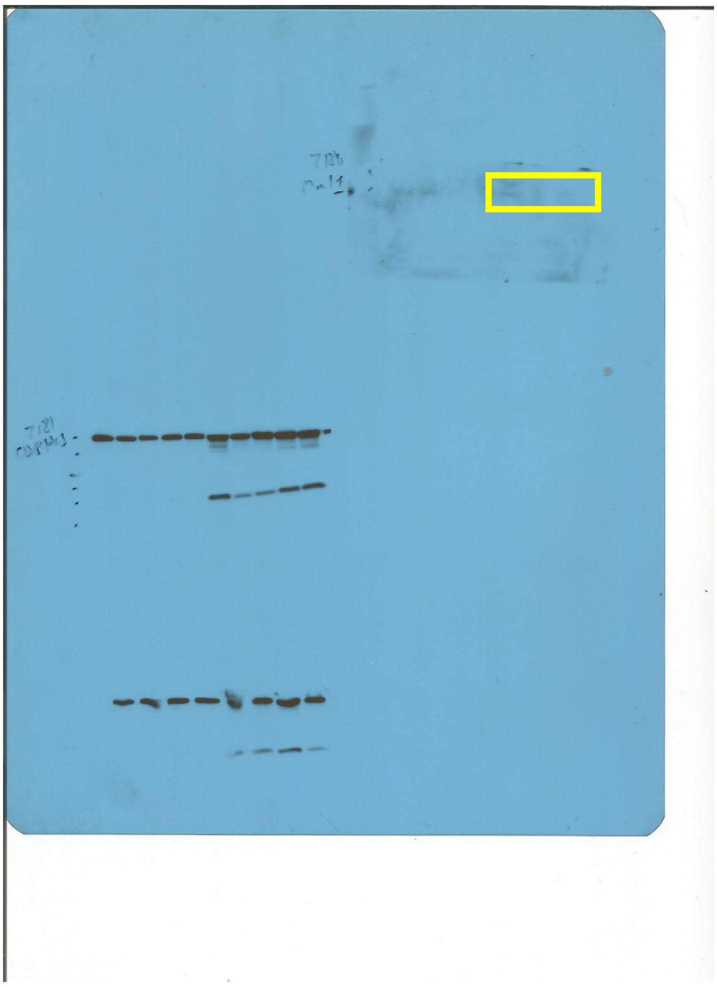

Figure S2, Panel E, Ab: HIF-2α

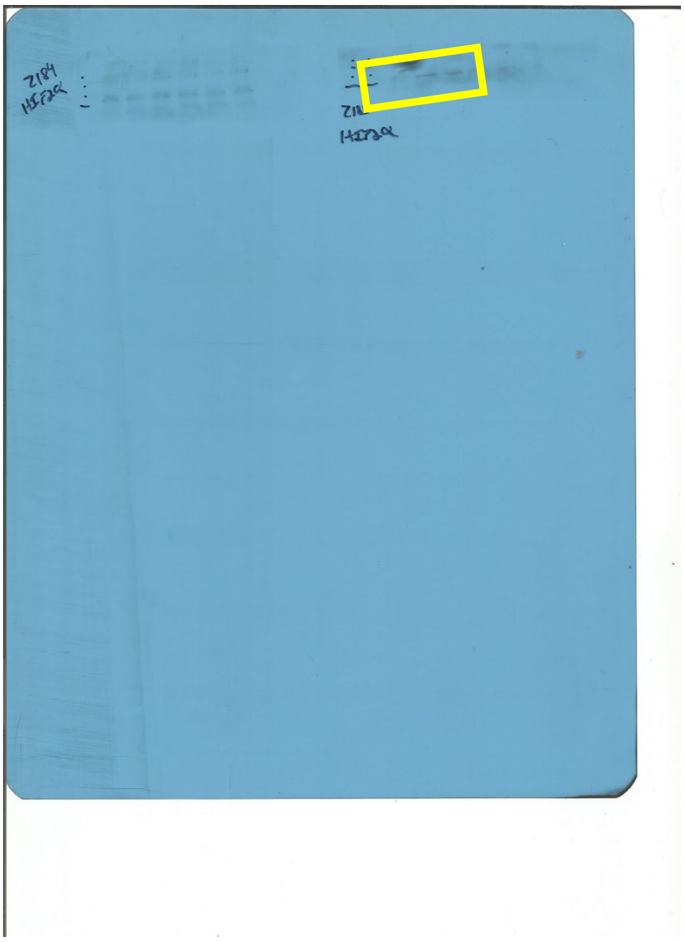

Figure S2, Panel E, Ab: Actin

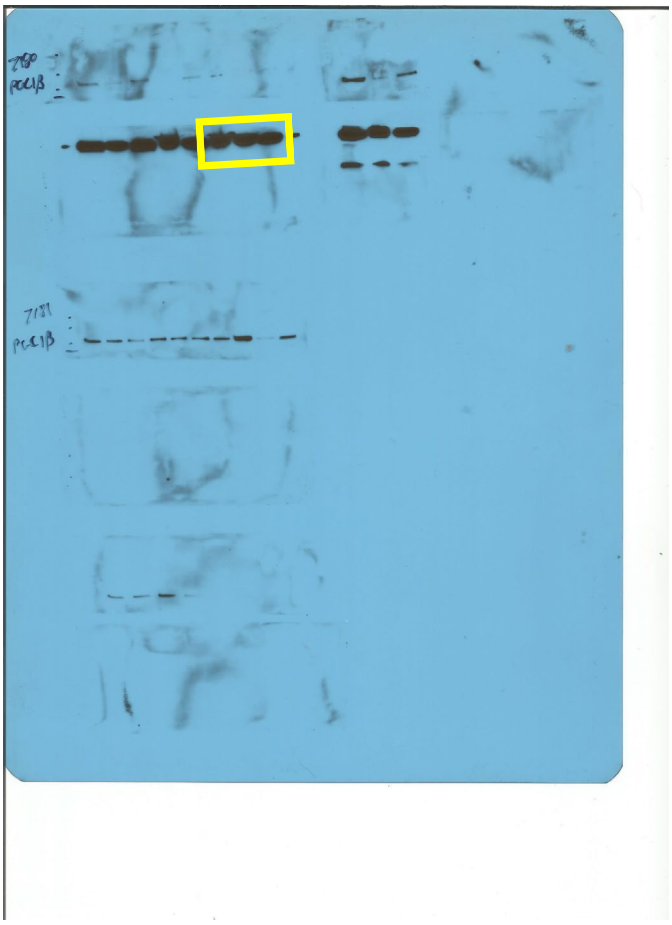

Figure S2, Panel E, Ab: Actin

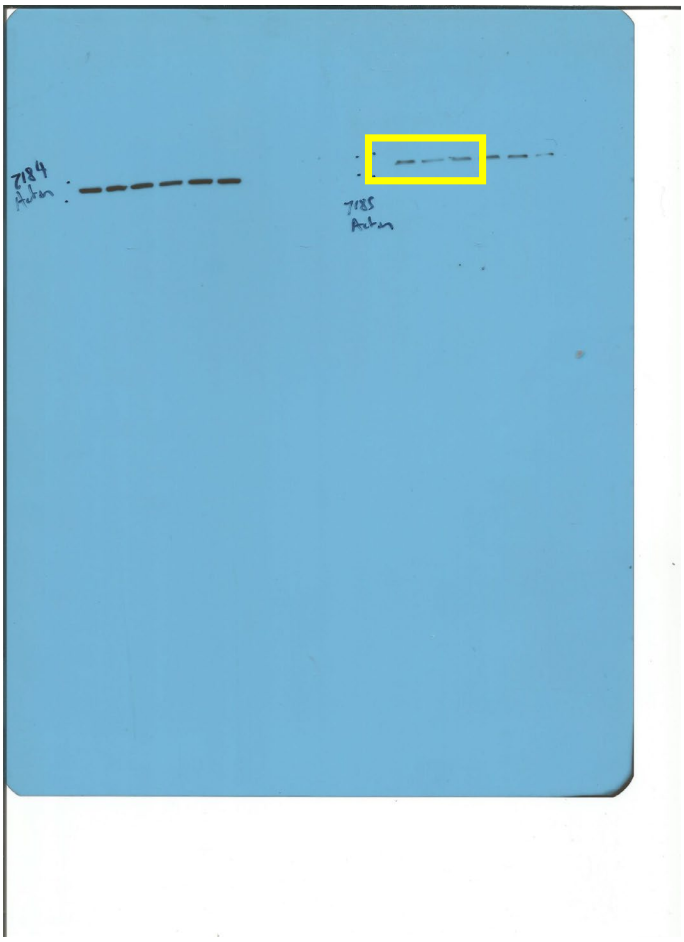

Figure S3

Figure S3, Panel A, Ab: MUL1

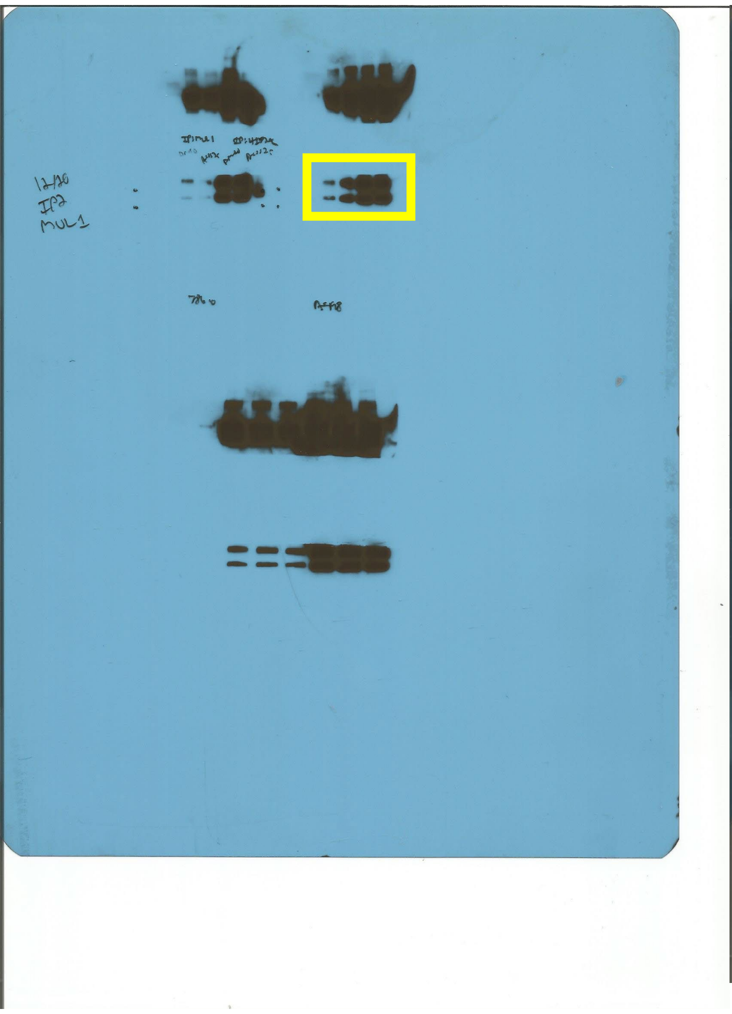

Figure S3, Panel A, Ab: HIF-2α

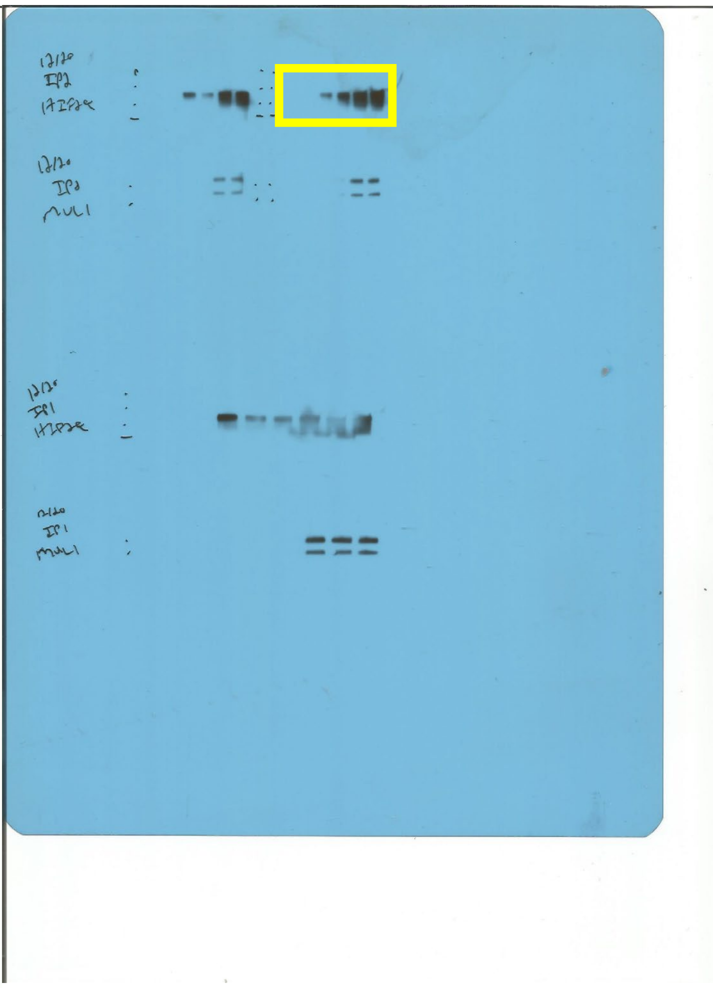

Figure S3, Panel B, Ab: MUL1

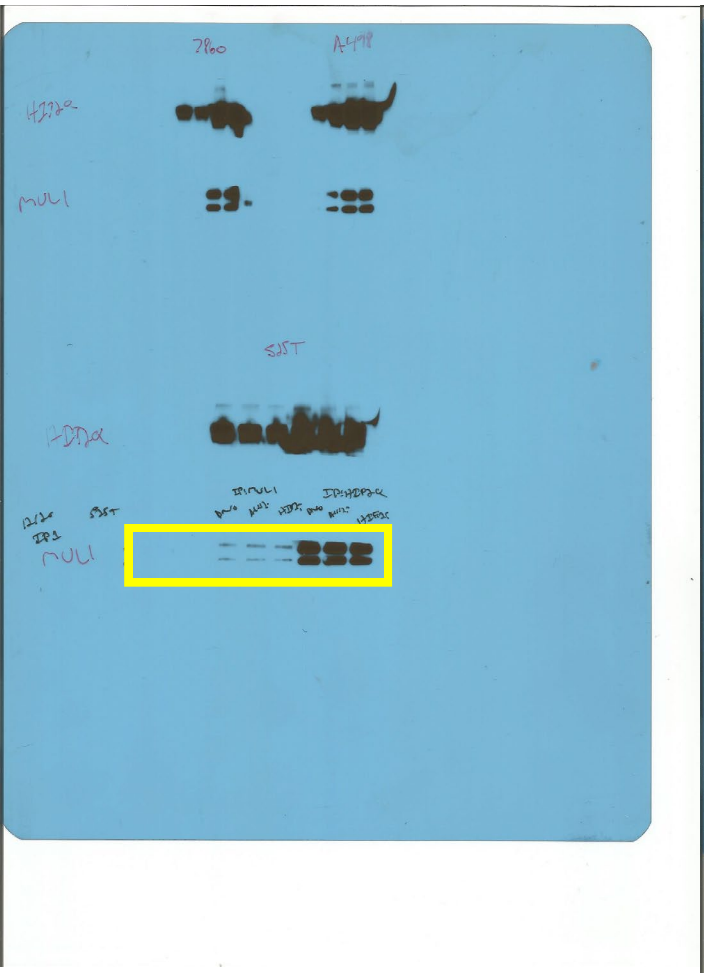

Figure S3, Panel B, Ab: HIF-2α

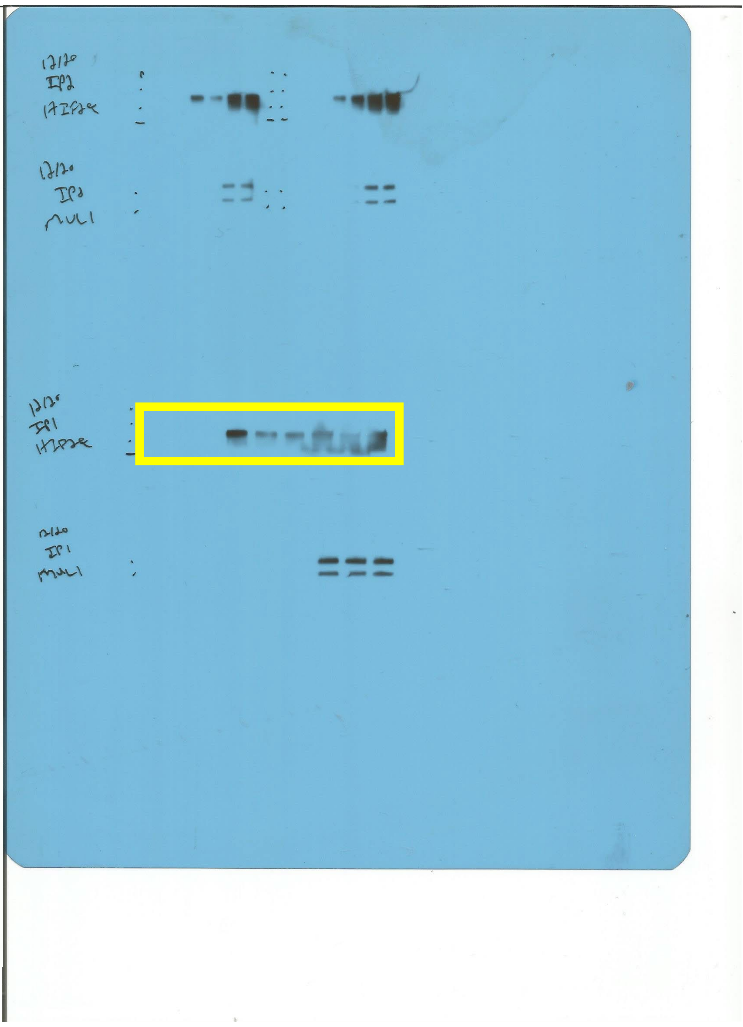

Figure 6

Figure 6, Panel C, Ab: Actin

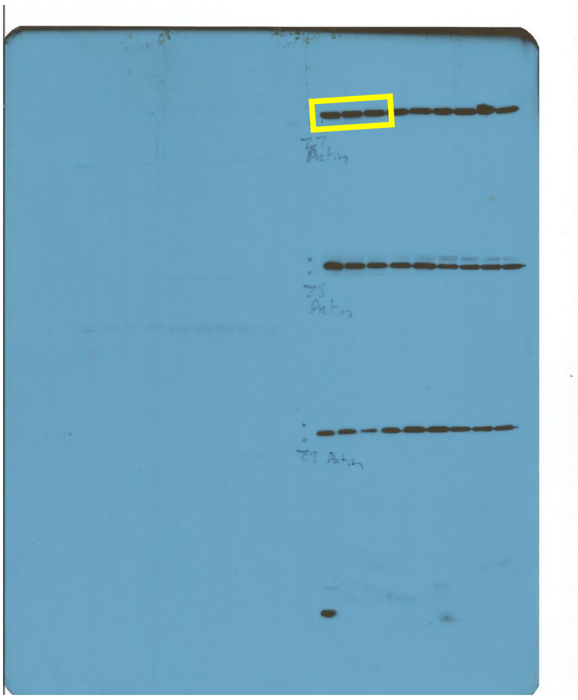

Figure 6, Panel C, Ab: HKII

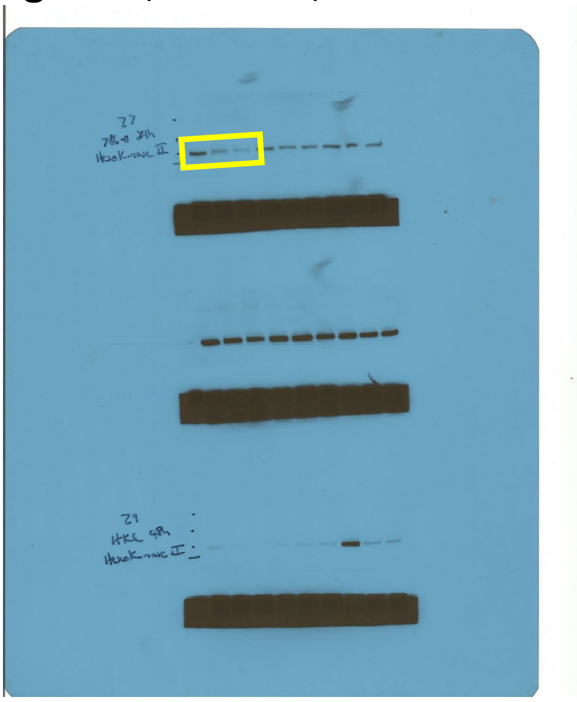

Figure 6, Panel C, Ab: SREBP1

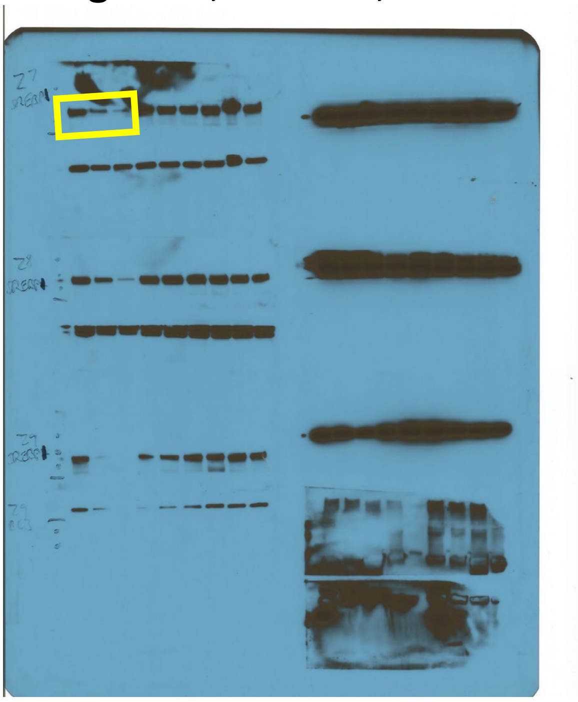

Figure 6, Panel C, Ab: GLUT1
Z10 = same lysate as Z7 w/o boiling to denature

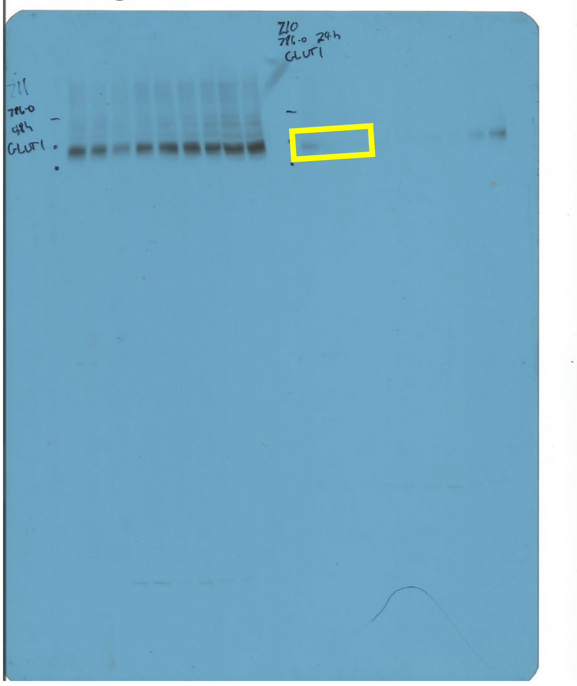

Figure 6, Panel C, Ab: SREBP2

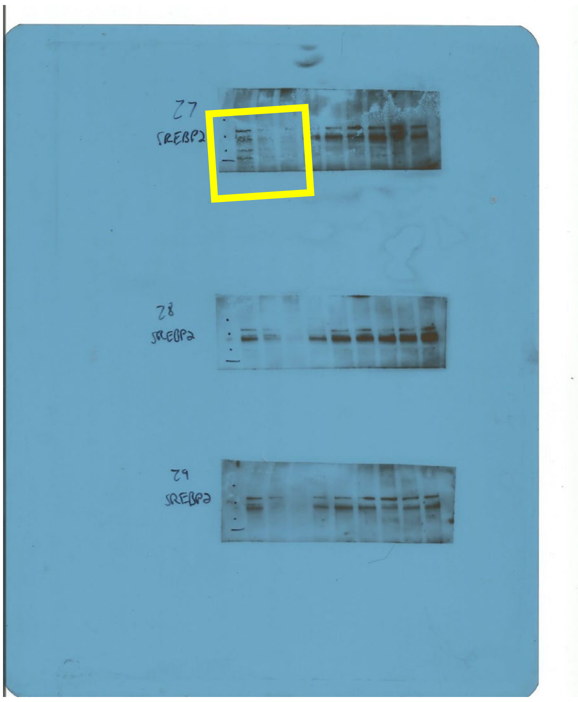

Figure S4

Figure S4, Panel B, Ab: Actin

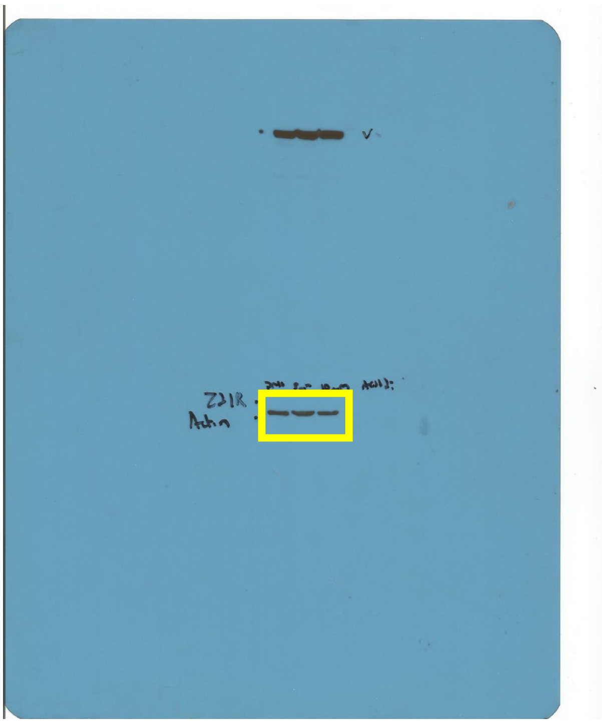

Figure S4, Panel B, Ab: ATP Synthase

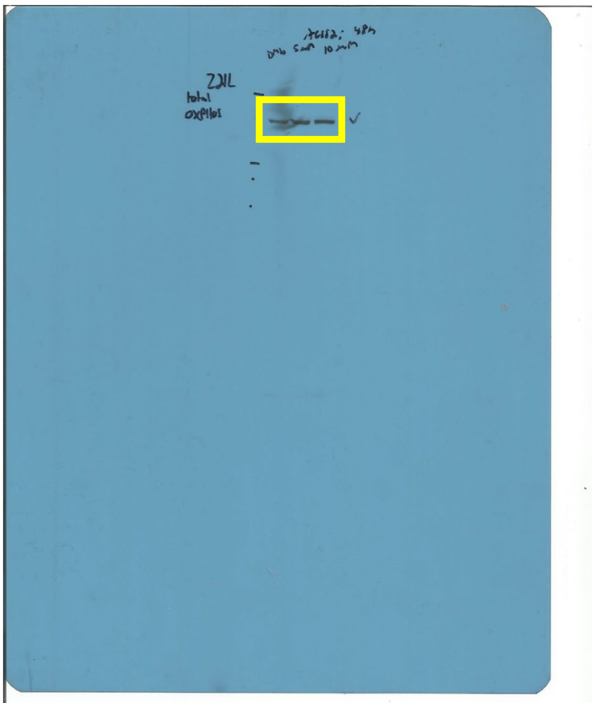

Figure S4, Panel B, Ab: Complex III

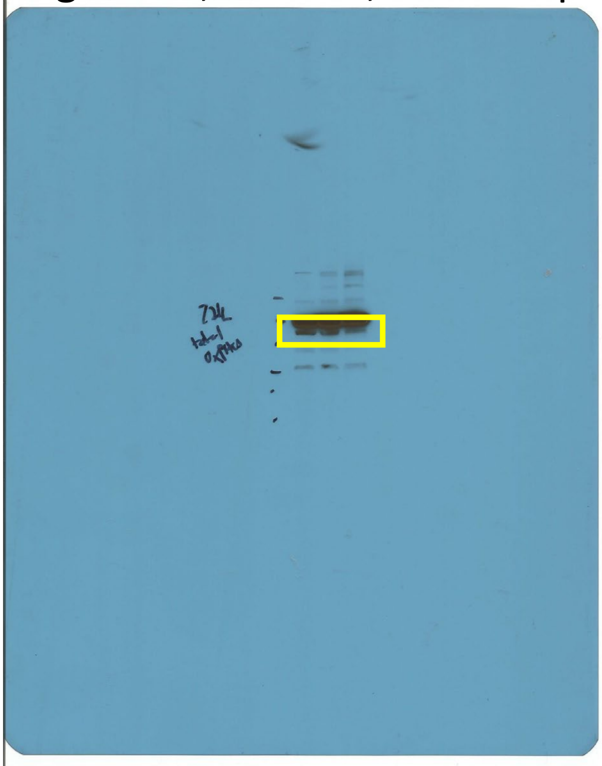

Figure S4, Panel B, Ab: Complex IV

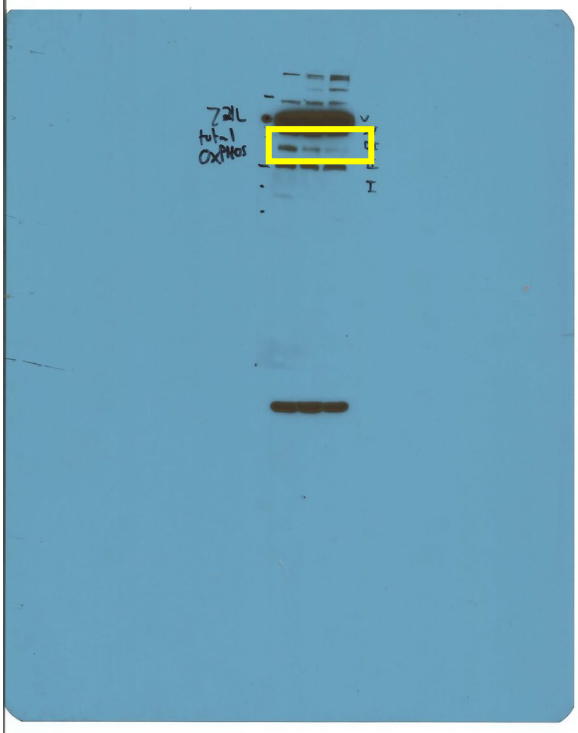

Figure S4, Panel B, Ab: Complex II

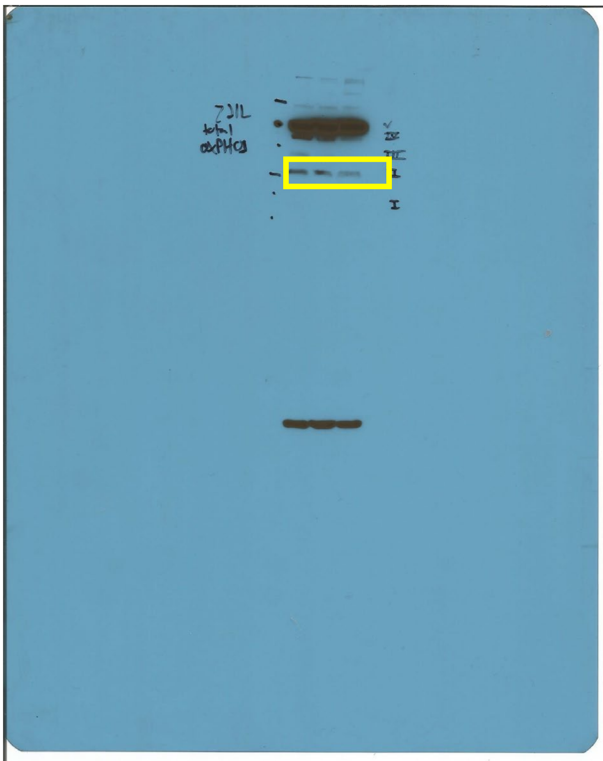

Figure S4, Panel B, Ab: Complex I

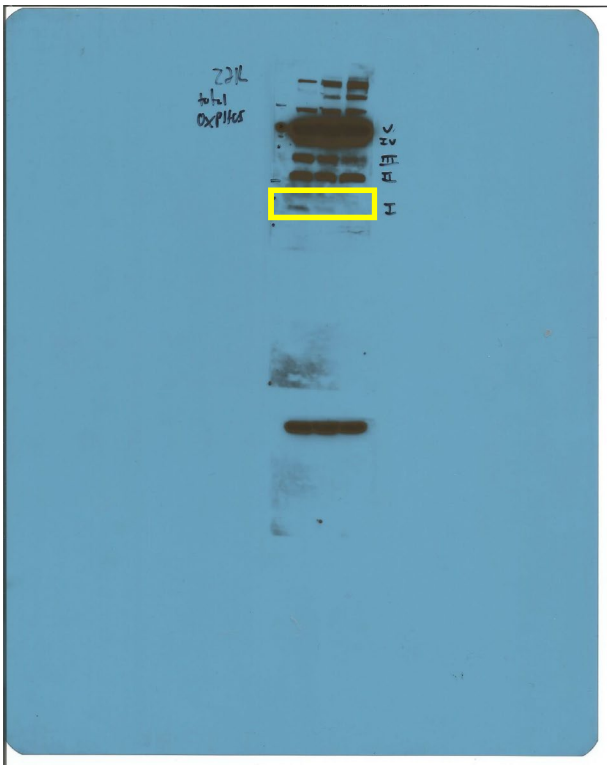

Figure S4, Panel C, Ab: Actin

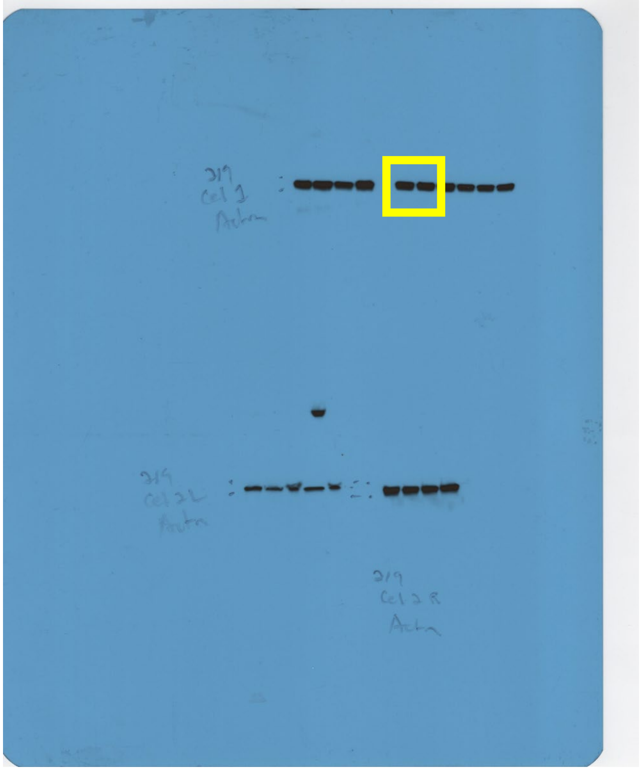

Figure S4, Panel C, Ab: HIF-2α

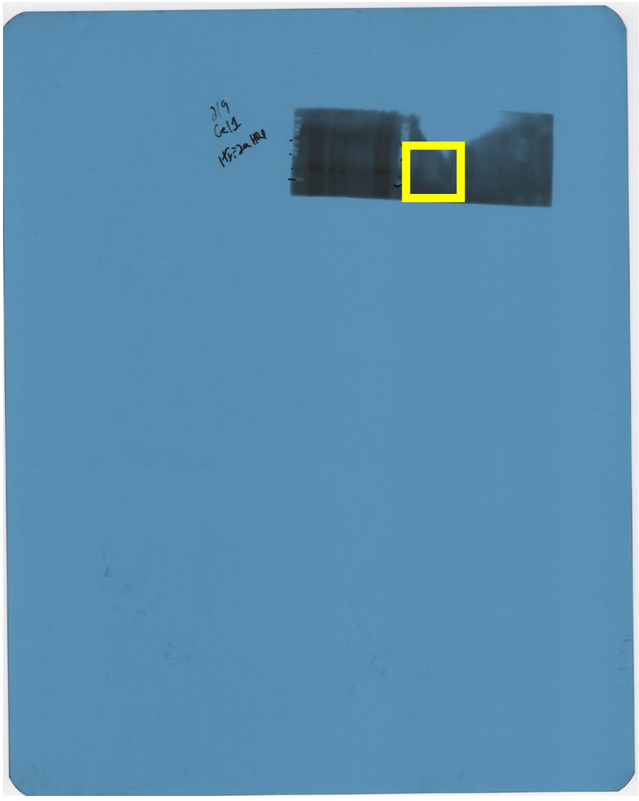

Figure S4, Panel C, Ab: ACSS2

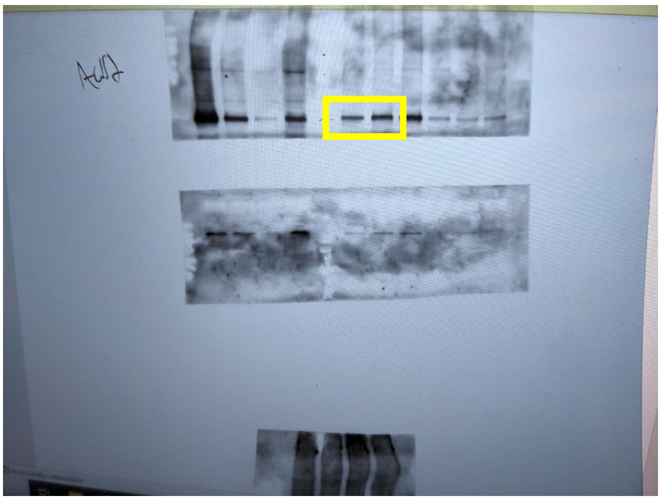

Figure S4, Panel C, Ab: GLUT1

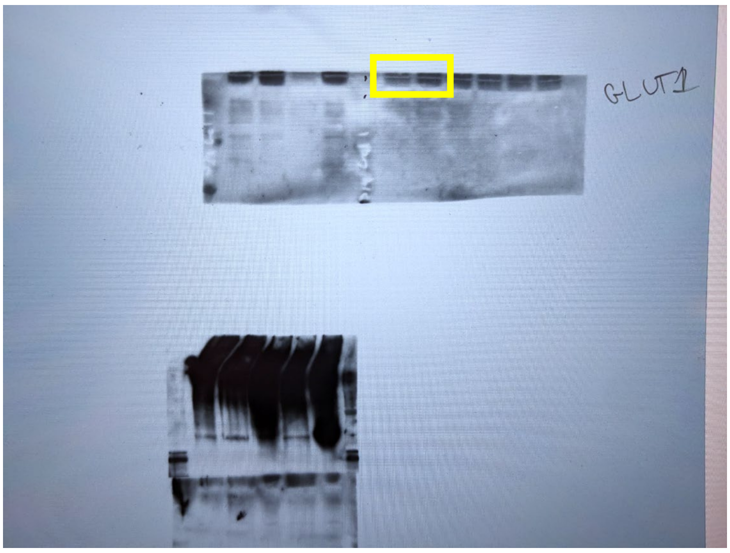

Figure S4, Panel D, Ab: OXPHOS

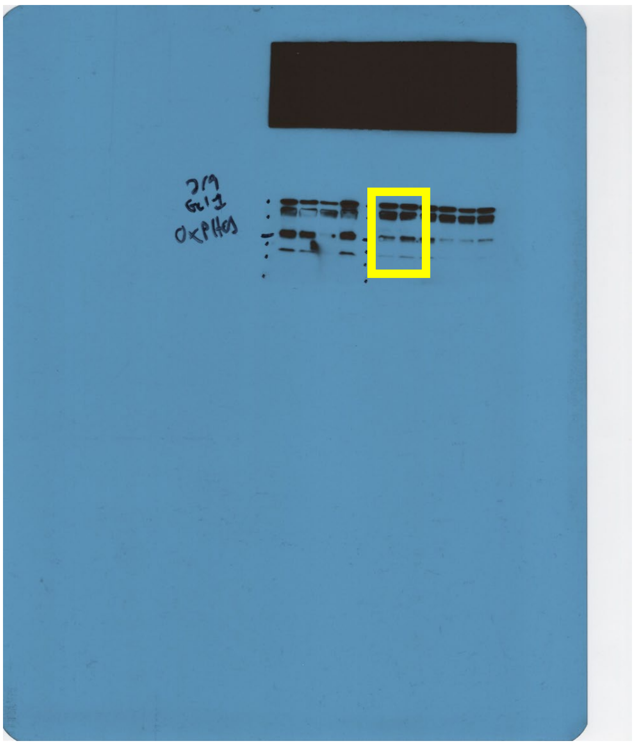

Figure S4, Panel C, Ab: Actin

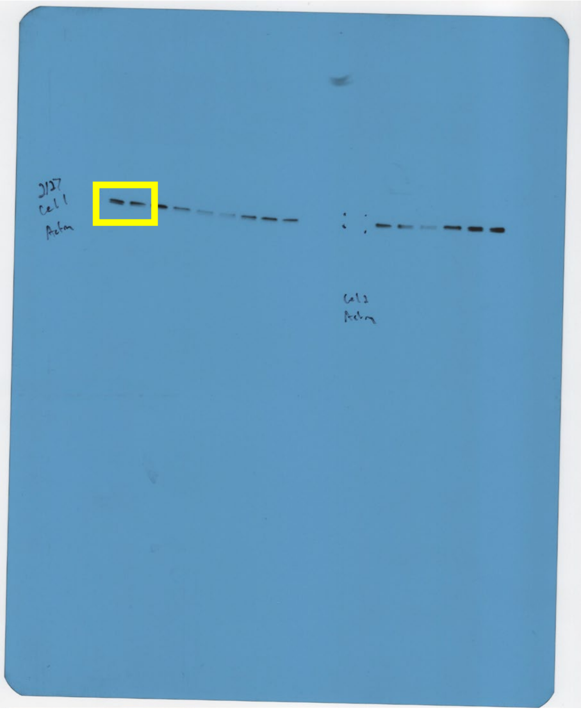

Figure S4, Panel C, Ab: HKII

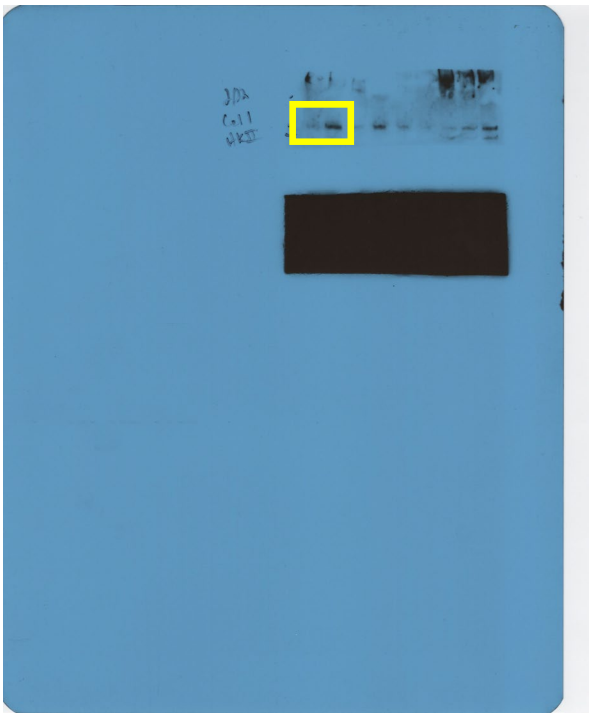

Figure 7

Figure 7, Panel B, Ab: Actin

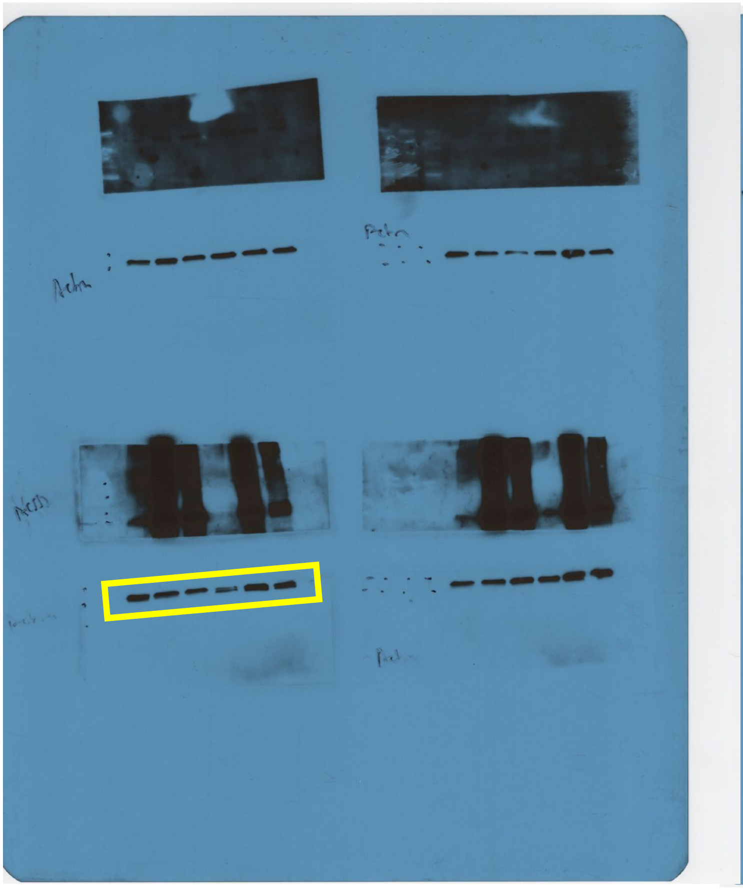

Figure 7, Panel B, Ab: HIF-2α

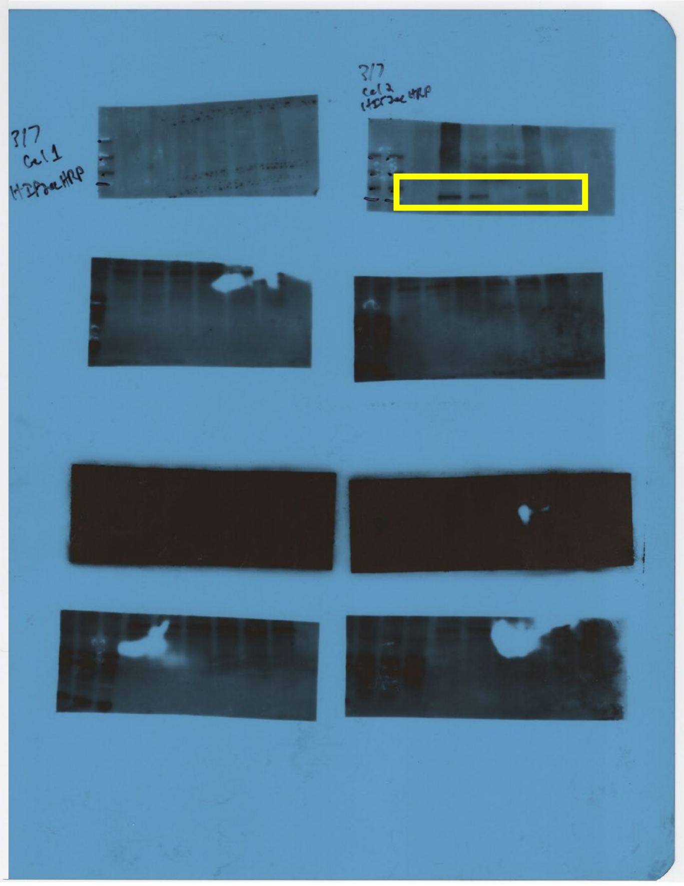

Figure 7, Panel B, Ab: ACSS2

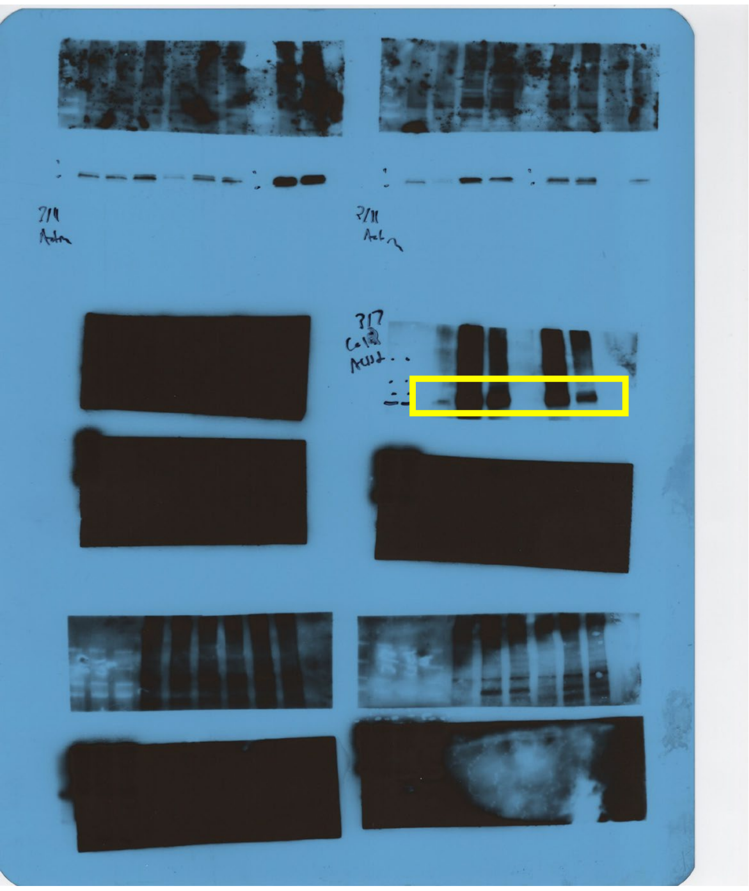

Figure 9

Figure 9, Panel A, Ab: Actin

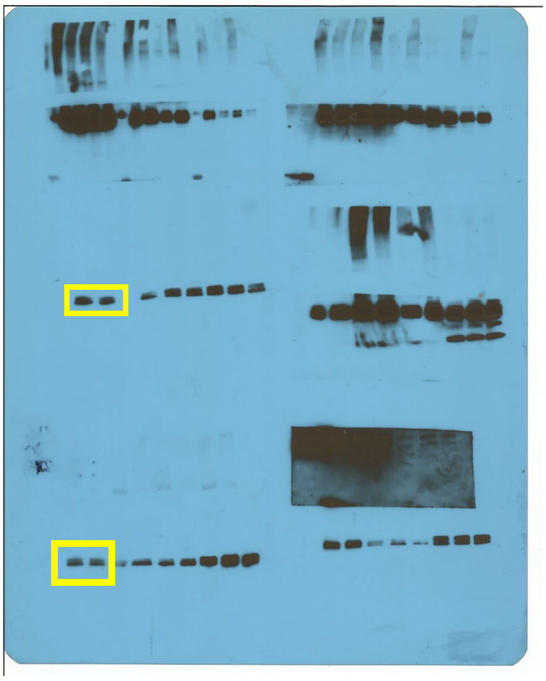

Figure 9, Panel A, Ab: E-Cadherin

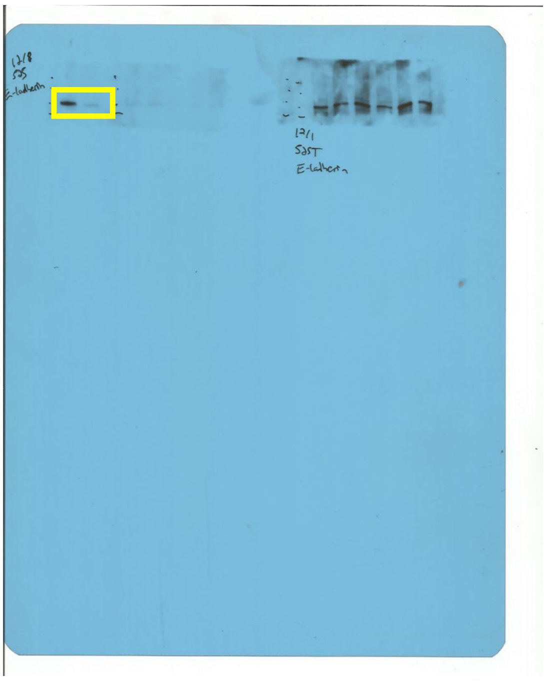

Figure 9, Panel A, Ab: HIF-2α

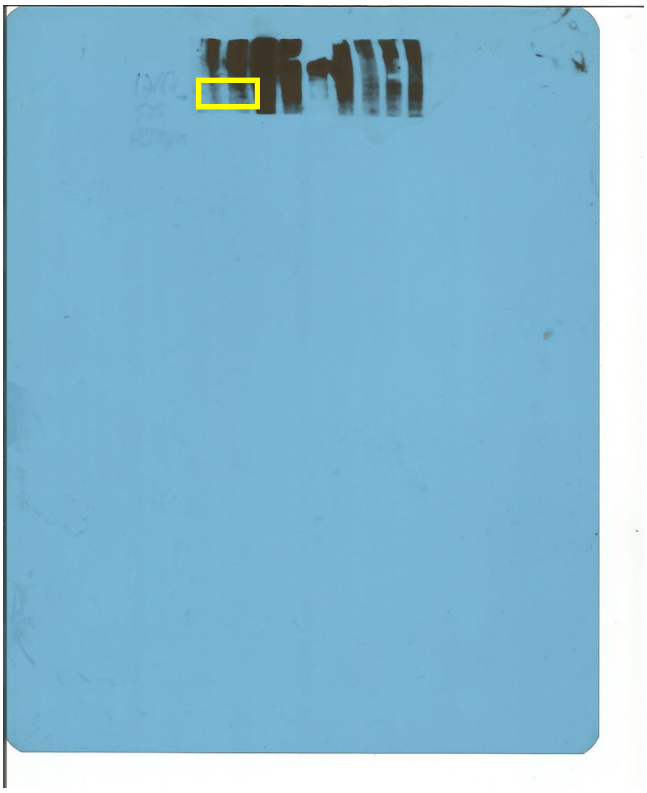

Figure 9, Panel A, Ab: pan-tubulin

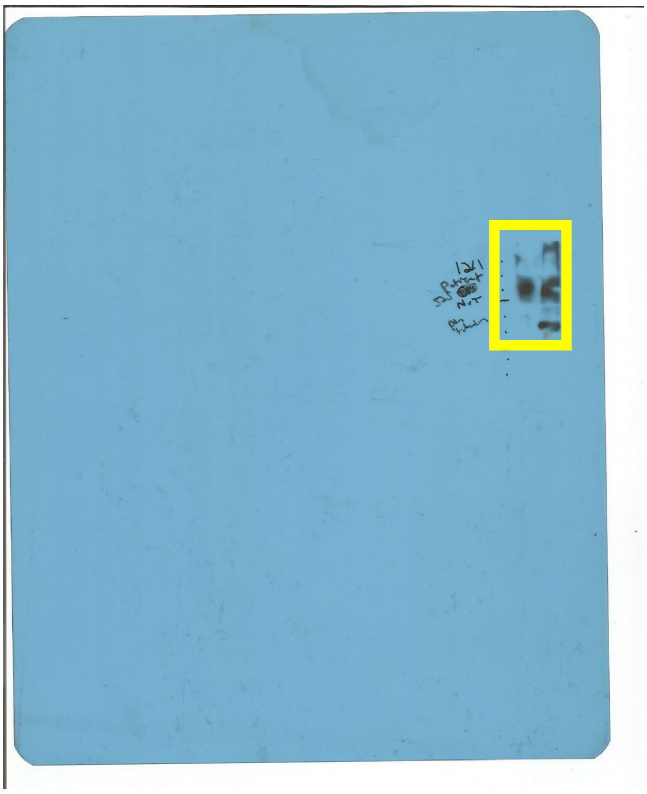

Figure 9, Panel A, Ab: AE1/AE3

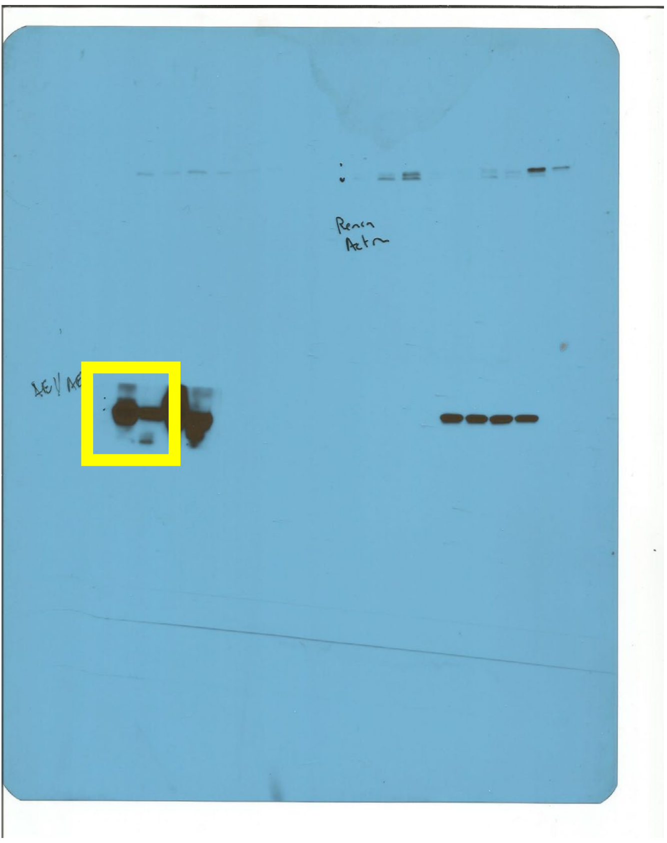

Figure 9, Panel A, Ab: Actin (parallel gel use)

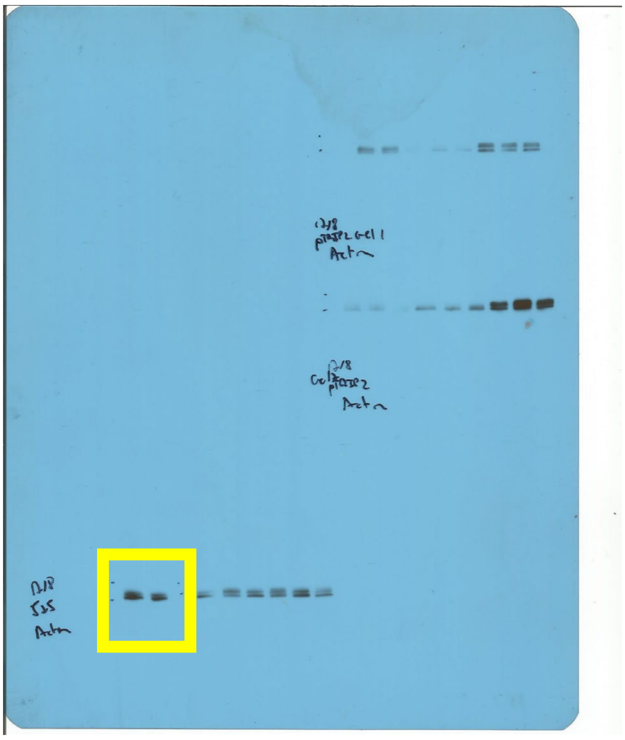

Figure 9, Panel B, Ab: HKII

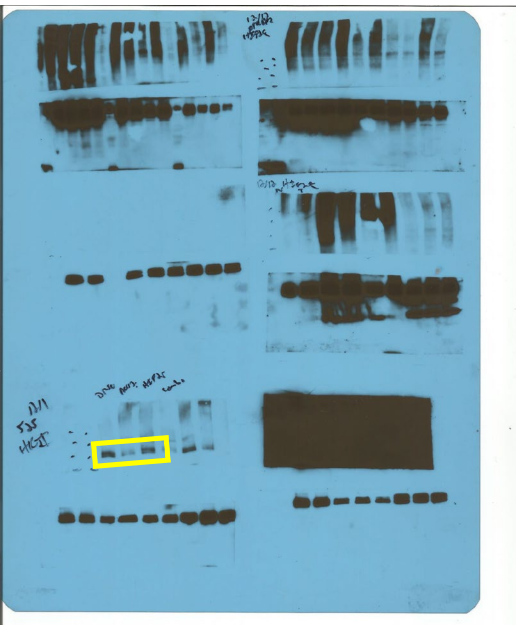

Figure 9, Panel B, Ab: HIF-2α

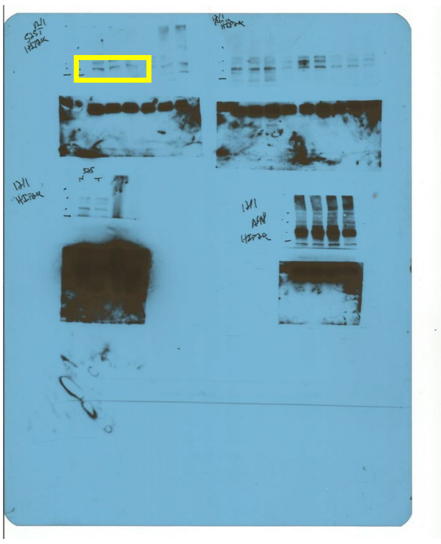

Figure 9, Panel B, Ab: ATP Synthase

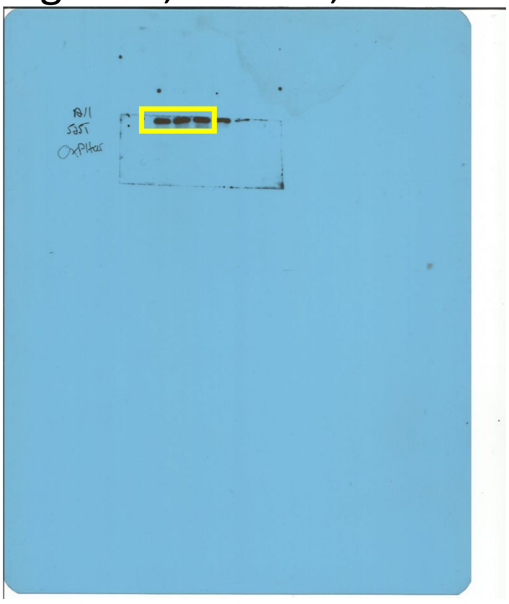

Figure 9, Panel B, Ab: AE1/AE3

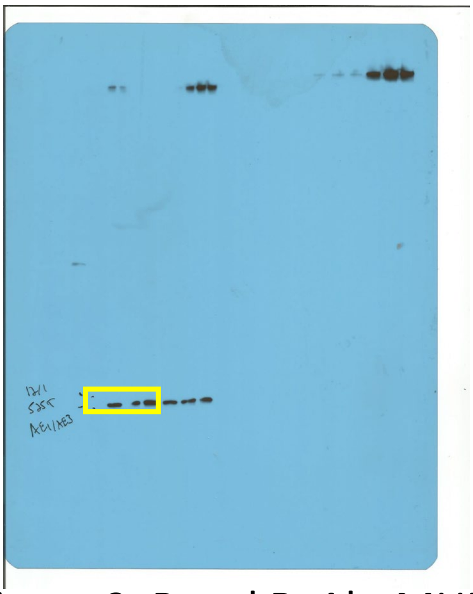

Figure 9, Panel B, Ab: MUL1

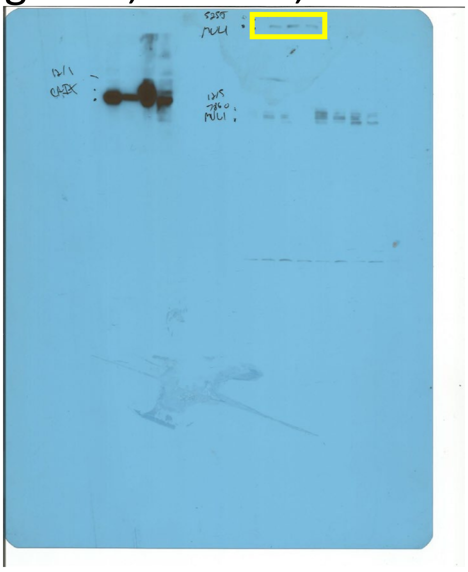

Figure 9, Panel B, Ab: Complex IV

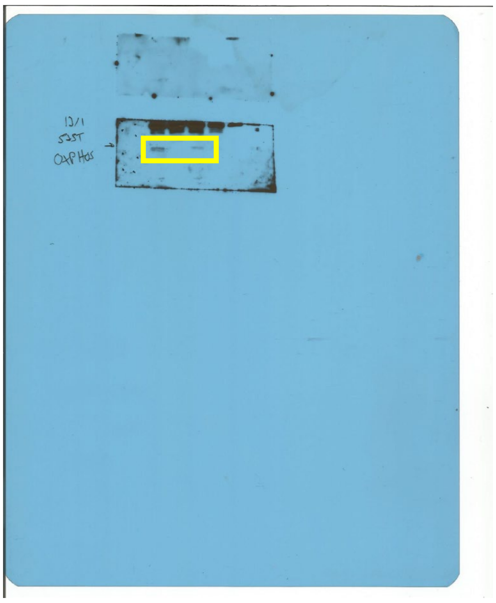

Figure 9, Panel B, Ab: E-cadherin

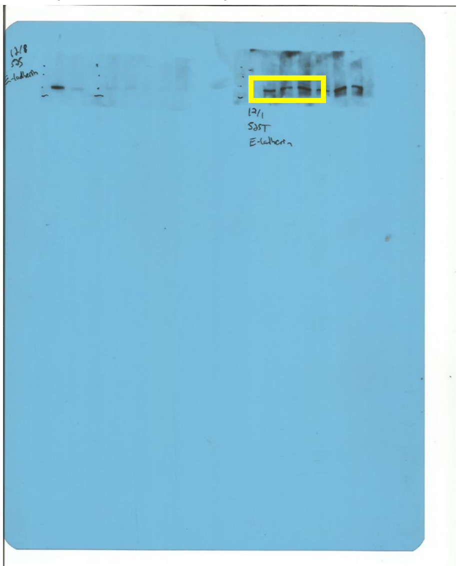

Figure S5

Figure S5, Panel B, Ab: HKII

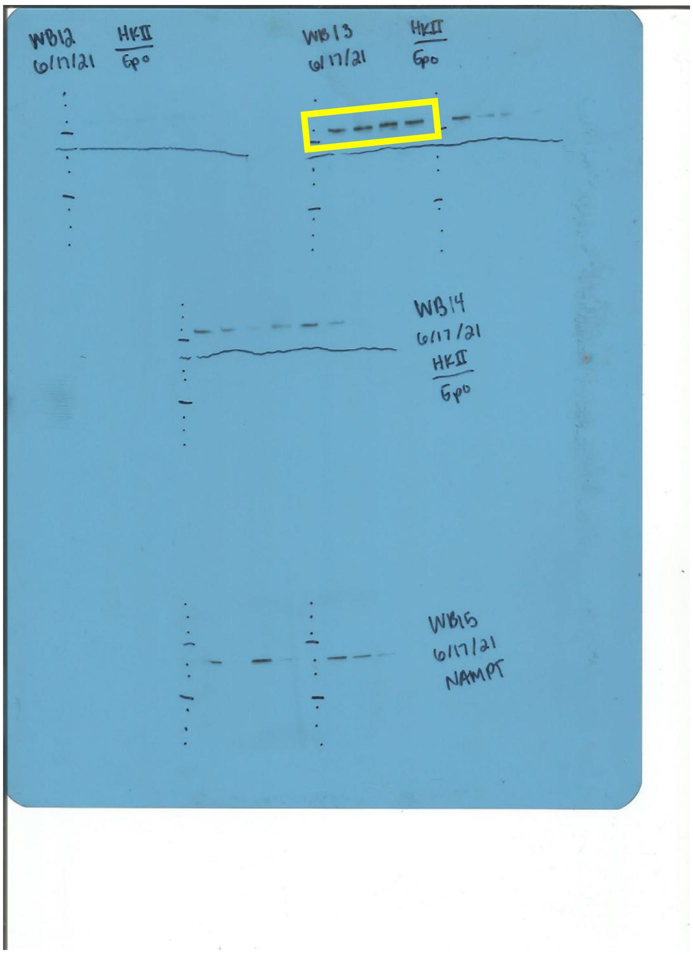

Figure S5, Panel B, Ab: HIF-2α

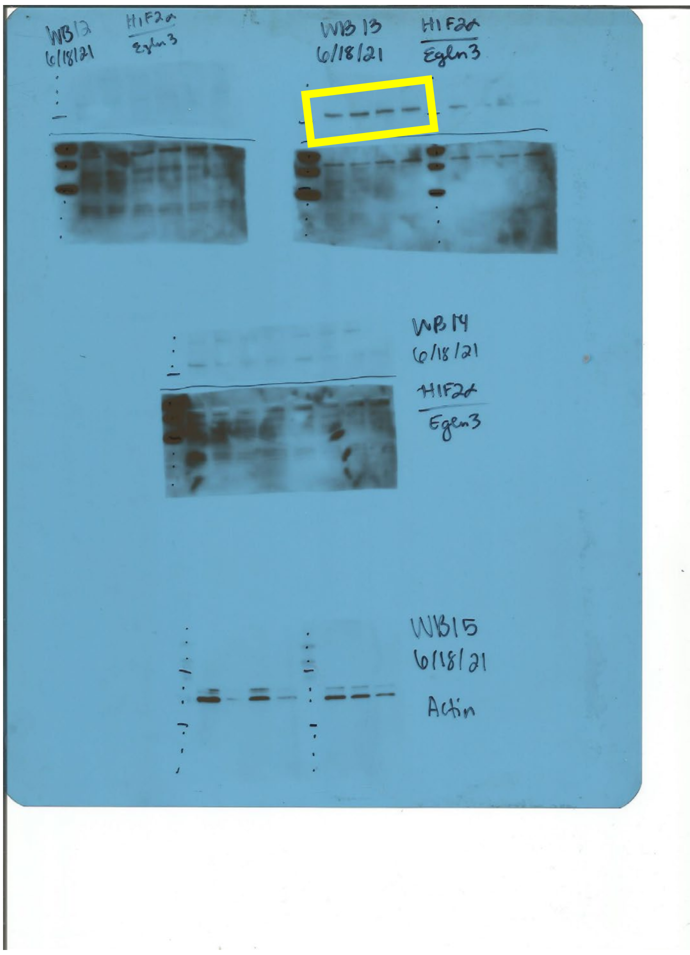

Figure S5, Panel B, Ab: Actin

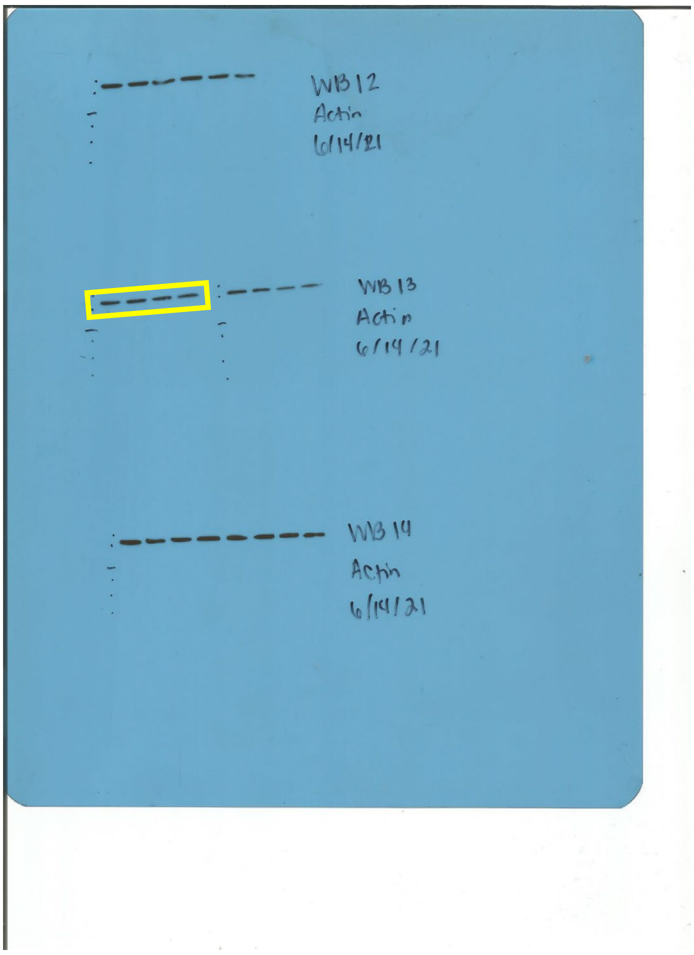

Figure S5, Panel D, Ab: HKII

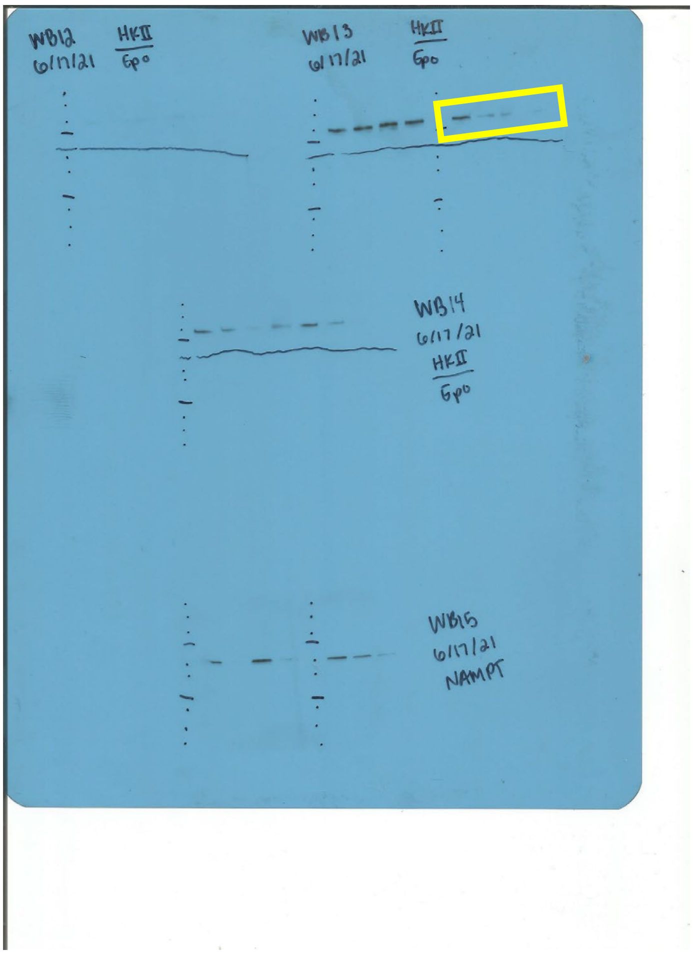

Figure S5, Panel D, Ab: HIF-2α

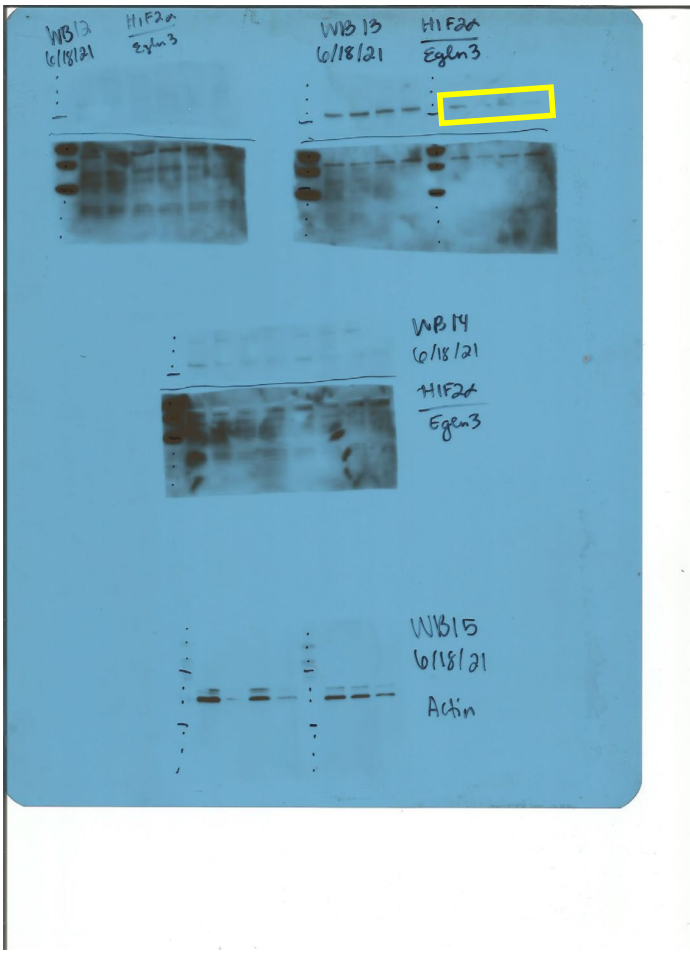

Figure S5, Panel D, Ab: Actin

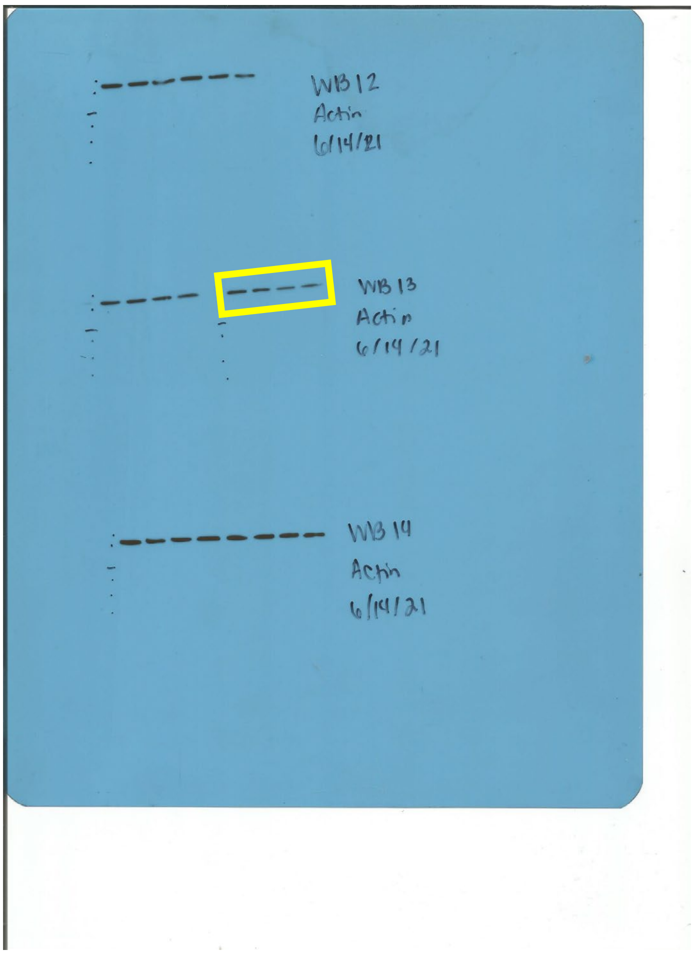

Supplement: Unedited blot and gel images [file jci-134-164249-s028.pdf]
